# Modified Hederagenin Derivatives Demonstrate Ex Vivo Anthelmintic Activity against *Fasciola hepatica*

**DOI:** 10.3390/pharmaceutics15071869

**Published:** 2023-07-03

**Authors:** Anand Chakroborty, Deiniol R. Pritchard, Marc E. Bouillon, Anna Cervi, Rolf Kraehenbuehl, Charlotte Wild, Caroline Fenn, Peter Holdsworth, Colin Capner, Gilda Padalino, Josephine E. Forde-Thomas, Joseph Payne, Brendan G. Smith, Maggie Fisher, Martina Lahmann, Mark S. Baird, Karl F. Hoffmann

**Affiliations:** 1 The Department of Life Sciences (DLS), Aberystwyth University, Aberystwyth SY23 3DA, UK; anand.chakroborty@ttuhsc.edu (A.C.); gip7@aber.ac.uk (G.P.); jef19@aber.ac.uk (J.E.F.-T.); 2 Department of Cell Biology and Biochemistry, Texas Tech University Health Sciences Center, 3601 4th Street, Lubbock, TX 79430, USA; 3 Naturiol Bangor Ltd., MSParc, Gaerwen, Anglesey LL60 6AG, UK; deiniol.rhodri@gmail.com (D.R.P.); annacervi@naturiol.uk (A.C.); 4 School of Natural Sciences, Bangor University, Bangor LL57 2UW, UK; marc.bouillon@endotherm.de (M.E.B.); r.kraehenbuehl@bangor.ac.uk (R.K.); lahmann@kth.se (M.L.); 5 Ridgeway Research Limited, Park Farm Buildings, Park Lane, St. Briavels, Gloucestershire GL15 6QX, UK; wild_charlie@hotmail.co.uk (C.W.); cfenn@ridgewayresearch.co.uk (C.F.); peter.paragon60@gmail.com (P.H.); colin.capner@colincapner.plus.com (C.C.); jpayne@ridgewayresearch.co.uk (J.P.); maggie@shernacre.co.uk (M.F.); 6 PAH Consultancy Pty Ltd., 3/27 Gaunson Crescent, Wanniassa, Canberra 2903, Australia; 7 School of Pharmacy and Pharmaceutical Sciences, Cardiff University, Redwood Building, King Edward VII Avenue, Cardiff CF10 3NB, UK; 8 Bimeda UK, Bryn Cefni Industrial Estate, Unit 2A, Llangefni LL77 7XA, UK; bgs@brendangsmith.com; 9 KTH Royal Institute of Technology, Biomedical Engineering and Health Systems, Hälsovägen 11, 141 52 Huddinge, Sweden

**Keywords:** *Fasciola hepatica*, anthelmintic drug discovery, *Hedera helix*, saponins and hederagenin

## Abstract

Infection with *Fasciola hepatica* (liver fluke) causes fasciolosis (or fascioliasis) and poses a considerable economic as well as welfare burden to both the agricultural and animal health sectors. Here, we explore the ex vivo anthelmintic potential of synthetic derivatives of hederagenin, isolated in bulk from *Hedera helix*. Thirty-six compounds were initially screened against *F. hepatica* newly excysted juveniles (NEJs) of the Italian strain. Eleven of these compounds were active against NEJs and were selected for further study, using adult *F. hepatica* derived from a local abattoir (provenance unknown). From these eleven compounds, six demonstrated activity and were further assessed against immature liver flukes of the Italian strain. Subsequently, the most active compounds (n = 5) were further evaluated in ex vivo dose response experiments against adult Italian strain liver flukes. Overall, MC042 was identified as the most active molecule and the EC_50_ obtained from immature and adult liver fluke assays (at 24 h post co-culture) are estimated as 1.07 μM and 13.02 μM, respectively. When compared to the in vitro cytotoxicity of MDBK bovine cell line, MC042 demonstrated the highest anthelmintic selectivity (44.37 for immature and 3.64 for adult flukes). These data indicate that modified hederagenins display properties suitable for further investigations as candidate flukicides.

## 1. Introduction

The liver fluke *Fasciola hepatica* (Linnaeus, 1758) causes fasciolosis or fascioliasis in herbivores [1,2]. Acute fasciolosis is often reported in ovine and caprine animals, whereas in bovine species the infection is normally chronic with blood loss, damage to the hepato-biliary system, and concomitant bacterial diseases [3,4]. Morbidity in livestock compromises qualitative and quantitative production of meat, fibre and milk [5]. Liver fluke infections in farm animals are increasing due to global climate change, resulting in increased survival rates of intermediate gastroropod hosts (e.g., *Galba trunculata*) as well as resistance to current treatment regimens [6,7,8,9].

In the United Kingdom alone, fasciolosis directly contributes to a loss of GBP 23 million/annum in the livestock industry [9]. Furthermore, according to World Health Organization (WHO) estimations, the global agricultural sector faces losses of USD 3.2 billion annually from liver fluke. Despite these enormous economic burdens, the budgets allocated for research and development are inadequate and the significance of fasciolosis has been largely neglected, especially in regards to its contributions to human disease. As a result, the WHO classified fasciolosis as a neglected disease (ND) [4,6,10].

Triclabendazole (TCBZ) is considered the front line flukicide treatment for fasciolosis (in animals as well as human infections), owing to its broad-spectrum efficacy against all three parasite stages (newly excysted juveniles (NEJs), immature flukes and adult flukes) that cause damage to the definitive host [11,12]. TCBZ is a halogenated benzimidazole thiol derivative and has been on the market for over 20 years. Cases of TCBZ resistant liver flukes in ruminants have been scientifically documented in Australia, the UK, Ireland and other European countries; this treatment vulnerability is of concern for sustaining liver fluke control in the long term [12,13,14].

Botanical-derived compounds harbouring potential parasiticidal properties have been tested as therapeutic agents against both protozoan and metazoan pathogens [15,16,17,18,19]. The anti-malarial compound artemisinin is a striking example of a therapeutic phytochemical; moreover, several natural terpenoid molecules have been studied against parasitic trematodes and their anthelmintic properties have been evaluated [17,20,21,22].

Among the most common botanical derived bioactive components are saponins [23]. English or Common Ivy (*Hedera helix*, Linnaeus 1753), a member of the Araliaceae family, is abundant throughout the British Isles [24]. The fruit and leaf extracts contain large quantities of triterpenoid saponins with broad therapeutical properties, including anti-leishmanial, anti-microbial, anti-fungal, anti-viral, molluscicidal, anti-proliferative and anti-inflammatory effects [25,26,27,28,29].

**Figure 1:**
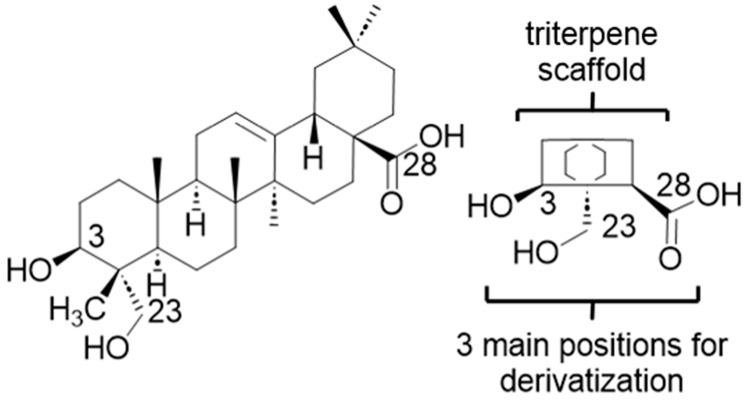
Hederagenin and its short-hand structure showing the three main positions used for derivatization. The full structure (**left**) and a simplified template (**right**) of hederagenin are illustrated, showing the three functional groups targeted for derivatization.

*H. helix* derived saponins are triterpenoid glycosides, unlike the steroid glycosides seen in monocotyledonous angiosperms [15]. Triterpenoid saponins are further classified as either monodesmosidic or bidesmosidic, depending on the number of carbohydrate substituents on the aglycone (the sapogenin or triterpenoid skeleton) [24]. The saponins of Common Ivy are largely based on a single sapogenin, hederagenin (Figure 1), with much smaller quantities based on oleanolic acid.

α-Hederin, a major hederagenin based mono-desmosidic saponin extracted from *H. helix*, shows cytotoxic effects against tumour cells, possesses haemolytic activity and induces membrane permeabilization [24,26]. It also demonstrates lethal activity against *F. hepatica* and members of the *Dicrocoelium* genus [30]. Hydrolysis of ivy saponins, including α-hederin, can be used to produce significant quantities of hederagenin (5–10% of dry weight). It was thus of interest to determine whether chemical derivatization of hederagenin could lead to the production of analogues with activity against different *F. hepatica* lifecycle stages, thereby identifying a potential new cost effective semi-synthetic treatment for controlling fasciolosis.

## 2. Materials and Methods

### 2.1. Ethics Statement

Adhering to the United Kingdom Home Office Animals (Scientific Procedures) Act of 1986 as well as the European Union Animals Directive 2010/63/EU (approved by RRL Animal Welfare and Ethical Review Bodies), the *F. hepatica* (Italian strain) [31] lifecycle was maintained in sheep under project licenses P6D805744 and PA09B4E45.

### 2.2. Modified Plant Saponins from H. helix

**Figure 2:**
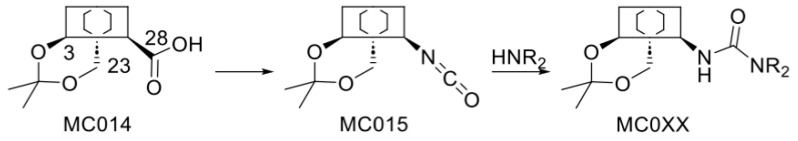
Synthesis of the ureate derivative from protected hederagenin. The acetal protected hederagenin (MC014) was converted into the isocyanate (MC015) using diphenylphosphoryl azide in toluene following a known procedure (Methods S1). Addition of a set of amines then gave the different ureates (MC0XX).

The structures of all modified hederagenins (MC and IVL series) used in this work are shown in Table S1. The synthesis of some of these compounds, from hederagenin, has been reported before [15,32,33,34]. The syntheses of other compounds, as well as their proton and carbon NMR spectra, are provided in Methods S1 and NMR Spectra S1 (Supplementary Materials). A large set of these compounds were prepared from hederagenin protected as the acetal MC014 by conversion into the isocyanate MC015, followed by reaction with selected amines (Figure 2).

### 2.3. Parasites and Dose Preparation

The laboratory reared *F. hepatica* Italian strain was originally isolated from the Campania region of Southern Italy in 2014 [31]; this strain is susceptible to TCBZ and has been used in our previous investigations [20,21,22]. To maintain the liver fluke lifecycle, cercariae were harvested from *Galba truncatula* snails, which had previously been infected with miracidia. Cercariae were allowed to encyst onto Visking tubes, which led to the production of metacercariae. Metacercariae were examined for viability and suspended in a 0.2% agar (Sigma-Aldrich, UK) solution to produce a final peroral dose of 200 metacercariae per sheep. At postmortem (12 weeks post infection), eggs were harvested from adult liver fluke, incubated and hatched to produce miracidia, thereby completing the liver fluke lifecycle.

In this investigation, immature (4-weeks old) and adult (8-weeks old) Italian strain flukes used for ex vivo assays were produced by infection of 6-month-old Texel Mule X lambs. All metacercariae, immature and adult flukes were supplied by Ridgeway Research Ltd. (Gloucestershire, UK) and subsequently transported to Aberystwyth University for ex vivo experiments. Assays initiated with wild strain adult *F. hepatica* used flukes derived from infected livers of sheep and cattle at Randall Parker foods, Llanidloes, Wales (UK).

### 2.4. Ex Vivo Assays on Life Stages of F. hepatica (Italian or Wild Strain) Parasites

**Figure 3:**
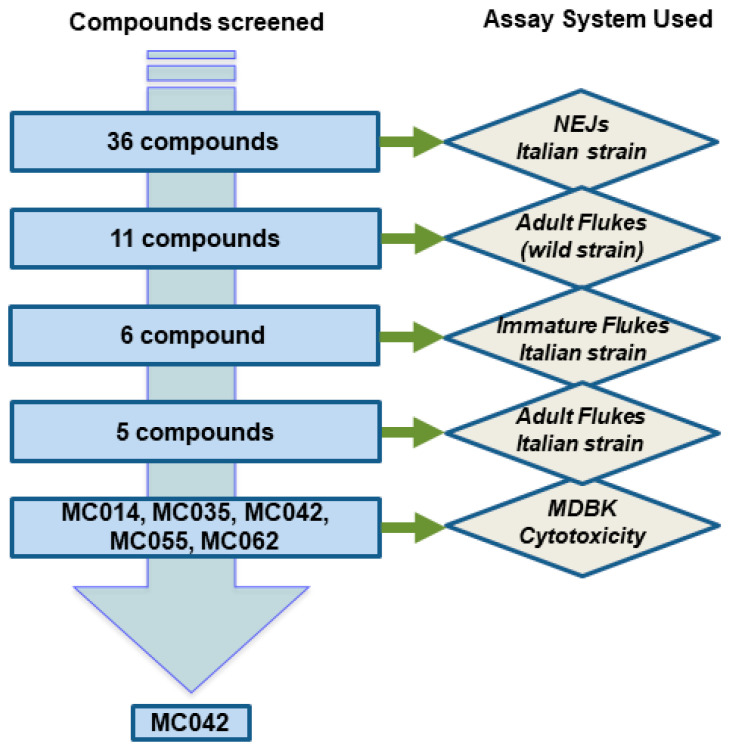
Flowchart describing the screening strategy for *H. helix* derived modified hederagenins. The initial 36 compounds were passed through a series of assays; the first four assays consisted of co-culturing liver flukes ex vivo with different concentrations of compound (10 µM or 40 µM for single concentration assays; 40 µM, 13.3 µM and 4.4 µM for dose response assays). The laboratory reared *F. hepatica* Italian strain was originally obtained from Campania, Italy, whereas the *F. hepatica* wild strain (provenance unknown) was obtained from Randall Parker Foods, Llanidloes (Wales), UK. The final assay was used to assess overt cytotoxicity on the bovine MDBK cell line.

Screening of the hederagenin derivatives was performed against three life stages of *F. hepatica* following the strategy described in Figure 3.

#### 2.4.1. Effect against Newly Excysted Juveniles (Italian Strain)

Initially, the candidate compounds’ effect on 25 Newly Excysted Juveniles (NEJs) was assessed following 72 h of compound/parasite co-incubation. The NEJs were excysted from metacercariae [21] and assayed at 10 μM (in 0.1% DMSO) final concentration alongside negative (0.1% DMSO) and positive (10 μM TCBZ, in 0.1% DMSO) controls as previously described [22]. The MC and IVL compound series were screened separately.

The motility (1—good/normal; 2—moderate; 3—low; 4—very little; 5—no movement) and phenotypic (ultrastructural changes) scores were noted according to a previously described scoring matrix [17] (Figure S1 and Table S2). Eleven molecules (hit compounds) were identified for further evaluation on adult and immature flukes as they demonstrated the strongest effects on both NEJ phenotype and motility metrics (green and yellow rows, Figure S1). Bright field microscopic images (20× magnification, using an Olympus CK2 microscope with top-mounted Euromex HD ultra-camera) were subsequently taken of NEJs following treatment with five (amongst the eleven) of the most active molecules (MC014, MC042, MC035, MC055 and MC062) at 10 µM for 72 h (Figure S2).

#### 2.4.2. Activity against Adult Liver Flukes (Wild Strain)

Due to the high number of active molecules and a shortage of Italian strain adult liver flukes, the eleven hit compounds were screened for 72 h at 40 µM (in 0.4% DMSO) on wild strain adult *F. hepatica* (n = 4/condition) of unknown provenance (Table S3). The protocol for culturing and scoring (motility) the activity of these compounds was previously described (1—good/normal; 2—moderate; 3—resting (less than 10 s pulses of body and head), 4—apathetic (less than 2 s pulses of body and head), 5—paralysis and 6–no movement or dead worms) [20]. Six compounds were deemed active in these assays and were selected for further screening.

#### 2.4.3. Activity against Immature Liver Flukes (Italian Strain)

The six selected compounds (at 40 µM in 0.4% DMSO) were evaluated on immature flukes (n = 4/condition) at 24, 48 and 72 h (Table S4). The protocol for culturing and scoring (motility) of these life stages have been previously described [20,21]. Due to restricted numbers of immature worms, a dose response assay was conducted only for MC042. Here, immature worms (n = 2/concentration tested) were screened against descending concentrations of MC042 (40 µM, 13.3 µM and 4.4 µM); motility scores were recorded at 24, 48 and 72 h (Table S5). The scoring pattern used is identical to that described for NEJs.

#### 2.4.4. Dose Response Studies on Adult Liver Flukes (Italian Strain)

The collection, ex vivo culturing and screening of adult liver fluke (Italian strain; n = 3/condition) was previously described [20,21]. Dose response assays (40 µM, 13.3 µM and 4.4 µM) were conducted for MC014, MC042, MC035, MC055 and MC062 with motility scores recorded at 24, 48 and 72 h (Table S6).

### 2.5. MTT Assay on MDBK Cells

MTT cell viability assays were performed on Madin–Darby bovine kidney (MDBK cells) for the five selected hederagenin derivatives (MC014, MC035, MC042, MC055 and MC062) using final concentrations of 100 μM, 75 μM, 50 μM, 25 μM, 10 μM, 5 μM and 1 μM (in 1% DMSO). Negative (1% *v*/*v* Triton X-100, Sigma-Aldrich, UK) and positive (1% *v*/*v* DMSO or media only) controls were included. The protocol was followed as previously described [20,21,22]. Absolute EC_50_ values were calculated from the corrected absorbance of replicates by non-linear regression, after log transformation of concentrations and data normalization (where DMSO values represents 0% inhibition and TX-100 values represent 100% inhibition) using GrapPad Prism 8.

### 2.6. Statistics

Statistical analyses of the data were performed using GraphPad Prism 9.5 software (https://www.graphpad.com/scientific-software/prism/, accessed on 18th June 2023) to determine significant differences amongst population means. As the scores were not equally distributed, a non-parametric test (Kruskal-Wallis), followed by a multiple comparisons test (either Dunn’s test or uncorrected Dunn’s test) was conducted; *p*-values ≤ 0.05 were considered statistically significant.

## 3. Results

Initially 36 compounds were produced from hederagenin. The structures and their method of synthesis are presented in the Supplementary Material (Table S1).

**Figure 4:**
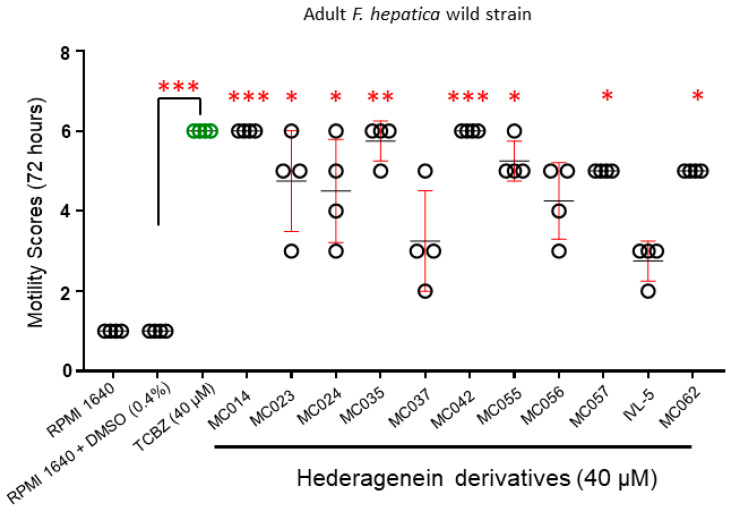
Hederagenin derivatives differentially affect adult *F. hepatica* (wild strain) viability and motility. Adult *F. hepatica* flukes (wild strain; n = 4) were co-cultured with TCBZ (40 µM in 0.4% DMSO), the DMSO solvent (0.4%), medium only (RPMI 1640) or the 11 prioritized hederagenin derivatives (40 µM in 0.4% DMSO) for 72 h. Each parasite was carefully observed using bright field microscopy and scored accordingly (from 1 for normal movement to 6 for no movement or death). The individual fluke score is represented by a circle, means of the population are represented in black and error bars are represented by red, and green circles represent population of TCBZ treated parasites only. Statistically significant differences in compound-mediated motility were calculated by multiple comparisons (uncorrected Dunn’s test) to adult flukes co-cultured in 0.4% DMSO. *, ** and *** indicate *p* < 0.05, *p* < 0.001 and *p* < 0.0001, respectively.

To determine their flukicidal properties, each compound was screened at 10 µM concentration against NEJs, the major infective stage for fasciolosis (Table S2 and Figure S1). Ex vivo co-culture of the compound/NEJs for 72 h enabled phenotypic alterations and motility to be independently quantified [20]. Compounds that simultaneously induced both tegumental damage/other ultrastructure changes (Figure S1a,b) and arrested motility (Figure S1c,d) were considered hits. From the set of 36 molecules, 11 (MC014, MC023, MC024, MC035, MC037, MC042, MC055, MC056, MC057, MC062 and IVL-5; highlighted by green and yellow rows, Table S2) showed maximal alterations to both phenotype/ultrastructure and motility at 72 h post-initiation of co-culture; these effects were similar to those induced by TCBZ. Indeed, the alterations to NEJ phenotype/ultrastructure and motility induced by these 11 molecules were also visible as early as 24 h post co-culture (Table S2); therefore, these molecules were progressed for screening on adult liver flukes (Figure 4). The rest of the compounds failed to uniformly affect NEJ phenotypes and motility after 72 h co-culture and were subsequently excluded from further assays.

Of the eleven compounds progressed, MC014 and MC042 proved active against all ex vivo cultured adult liver flukes (provenance unknown) collected from condemned bovine/caprine livers; all adults demonstrated no motility after 72 h, which was comparable to adult flukes co-cultured with TCBZ (Figure 4, Table S3). While MC035, MC055, MC057 and MC062 treated liver flukes experienced partial paralysis, this effect was reversible (i.e., movement slightly recovered when flukes were immersed in fresh culture media). Nevertheless, adult fluke movement was significantly affected when compared to control worms (0.4% DMSO in culture media) at 72 h and justified these compounds’ inclusion for further investigations. MC023, MC024, MC037, MC056 and IVL-5 exposed liver flukes displayed less severe motility defects and were not prioritized for further anthelmintic screening.

**Figure 5:**
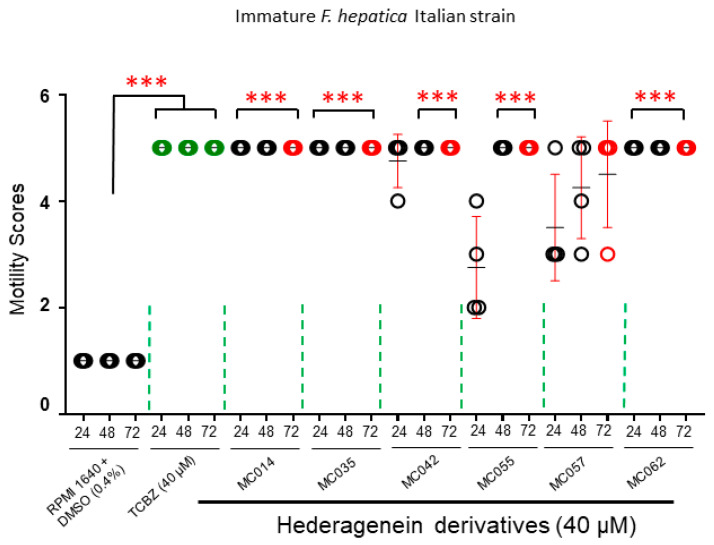
Prioritized hederagenin derivatives kill *F. hepatica* (Italian strain) immature flukes. Immature *F. hepatica* flukes (n = 4) were co-cultured with TCBZ (40 µM in 0.4% DMSO), medium containing the DMSO solvent (0.4%) or the six prioritized hederagenin derivatives (40 µM in 0.4% DMSO) for 72 h. Each parasite was carefully observed using bright field microscopy and scored accordingly (1 for normal movement, 5 for no movement or death). Each parasite was scored at three time points: 24, 48 and 72 h. Except for MC057, all parasites treated with TCBZ (green circles) and modified saponins were completely immobile by 72 h of co-incubation. Significance was calculated by multiple comparisons (Dunn’s test) with an individual fluke score represented by a circle, means of the population are represented in black and error bars are represented by red. Statistically significant difference in motility, compared to adult flukes co-cultured in 0.4% DMSO, are indicated (***, *p* < 0.0001).

The prioritized six compounds (MC014, MC035, MC042, MC055, MC057 and MC062) were subsequently evaluated for their effects on immature fluke (Italian strain) motility (Figure 5, Table S4). With the exception of MC057, all compounds paralyzed the assayed immature flukes by 72 h post-co-culture.

**Figure 6:**
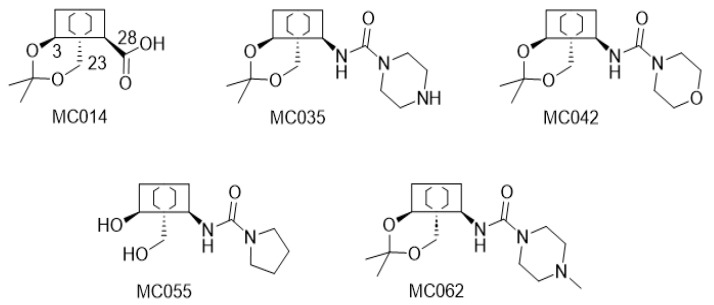
Structures of the five lead molecules selected for dose response studies. MC014 is the acetonide of hederagenin. The four remaining molecules are ureates derived by addition of particular cyclic secondary amines to isocyanate MC015, as shown in Figure 2.

**Figure 7:**
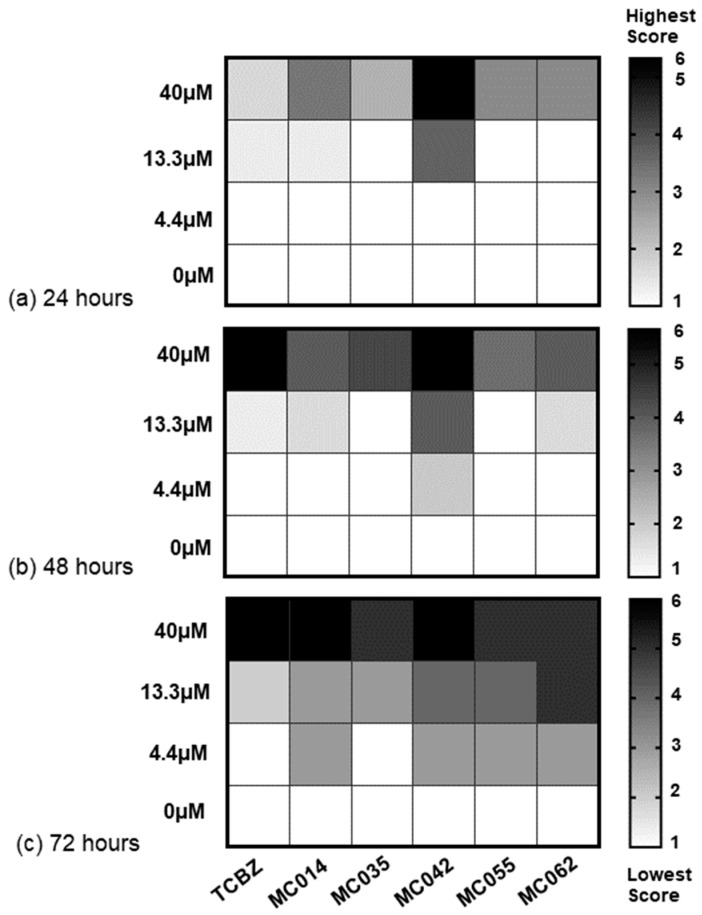
Dose response titrations of MC014, MC035, MC042, MC055 and MC062 on adult *F. hepatica* flukes (Italian strain). The heat maps represent means of dose response scores (40 µM, 13.3 µM, 4.4 µM and 0 µM) of adult *F. hepatica* flukes (Italian strain; n = 3) assayed at: (**a**) 24 h, (**b**) 48 h and (**c**) 72 h. White squares represent the lowest motility score (1) and black squares represent the highest motility scores (6). Squares containing grey scales represent mean of intermediate motility scores.

Following these assays with immature liver flukes, dose response studies (40 μM, 13.3 μM, 4.4 μM) on adult flukes (Italian strain) were performed with the five remaining molecules (MC014, MC035, MC042, MC055 and MC062, Figure 6). These results are presented as heat maps in Figure 7 (data in Table S6). After 72 h, two out of three adult flukes were completely immobilized after exposure to MC042 (40 μM), with the remaining worm exhibiting severe paralysis. When compared to controls (DMSO, 0.4%), adults co-cultured with MC042 at lower concentrations (13.3 μM and 4.4 μM) also exhibited impaired movement after 72 h. Decreased movement in adult flukes was additionally noted with the other four compounds (MC014, MC035, MC055 and MC062) during dose-response assays, but their activities were not as fast-acting when compared to MC042.

To investigate the general cytotoxic effects of the five prioritized compounds, dose response titrations were next conducted on Madin–Darby bovine kidney (MDBK) cells. Subsequently, the selectivity indices (SI = CC_50_ in MDBK cells/EC_50_ on *F. hepatica* life stages) for four of the five hit compounds were calculated after 24 h exposure (Table 1). Notably, the SI could not be obtained for MC014 as this compound was not cytotoxic even at the highest concentration tested (100 μM). Instead, MC014 induced a modest proliferative effect. MC014 was, therefore, excluded from further consideration because of the potential link between cell proliferation and carcinogenesis [35]. Collectively, these data supported the selection of MC042 as the most promising hederagenin amongst the 36 original compounds tested. In summary, MC042 displayed anthelmintic potencies of 1.07 µM and 13.02 µM against immature and adult flukes, respectively. When cytotoxicity was considered, MC042 demonstrated immature and adult fluke selectivities of 44.37 and 3.64, respectively (Table 1 and Table S7).

## 4. Discussion

### 4.1. The Compounds Evaluated

Partly due to the presence of detergent like properties, natural plant saponins have been exploited for diverse therapeutic applications. In past studies, we have investigated the anthelminthic properties of plant derived saponins, isolated from Fir species commonly found in Welsh forests [22]. Because of this and, additionally due to previous reports documenting the anthelmintic properties of saponins derived from of other plant species [36,37,38,39,40], we pursued the characterization of saponin analogues chemically derived from a common and easy to obtain plant species, *H. helix* (Common Ivy).

Common Ivy, a very widespread non-food plant, is unusual in that its saponins are almost entirely based on a single sapogenin, hederagenin (Figure 1), which can be isolated on a large scale (5–10% of dry ivy weight of leaf or fruit) by acidic hydrolysis of plant extracts. While hederagenin is a relatively large chemical compound, modifications can relatively easily be achieved by chemical reactions at either the diol (positions 3, 23) or the carboxylic acid (position 28) found on opposite sides of the compound. It was thus of interest to determine whether chemical derivatization of hederagenin could enhance the activity against different stages of *F. hepatica*, to provide a new cost-effective semi-synthetic treatment.

Some 36 compounds were prepared from hederagenin by chemical transformations. A large number of these compounds were prepared from hederagenin by initial protection of the diol group as an acetal to create the acetonide MC014, followed by conversion into the isocyanate MC015. This intermediate was reacted with a series of amines to result in a set of seventeen urea derivatives and two carbamates (Table S2). It is known that urea analogues of this type (i.e., based on the dehydroabietane skeleton) are active against both *F. hepatica* [20] and *Leishmania* sp. [15]. The remaining compounds display primarily modifications at the diol site, mimicking to various extent the original carbohydrate section, and an unmodified or esterified carboxylic acid residue. Others are derivatives in which the 28-acid has been reduced to an alcohol.

### 4.2. Effect of Compounds on Different F. hepatica Life Stages

As TCBZ is the only commercially available drug effective against all intra-mammalian developmental stages of *F. hepatica* (including NEJs), eleven out of the 36 semi-synthetic saponin derivatives (MC014, MC023, MC024, MC035, MC037, MC042, MC055, MC056, MC057, MC062 and IVL5) were chosen for further progression as they all showed comparative activity to TCBZ on NEJs (n = 25/treatment) during initial ex vivo assays (Figure S1 and Table S2). A proportion (~70%) of the original 36 compounds did not achieve the same level of activity as TCBZ and were, therefore, discarded from further investigation at this stage. Follow-on ex vivo assays against wild strain adults (n = 4/treatment) reduced the number of anthelmintic candidates with both NEJ and adult worm activities from eleven to six (MC014, MC035, MC042, MC055, MC057 and MC062, Figure 4). After testing these six compounds against immature flukes (n = 4/treatment), MC057 was the least active in this series and was removed from follow-on dose-response titrations on adult Italian strain flukes (n = 3/treatment). Of the remaining five compounds, MC014 and MC042 showed the most promise with both compounds achieving biocidal effects on all immature and adult flukes after 72 h of ex vivo co-culture. While three further compounds (MC035, MC055 and MC062) caused reversible paralysis, they were less potent than MC014 and MC042. Of the 36 starting molecules, compound MC042 was identified as the most promising anthelmintic hederagenin derivative due to its SI (44 and 4, immature and mature flukes, respectively), while MC014 was removed because of its proliferative effect on MDBK cells.

The underlying mechanisms of action of these compounds are unknown, but hederagenin triterpenoid saponins have been shown to increase the permeability of cell membranes [24,41], reduce mitochondrial potential [42] and inhibit vital enzymatic activities [43]. In protozoan parasites, hederagenin formulations interfere with mitochondrial activity by decreasing ATP levels, depolarizing membrane potential and inducing over production of reactive oxygen species [44]. Due to these and other reported triterpenoid saponin-mediated biological activities [45], the ultrastructural damage observed on NEJ surfaces upon co-culture with MC014, MC035, MC042, MC055 and MC062 membranes was unsurprising. In fact, similar phenotypes have previously been observed on *F. hepatica* membranes when examining the anthelmintic activities of related terpenoids [17,20,21,22]. The synthetic hederagenin analogues may also be acting on targets that control movement of worms in addition to alterations of tegumental surface membranes as evidenced by inhibition of NEJ, immature and mature fluke motility. These molecules may also inhibit protein synthesis pathways as discussed in Whiteland et al. [22]. Nevertheless, it is currently unclear how these semi-synthetic hederagenin derivatives lead to the *F. hepatica* phenotypes reported within this study.

### 4.3. Structure-Activity Relationship Investigations

Amongst the 36 compounds, broadly subdivided in 10 classes as described below, eleven had activity against NEJs (Table S2). While these eleven were selected for further ex vivo studies, all 36 were subjected to structural activity relationship investigations detailed below.

1. The ureates (Table S2; groups 6,7) form the largest group of compounds studied, with 17 examples. In most of these, the hederagenin diol group is protected as an acetonide. However, esters, unmodified diols and other acetal modifications are also part of this set.

Thirteen compounds from the initial library of 36 compounds had been modified with a 3,23-acetal and a 28-ureate (Table S2, group 7). Five compounds (MC023, MC035, MC042, MC062 and MC024) from this group were progressed into adult worm (wild strain) assays due to their activity against NEJs. The first four of these were obtained by reaction of the isocyanate MC015 with a simple cyclic amine, pyrrolidine, piperidine, morpholine and N-methylpiperidine, respectively; the final one by trapping with ammonia.

Eight of the thirteen did not show sufficient activity for further study; notably, the large majority of these carried additional rather polar substituents on the ureate, such as the amino-sugars MC028 and MC030, and MC033 with a free acid. MC059, MC028 and MC030 mimic the sugar residue found in many naturally occurring saponins esterified at position 28. However, none reached the level required (i.e., statistically reducing both motility and phenotypic metrics at 10 µM when compared to DMSO controls) to pass the first screen criterion.

The remaining four ureates in this set (Table S2, group 6) comprised two unmodified 3,23-diols and two compounds partially or completely esterified at the 3,23-positions. The diol MC055, monoacetate MC057 and diacetate MC055 were all active against NEJs and were studied further. The free diol MC030, comprising a sugar based ureate, again showed low activity.

MC023, MC055, MC056, and MC057 are of special interest, because the carbamide group is made from pyrrolidine, while the diol group differs (MC023—acetonide, MC055—diol, MC056—diacetate, and MC057—monoacetate). Although all performed equally effectively against NEJs, there were subtle changes in the order of efficacy against wild strain, immature and adult flukes.

2. The isocyanate MC015 is also a precursor for the two carbamates, MC019 and MC022 (Table S2, group 1). Both carry a 3,23-acetonide group and their carbamate modification carries a longer aliphatic chain. Neither MC019 nor MC022 passed the first selection criterion as they were both inactive against NEJs.

3. In three cases (Table S2, group 4), the compounds contained free acids at position 28, but acetals at 3,23. Only one of these, the simplest (MC014), was sufficiently active enough to pass the first assay checkpoint (i.e., NEJ active). The other two examples (IVL-4 and IVL-3) showed lower activity, possibly due to additional polar groups at the acetal position.

4. In two compounds (Table S2, groups 3,5), the carboxylic ester of the hederagenin scaffold had been converted into an ester group. One (IVL-5) was a free 3,23-diol, the other (IVL-6) was protected as a 3,23-acetal. The former molecule was active in the first screen but was removed at the next stage.

5. In four of the compounds studied (Table S2, group 8), the 28-acid had been reduced to an alcohol, and then esterified. Two (MC037 and MC038) had a free diol at 3,23-positions, and two (MC031 and MC032) were protected as an acetal. Rather surprisingly, the simplest example, the acetate ester MC037, was the only one that passed the first checkpoint (i.e., NEJ active). The corresponding hexadecanoate ester showed little activity.

6. Three compounds (IVL-16, IVL-17 and IVL-18, Table S2, group 2) mimic natural saponins and were modified glycosides with free acid groups at the 28-position. These compounds are glycosides linked to the triterpene core through an acetal link in position 3,23, forming an unnatural bond to the sugar part and preventing a possible cleavage by glycosidases. No compound passed the first assay checkpoint.

7. The two tri-acid derivatives (IVL-1 and IVL-2, Table S2, group 9) showed only low activity in initial NEJ screens.

8. The miscellaneous group (Table S2, group 10) contains three compounds (IVL-10, MC015 and MC034). IVL-10 is an oxidation product of hederagenin containing a free aldehyde group, MC015 is a rather reactive isocyanate and MC034 represents a rather reactive methanesulfonate derivative. All three comprise functional groups that would likely be further transformed in vivo or indeed ex vivo. None of the three passed the first screen (i.e., NEJ active). Of the eleven molecules taken forward to the second screen (wild strain adult flukes), eight were ureate derivatives (Table S3). Such compounds are known to be active against leishmanial parasites [15]. The remaining three molecules were much simpler, but more diverse: a hederagenin 3,23-acetonide-28-acid (MC014), a 28-ester 3,23-diol (IVL-5) and a 3,23-diol in which the 28-acid had been reduced to an alcohol (MC056). MC056 and IVL-5 showed limited activity against wild strain adult liver flukes, and were not studied further, along with three of the ureates (MC023, MC024 and MC056). Again, surprisingly, the minimally modified hederagenin MC014 performed very well in the assay against wild strain adult liver flukes. The final screens (immature and mature Italian strain liver flukes) and dose response studies then reduced the set to five compounds and identified the ureate MC042 as the most promising hederagenin derivative.

The sample set is not sufficiently large to deduce a clear structure activity relationship. However, some indicators can be seen. A natural saponin is characterized by a balanced mixture of polar and nonpolar sides; thus, generally, the blocking of the diol side with an acetal, e.g., as an acetonide, does not exclude activity per se. However, an accessible electron donor at the carboxylic acid side of hederagenin seems to be beneficial.

A limited, but direct, comparison can be done with the four pyrrolidyl carbimides MC023, MC055, MC056 and MC057. The diacetate MC057 and the acetonide MC023 were excluded after the first test, while the diol MC055 and the monoacetate MC057 passed the first screen and displayed activity in the second round. In the final round, MC055 was tested against both wild and Italian strains of adult liver fluke and showed very similar activity to MC014, MC035 and MC062, and was only surpassed by MC042. While MC042 (a morpholine bound ureate), MC035 (piperidine) and MC062 (N-methyl piperidine) are structurally very similar, the non-basic oxygen of the morpholine fragment seems to correlate with better activity than the basic amino-groups of the piperidines. It is also worth noting that MC042, with a morpholine ring on the ureate, was the most active of the analogues tested bearing a simple pyrrolidine ring at that position. Other examples of ureates with highly polar substituents as well as other molecules containing highly polar substituents (i.e., modified sugars attached at either diol or acid positions) do not lead to high flukicidal activity.

Ureates such as those that are active in this study are well known pharmacores and are effective against leishmanial parasites [15]. However, it must be recognised that the much simpler acetal derivative of hederagenin (MC014) is almost as effective in these ex vivo screens as the commercial product TCBZ and the ureate MC042. However, unlike the other compounds evaluated, MC014 showed a moderate proliferative effect on the growth of MDBK cells, leading to it being excluded as a potential carcinogen [35]. Given only five compounds of rather similar structure were evaluated in the studies with MDBK cells, it would be premature to draw too many conclusions. While MC014 is the only structure including a free acid rather than an ureate group, it is rather similar in structure to hederagenin, displaying an acetonide instead of a free diol group. Hederagenin is known to cause apoptosis of cancer cells [46]. Thus, cell proliferation caused by MC014 may not be linked to carcinogenesis in this particular case.

An examination of other simple functional changes to hederagenin would be very useful, though the substantial modifications already examined have led to only modest changes in flukicidal efficacy, suggesting more significant changes will be required in order to achieve a step change in activity compared to TCBZ.

## 5. Conclusions

In this series of experiments, we screened 36 synthetic *H. helix* saponin derivatives, structurally based on the hederagenin molecule, against the liver fluke, *F. hepatica*. After our initial round of ex vivo screening on NEJs, we reduced the count to eleven compounds to be tested in adult liver fluke (wild type) which resulted in six molecules for two additional rounds of testing against immature and adult liver fluke of the Italian strain. Overall, the ureate MC042 showed the highest activity, with efficacy against all stages of liver fluke at levels close to those of TCBZ. The EC_50_ of MC042 obtained from immature and adult fluke assays (at 24 h) are estimated at 1.07 μM and 13.02 μM, respectively. Further derivatization of MC042 may generate more selectively potent compounds, which could lead to the creation of urgently-needed, next-generation anthelmintics.

## Figures and Tables

**Table 1:** Summary of anthelmintic activities and selectivity indices of modified saponin molecules.

Compound	Biological Material and Parameters Assessed		EC_50_ (Parasites) and CC_50_ (Cells) Values ^b^ (µM) with 95% Lower and Higher Confidence Intervals (CI) Indicated	Selectivity Indices (SI)
MC014	Adult	Motility	13.46 (7.07; 23.62)	Not determined
	MDBK	Cytotoxicity	Undefined ^c^	
MC035	Adult	Motility	Not determined ^d^	Not determined
	MDBK	Cytotoxicity	79.84 (46.45; 198.9)	
MC042 ^a^	Immature	Motility	1.07 (0.07; 3.04)	44.37
	Adult	Motility	13.02 ^e^	3.64
	MDBK	Cytotoxicity	47.48 (36.41; 65.29)	
MC055	Adult	Motility	Not determined ^d^	Not determined
	MDBK	Cytotoxicity	34.70 (29.93; 40.11)	
MC062	Adult	Motility	Not determined ^d^	Not determined
	MDBK	Cytotoxicity	13.65 (11.59; 16.05)	

^a^ Only MC042 was subjected to dose response titrations against immature flukes. All compounds were assessed against MDBK cells with selectivity indices (SI) calculated at 24 h post exposure. SI was calculated by dividing the CC_50_ by the EC_50_ values. ^b^ values following exposure for 24 h; ^c^ compound caused cell proliferation. Not determined ^d^, the accurate estimation of EC_50_ value in GraphPad software is not possible for this time point without the inclusion of additional higher compound concentrations. ^e^ Lower and higher 95% CI are ‘very wide’ according to GraphPad software.

## Data Availability

Not applicable.

## Supplementary Materials

The following supporting information can be downloaded at: https://www.mdpi.com/article/10.3390/pharmaceutics15071869/s1, Figure S1: Scatter and dot plot showing the effects of 36 hederagenin derivatives on ultrastructure (phenotype) and motility of *Fasciola hepatica* Newly Excysted Juveniles (NEJs). NEJs (n = 25) were treated with hederagenin derivatives (10 µM + 0.1% DMSO) in RPMI 1640 supplemented with 1% *v*/*v* Fetal Calf Serum (FCS) and 1% *v*/*v* antibiotic/antimycotic solution, incubated for 72 h. Parasites incubated in RPMI 1640 + 0.1% DMSO represent the negative controls whereas Triclabendazole (10 µM + 0.1% DMSO) treated worms are the positive controls. The ultrastructure score of 6 represents dead or dissolved appearance and 1 for normal phenotype; in motility, the score of 5 means motionless and normal movement is 1. (a) Ultrastructure (phenotype) of NEJs treated with MC series of compounds. Significance is calculated by Kruskal–Wallis test followed by Dunn’s multiple comparisons test in Prism GraphPad, with an individual fluke score represented by a circle, means of the population represented in black and error bars represented by red (**, *p* < 0.05, ***, *p* < 0.0001). (b) Motility of NEJs treated with MC series of compounds, with an individual fluke score represented by a circle, means of the population represented in black and error bars represented by red (*, *p* < 0.05, **, *p* < 0.001,***, *p* < 0.0001). (c) Ultrastructure (phenotype) of NEJs treated with IVL series of compounds. Significance is calculated by Kruskal–Wallis test followed by Dunn’s multiple comparisons test in Prism GraphPad, with an individual fluke score represented by a circle, means of the population represented in black and error bars represented by red (**, *p* < 0.05, ***, *p* < 0.0001). (d) Motility of NEJs treated with IVL series of compounds. The statistics and values of significance are same as mentioned in (c). Figure S2: Microscopic images showing ultrastructure (phenotype) of *F. hepatica* NEJs treated with MC014, MC035, MC042, MC055 and MC062. NEJs were assayed with TCBZ, MC014, MC035, MC042, MC055 or MC062 (10 µM in 0.1% DMSO) for 72 h. Control NEJs included those cultivated in RPMI 1640 containing 0.1% DMSO. The images were captured using an Olympus CK2 microscope (20× magnification) with a top-mounted Euromex HD ultra-camera. Table S1: Compounds evaluated in this work. Table S2: NEJ motility and ultrastructure (phenotype) scores derived from screening of hederagenin derivatives at 10 µM. Table S3: Effects of selected compounds on motility of wild strain adult flukes at 40 µM over 72 h. Table S4: Effects of selected compounds on motility of immature fluke at 40 µM over 72 h. Table S5: Effects of dose response assay of MC042 on motility of Italian strain immature flukes over 72 h. Table S6: Effects of dose response assay of selected compounds on motility of adult flukes over 72 h. Table S7: Summary of anthelmintic activities and selectivity indices. Methods S1: Synthesis of new compounds. NMR Spectra S1: Proton and carbon NMR spectra of compounds prepared and evaluated in this work.

## References

[1] Roberts L.S., Janovy J., Schmidt G.D. (2000). Foundations of Parasitology.

[2] Rojo-Vazquez F.A., Meana A., Valcarcel F., Martinez-Valladares M. (2012). Update on trematode infections in sheep. Vet. Parasitol..

[3] Alemneh T. (2019). An Introductory to Fasciolosis. Concepts Dairy Vet. Sci..

[4] McNulty S.N., Tort J.F., Rinaldi G., Fischer K., Rosa B.A., Smircich P., Fontenla S., Choi Y.-J., Tyagi R., Hallsworth-Pepin K. (2017). Genomes of *Fasciola hepatica* from the Americas reveal colonization with *Neorickettsia endobacteria* related to the agents of potomac horse and human sennetsu fevers. PLoS Genet..

[5] Piedrafita D., Spithill T.W., Smith R.E., Raadsma H.W. (2010). Improving animal and human health through understanding liver fluke immunology. Parasite Immunol..

[6] Fox N.J., White P.C., McClean C.J., Marion G., Evans A., Hutchings M.R. (2011). Predicting impacts of climate change on *Fasciola hepatica* risk. PLoS ONE.

[7] Winkelhagen A.J.S., Mank T., de Vries P.J., Soetekouw R. (2012). Apparent triclabendazole-resistant human *Fasciola hepatica* infection, the Netherlands. Emerg. Infect. Dis..

[8] Shrestha S., Barratt A., Fox N.J., Ahmadi B.V., Hutchings M.R. (2020). Financial Impacts of Liver Fluke on Livestock Farms Under Climate Change—A Farm Level Assessment. Front. Vet. Sci..

[9] Mazeri S., Rydevik G., Handel I., Bronsvoort B.M.D., Sargison N. (2017). Estimation of the impact of *Fasciola hepatica* infection on time taken for UK beef cattle to reach slaughter weight. Sci. Rep..

[10] Panic G., Duthaler U., Speich B., Keiser J. (2014). Repurposing drugs for the treatment and control of helminth infections. Int. J. Parasitol. Drugs Drug Resist..

[11] Barrera B., Otero J.A., Egido E., Prieto J.G., Seelig A., Álvarez A.I., Merino G. (2012). The anthelmintic triclabendazole and its metabolites inhibit the membrane transporter ABCG2/BCRP. Antimicrob. Agents Chemother..

[12] Brennan G.P., Fairweather I., Trudgett A., Hoey E., McConville M., Meaney M., Robinson M., McFerran N., Ryan L., Lanusse C. (2007). Understanding triclabendazole resistance. Exp. Mol. Pathol..

[13] Gordon D., Zadoks R., Skuce P., Sargison N. (2012). Confirmation of triclabendazole resistance in liver fluke in the UK. Vet. Rec..

[14] Kelley J.M., Elliott T.P., Beddoe T., Anderson G., Skuce P., Spithill T.W. (2016). Current Threat of Triclabendazole Resistance in *Fasciola hepatica*. Trends Parasitol..

[15] Anderson O., Beckett J., Briggs C.C., Natrass L.A., Cranston C.F., Wilkinson E.J., Owen J.H., Mir Williams R., Loukaidis A., Bouillon M.E. (2020). An investigation of the antileishmanial properties of semi-synthetic saponins. RSC Med. Chem..

[16] Braga F.G., Bouzada M.L.M., Fabri R.L., Matos M.d.O., Moreira F.O., Scio E., Coimbra E.S. (2007). Antileishmanial and antifungal activity of plants used in traditional medicine in Brazil. J. Ethnopharmacol..

[17] Edwards J., Brown M., Peak E., Bartholomew B., Nash R.J., Hoffmann K.F. (2015). The diterpenoid 7-keto-sempervirol, derived from *Lycium chinense*, displays anthelmintic activity against both *Schistosoma mansoni* and *Fasciola hepatica*. PLoS Negl. Trop. Dis..

[18] Majester-Savornin B., Elias R., Diaz-Lanza A.M., Balansard G., Gasquet M., Delmas F. (1991). Saponins of the ivy plant, *Hedera helix*, and their Leishmanicidic activity. Planta Med..

[19] Ribeiro T.G., Chávez-Fumagalli M.A., Valadares D.G., Franca J.R., Lage P.S., Duarte M.C., Andrade P.H.R., Martins V.T., Costa L.E., Arruda A.L.A. (2014). Antileishmanial activity and cytotoxicity of Brazilian plants. Exp. Parasitol..

[20] Chakroborty A., Pritchard D., Bouillon M.E., Cervi A., Cookson A., Wild C., Fenn C., Payne J., Holdsworth P., Capner C. (2022). Flukicidal effects of abietane diterpenoid derived analogues against the food borne pathogen *Fasciola hepatica*. Vet. Parasitol..

[21] Crusco A., Bordoni C., Chakroborty A., Whatley K.C.L., Whiteland H., Westwell A.D., Hoffmann K.F. (2018). Design, synthesis and anthelmintic activity of 7-keto-sempervirol analogues. Eur. J. Med. Chem..

[22] Whiteland H.L., Chakroborty A., Forde-Thomas J.E., Crusco A., Cookson A., Hollinshead J., Fenn C.A., Bartholomew B., Holdsworth P.A., Fisher M. (2018). An *Abies procera*-derived tetracyclic triterpene containing a steroid-like nucleus core and a lactone side chain attenuates in vitro survival of both *Fasciola hepatica* and *Schistosoma mansoni*. Int. J. Parasitol. Drugs Drug Resist..

[23] Faizal A., Geelen D. (2013). Saponins and their role in biological processes in plants. Phytochem. Rev..

[24] Lorent J., Le Duff C.S., Quetin-Leclercq J., Mingeot-Leclercq M.-P. (2013). Induction of highly curved structures in relation to membrane permeabilization and budding by the triterpenoid saponins, α- and δ-Hederin. J. Biol. Chem..

[25] Bun S.-S., Elias R., Baghdikian B., Ciccolini J., Ollivier E., Balansard G. (2008). α -hederin potentiates 5-FU antitumor activity in human colon adenocarcinoma cells. Phytother. Res..

[26] Danloy S., Quetin-Leclercq J., Coucke P., De Pauw-Gillet M.C., Elias R., Balansard G., Angenot L., Bassleer R. (1994). Effects of alpha-hederin, a saponin extracted from *Hedera helix*, on cells cultured in vitro. Planta Med..

[27] Hooshyar H., Talari S., Feyzi F. (2014). Therapeutic effect of *Hedera helix* alcoholic extract against Cutaneous Leishmaniasis caused by *Leishmania major* in Balb/C mice. Jundishapur J. Microbiol..

[28] Quetin-Leclercq J., Elias R., Balansard G., Bassleer R., Angenot L. (1992). Cytotoxic Activity of Some Triterpenoid Saponins. Planta Med..

[29] Song J.H., Yeo S.G., Hong E.H., Lee B.R., Kim J.W., Kim J.H., Jeong H.G., Kwon Y.S., Kim H.P., Lee S. (2014). Antiviral activity of hederasaponin B from *Hedera helix* against enterovirus 71 subgenotypes C3 and C4a. Biomol. Ther..

[30] Julien J., Gasquet M., Maillard C., Balansard G., Timon-David P. (1985). Extracts of the Ivy Plant, *Hedera helix*, and their anthelminthic activity on liver flukes. Planta Med..

[31] Rinaldi L., Biggeri A., Musella V., de Waal T., Hertzberg H., Mavrot F., Torgerson P.R., Selemetas N., Coll T., Bosco A. (2015). Sheep and *Fasciola hepatica* in Europe: The GLOWORM experience. Geospat. Health.

[32] Bouillon M.E., Bertocco K., Bischoff L., Buri M., Davies L.R., Wilkinson E.J., Lahmann M. (2020). Synthesis of Anemoclemosides A and B, two saponins isolated from *Anemoclema glaucifolium*. Eur. J. Org. Chem..

[33] Al-dulayymi J.R., Baird M., Bouillon M.E., Duval S., Ramos Morales E., Newbold C.J., Preskett D., Braganka R., Strawson S.W., Wehrli C. (2016). New Bis Esters of Ivy Sapogenins for Ruminants. Patent.

[34] Singh S.K., Tripathi V.J., Singh R.H. (1990). 3β,24-Dihydroxyolean-12-en-28-oic acid, a pentacyclic triterpene acid from *Lantana indica*. Phytochemistry.

[35] Caldwell J.C. (2021). Alterations in cell proliferation, cell death, or nutrient supply. Tumour Site Concordance and Mechanisms of Carcinogenesis.

[36] Ramdani D., Yuniarti E., Jayanegara A., Chaudhry A.S. (2023). Roles of essential oils, polyphenols, and saponins of medicinal plants as natural additives and anthelmintics in ruminant diets: A systematic review. Animals.

[37] Ali N., Shah S.W.A., Shah I., Ahmed G., Ghias M., Khan I. (2011). Cytotoxic and anthelmintic potential of crude saponins isolated from *Achillea Wilhelmsii* C. Koch and Teucrium Stocksianum boiss. BMC Complement. Altern. Med..

[38] Maestrini M., Tava A., Mancini S., Tedesco D., Perrucci S. (2020). In vitro anthelmintic activity of saponins from *Medicago* spp. against sheep gastrointestinal mematodes. Molecules.

[39] Maestrini M., Tava A., Mancini S., Salari F., Perrucci S. (2019). In vitro anthelmintic activity of saponins derived from *Medicago* spp. plants against donkey gastrointestinal nematodes. Vet. Sci..

[40] Zhou S., Dong J., Liu Y., Yang Q., Xu N., Yang Y., Ai X. (2021). Anthelmintic efficacy of natural saponins against *Gyrodactylus kobayashii* in goldfish (*Carassius auratus*) and their 3D-QSAR analysis. Parasitol. Res..

[41] Lorent J., Lins L., Domenech Ò., Quetin-Leclercq J., Brasseur R., Mingeot-Leclercq M.P. (2014). Domain formation and permeabilization induced by the saponin α-hederin and its aglycone hederagenin in a cholesterol-containing bilayer. Langmuir.

[42] Gao Y., He C., Bi W., Wu G., Altman E. (2016). Bioassay guided fractionation identified hederagenin as a major cytotoxic agent from *Cyclocarya paliurus* Leaves. Planta Med..

[43] Zeng J., Huang T., Xue M., Chen J., Feng L., Du R., Feng Y. (2018). Current knowledge and development of hederagenin as a promising medicinal agent: A comprehensive review. RSC Adv..

[44] Upegui Zapata Y.A., Echeverri F., Quiñones W., Torres F., Nacher M., Rivas L.I., Meira C.D.S., Gedamu L., Escobar G., Archbold R. (2020). Mode of action of a formulation containing hydrazones and saponins against *leishmania* spp. Role in mitochondria, proteases and reinfection process. Int. J. Parasitol. Drugs Drug Resist..

[45] Jiang X., Cao Y., Jørgensen L.V.G., Strobel B.W., Hansen H.C.B., Cedergreen N. (2018). Where does the toxicity come from in saponin extract?. Chemosphere.

[46] Liu B.X., Zhou J.Y., Li Y., Zou X., Wu J., Gu J.F., Yuan J.R., Zhao B.J., Feng L., Jia X.B. (2014). Hederagenin from the leaves of ivy (*Hedera helix* L.) induces apoptosis in human LoVo colon cells through the mitochondrial pathway. BMC Complement. Altern. Med..

