# Supplementary material for: Modified Hederagenin Derivatives Demonstrate Ex Vivo Anthelmintic Activity against Fasciola hepatica
Source: Pharmaceutics. 2023 Jul 3;15(7):1869. doi: 10.3390/pharmaceutics15071869 (PMC10385850; doi:10.3390/pharmaceutics15071869)

pharmaceutics

MDPI

Article

Modified Hederagenin Derivatives Demonstrate Ex Vivo Anthelmintic Activity against *Fasciola hepatica*

Anand Chakroborty [1,2], Deiniol R. Pritchard [3], Marc E. Bouillon [4], Anna Cervi [3], Rolf Kraehenbuehl [4], Charlotte Wild [5], Caroline Fenn [5], Peter Holdsworth [5,6], Colin Capner [5], Gilda Padalino [1,7], Josephine E. Forde-Thomas [1], Joseph Payne [5], Brendan G. Smith [8], Maggie Fisher [5], Martina Lahmann [4,9], Mark S. Baird [3,*] and Karl F. Hoffmann [1,*]

1 The Department of Life Sciences (DLS), Aberystwyth University, Aberystwyth SY23 3DA, UK; anand.chakroborty@ttuhsc.edu (A.C.); gip7@aber.ac.uk (G.P.); jef19@aber.ac.uk (J.E.F.-T.)
2 Department of Cell Biology and Biochemistry, Texas Tech University Health Sciences Center, 3601 4th Street, Lubbock, TX 79430, USA
3 Naturiol Bangor Ltd., MSParc, Gaerwen, Anglesey LL60 6AG, UK; deiniol.rhodri@gmail.com (D.R.P.); annacervi@naturiol.uk (A.C.)
4 School of Natural Sciences, Bangor University, Bangor LL57 2UW, UK; marc.bouillon@endotherm.de (M.E.B.); r.kraehenbuehl@bangor.ac.uk (R.K.); lahmann@kth.se (M.L.)
5 Ridgeway Research Limited, Park Farm Buildings, Park Lane, St. Briavels, Gloucestershire GL15 6QX, UK; wild_charlie@hotmail.co.uk (C.W.); cfenn@ridgewayresearch.co.uk (C.F.); peter.paragon60@gmail.com (P.H.); colin.capner@colincapner.plus.com (C.C.); jpayne@ridgewayresearch.co.uk (J.P.); maggie@shernacre.co.uk (M.F.)
6 PAH Consultancy Pty Ltd., 3/27 Gaunson Crescent, Wanniassa, Canberra 2903, Australia
7 School of Pharmacy and Pharmaceutical Sciences, Cardiff University, Redwood Building, King Edward VII Avenue, Cardiff CF10 3NB, UK
8 Bimeda UK, Bryn Cefni Industrial Estate, Unit 2A, Llangefni LL77 7XA, UK; bgs@brendangsmith.com
9 KTH Royal Institute of Technology, Biomedical Engineering and Health Systems, Hälsovägen 11, 141 52 Huddinge, Sweden
* Correspondence: markbaird@naturiol.uk (M.S.B.); krh@aber.ac.uk (K.F.H.)

Citation: Chakroborty, A.; Pritchard, D.R.; Bouillon, M.E.; Cervi, A.; Kraehenbuehl, R.; Wild, C.; Fenn, C.; Holdsworth, P.; Capner, C.; Padalino, G.; et al. Modified hederagenin derivatives demonstrate ex vivo anthelmintic activity against *Fasciola hepatica*. *Pharmaceutics* **2023**, *15*, x. https://doi.org/10.3390/xxxxx

Academic Editor: Maria João Castro Gouveia

Received: 19 May 2023
Revised: 26 June 2023
Accepted: 28 June 2023
Published: 30 June 2023

Copyright: © 2023 by the authors. Licensee MDPI, Basel, Switzerland. This article is an open access article distributed under the terms and conditions of the Creative Commons Attribution (CC BY) license (https://creativecommons.org/licenses/by/4.0/).

Table S1. Compounds evaluated in this work.

**No.**	Compound	**Molecular Structure**	Reference as in manuscript: where there is no reference or additional information noted, the preparation is described in Methods (page 28) and NMR S1 (page 41) of this article
**1**	IVL-1	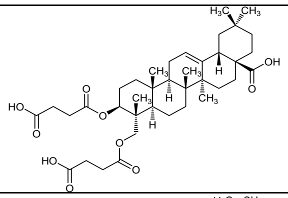	[33]
**2**	IVL-2	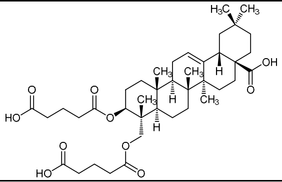	[33]
**3**	IVL-3	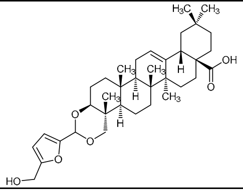	**See Methods S1 and NMR spectra S1**
4	IVL-4	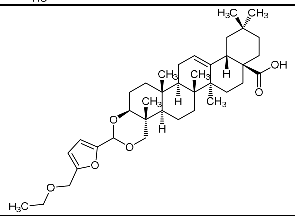	[15]
5	IVL-5	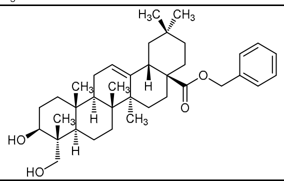	[15]
6	IVL-6	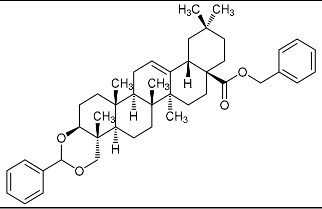	[15]
7	IVL-10	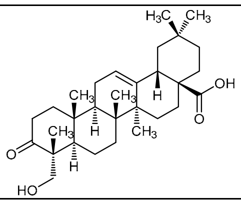	[34]
8	IVL-16	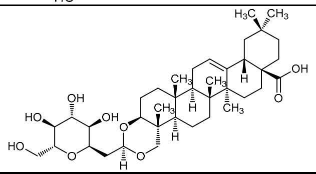	[15] [32]

9	IVL-17	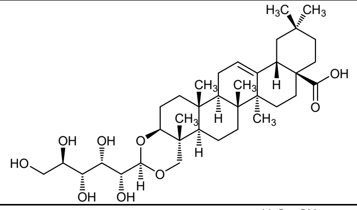	[15]
10	IVL-18	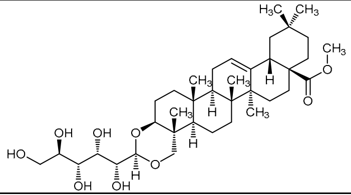	[15]
11	MC014	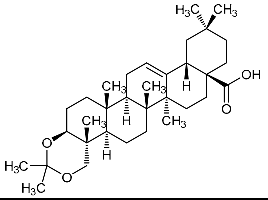	[15]<br>**An additional ^{13}C DEPT-q spectrum is provided in NMR spectra S1**
12	MC015	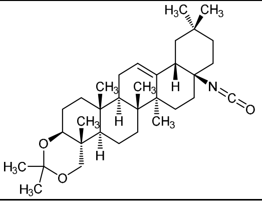	[15]<br>**See Methods S1 and NMR spectra S1**
13	MC017	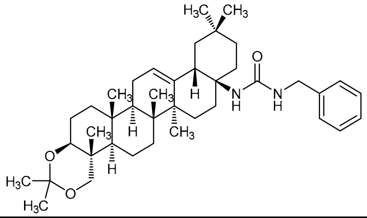	**See Methods S1**
14	MC019	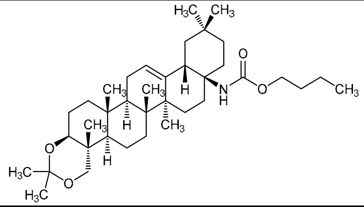	**See Methods S1 and NMR spectra S1**
15	MC022	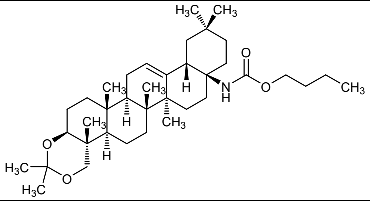	**See Methods S1 and NMR spectra S1**
16	MC023	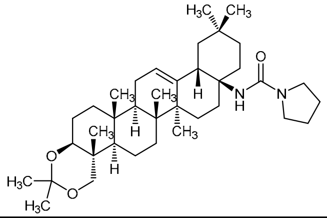	[15]<br>**See Methods S1 and NMR spectra S1**
17	MC024	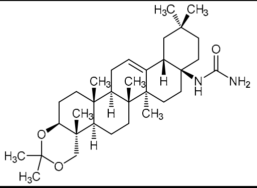	[15]

18	MC027	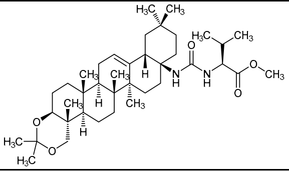	[15]
19	MC028	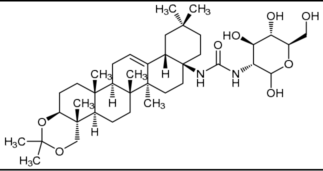	[15]
20	MC030	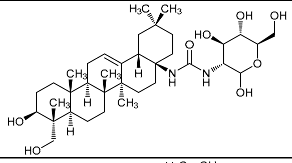	**See Methods S1 and NMR spectra S1**
21	MC031	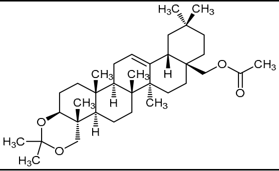	**See Methods S1 and NMR spectra S1**
22	MC032	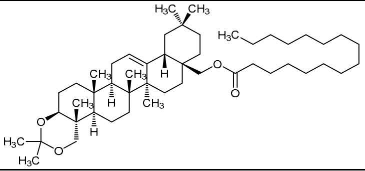	**See Methods S1 and NMR spectra S1**
23	MC033	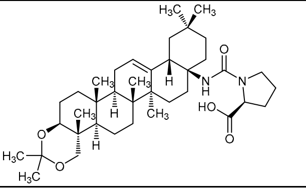	[15]<br>**See Methods S1 and NMR spectra S1**
24	MC034	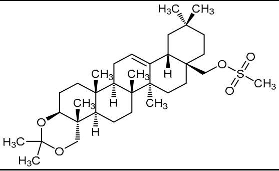	[15]
25	MC035	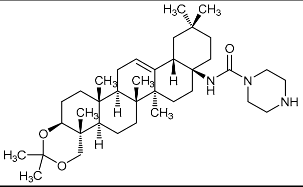	**See Methods S1**
26	MC036	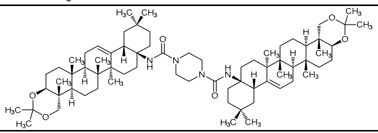	**See Methods S1 and NMR spectra S1**
27	MC037	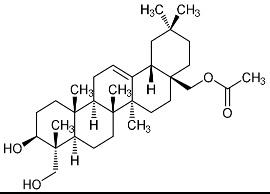	**See Methods S1 and NMR spectra S1**

28	MC038	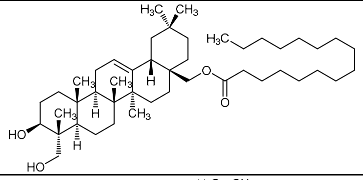	**See Methods S1 and NMR spectra S1**
29	MC042	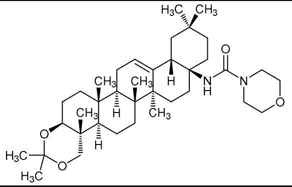	**See Methods S1 and NMR spectra S1**
30	MC044	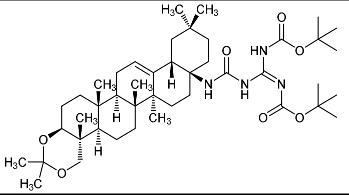	**See Methods S1 and NMR spectra S1**
31	MC048	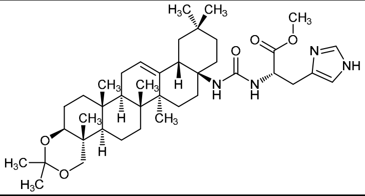	**See Methods S1**
32	MC055	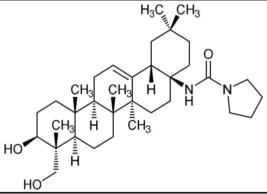	[15]<br>**See Methods S1 and NMR spectra S1**
33	MC056	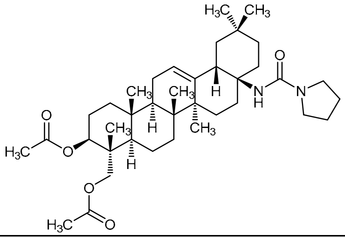	**See Methods S1**
34	MC057	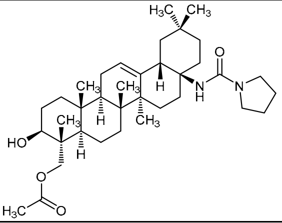	[15]<br>**See Methods S1 and NMR spectra S1**
35	MC059	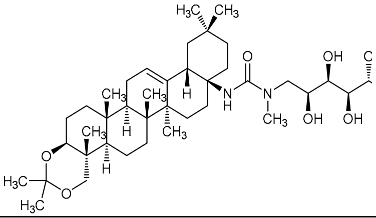	**See Methods S1 and NMR spectra S1**
36	MC062	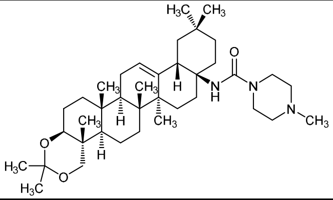	**See Methods S1 and NMR spectra S1**

Table S2. NEJ motility and Ultrastructure (phenotype) scores derived from screening of hederagenin derivatives at 10 µM.

No.	Compounds	**Molecular structure**	Sub-group	**Average phenotype of 25 NEJs with 95% lower and higher confidence intervals (CI) indicated.**			**Significance Adjusted P value***	**Average motility of 25 NEJs with 95% lower and higher confidence intervals (CI) indicated.**			**Significance Adjusted P value***
				**24 hours**	**48 hours**	**72 hours**	**72 hours**	**24 hours**	**48 hours**	**72 hours**	**72 hours**
1	IVL-1	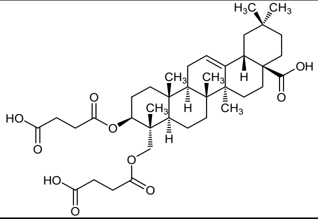	9	1.84 (1.3;2.3)	2.16 (1.6;2.6)	2.72 (2.1;3.3)	**Not Signifi-cant**	1.96 (1.5;2.3)	2.52 (2.1;2.9)	3.24 (2.8;3.6)	**Not Signifi-cant**
2	IVL-2	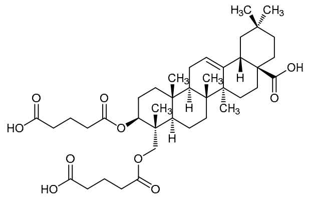	9	2.12 (1.5;2.6)	2.68 (1.9;3.3)	2.88 (2.2;3.5)	**Not Signifi-cant**	2.32 (1.8;2.7)	2.68 (2.1;3.2)	3.68 (3.2;4.1)	**Not Signifi-cant**
3	IVL-3	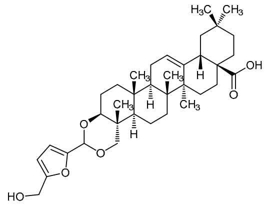	4	3.00 (2.3;3.6)	3.84 (3;4.6)	5.40 (4.9;5.8)	**p<0.05**	3.04 (2.4;3.5)	3.96 (3.5;4.4)	4.56 (4.1;4.9)	**p<0.05**
4	IVL-4	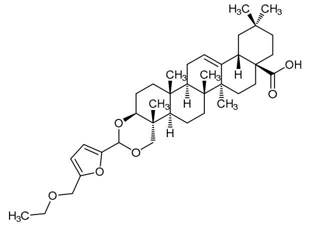	4	2.36 (1.7;3)	3.12 (2.3;3.8)	5.16 (4.6;5.6)	**p<0.05**	2.12 (1.5;2.6)	2.92 2.3;3.5)	4.24 (3.7;4.7)	**p<0.05**

5	IVL-5	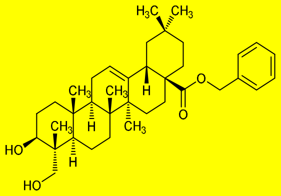	3	5.52 (4.9;6)	5.76 (5.4;6.1)	6 (6.0;6.0)	**p<0.05**	4.6 (4.1;5)	5 (5.0;5.0)	5 (5.0;5.0)	**p<0.05**
6	IVL-6	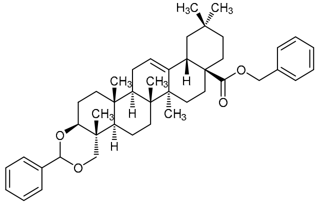	5	1.72 (1.5;1.8)	2 (1.7;2.2)	3.12 (2.9;3.2)	**Not Signifi-cant**	2 (1.7;2.2)	2.36 (2;2.7)	3.12 (2.6;3.6)	**Not Signifi-cant**
7	IVL-10	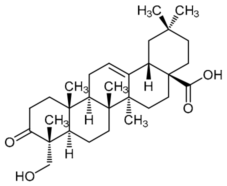	10	3.08 (2.5;3.6)	3.96 (3.4;4.5)	4.92 (4.3;5.4)	**p<0.05**	3.56 (3;4)	4.24 (3.8;4.5)	4.44 (4;4.8)	**p<0.05**
8	IVL-16	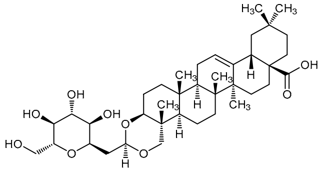	2	1.84 (1.4;2.2)	2.36 (1.7;2.9)	2.64 (2;3.2)	**Not Signifi-cant**	2.12 (1.7;2.4)	2.64 (2.1;3.1)	3.00 (2.4;3.5)	**Not Signifi-cant**
9	IVL-17	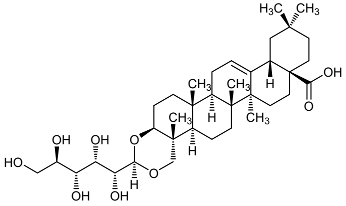	2	1.80 (1.6;1.9)	2.20 (1.6;2.7)	2.56 (2;3)	**Not Signifi-cant**	2.16 (1.8;2.4)	2.88 (2.3;3.4)	2.76 (2.2;3.2)	**Not Signifi-cant**

10	IVL-18	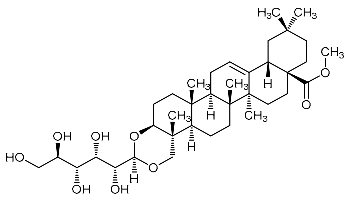	2	2.00 (2;2)	2.32 (2.1;2.5)	3.88 (3.3;4.4)	**$p<0.05$**	4.80 (4.6;4.9)	4.84 (4.6;4.9)	5.00 (5.5)	**$p<0.05$**
11	MC014	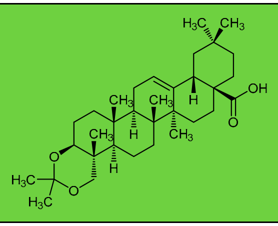	4	4.12 (3.5;4.6)	6.00 (6.0;6.0)	6.00 (6.0;6.0)	**$p<0.05$**	5.00 (5.0;5.0)	5.00 (5.0;5.0)	5.00 (5.0;5.0)	**$p<0.05$**
12	MC015	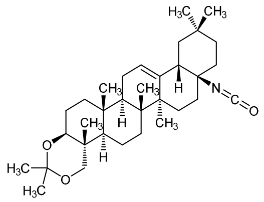	10	2.24 (1.6;2.8)	2.84 (2;3.5)	3.56 (2.7;4.3)	**Not Signifi-cant**	2.56 (2;3)	3.08 (2.4;3.6)	3.12 (2.4;3.7)	**Not Signifi-cant**
13	MC017	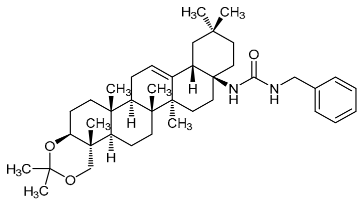	7	3.28 (2.5;4)	4.04 (3.3;4.6)	4.12 (3.4;4.7)	**$p<0.05$**	4.12 (3.7;4.5)	4.0 (3.5; 4.4)	3.92 (3.3; 4.4)	**$p<0.05$**
14	MC019	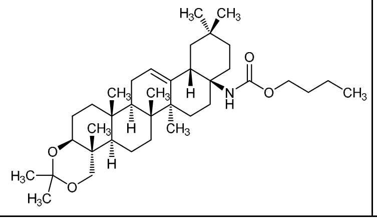	1	2.40 (1.8;2.9)	3.20 (2.6;3.7)	3.40 (2.7;4)	**Not Signifi-cant**	2.24 (1.7;2.7)	4.00 (3.7;4.2)	4.36 (3.9;4.7)	**$p<0.05$**

15	MC022	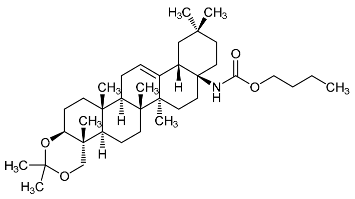	1	4.60 (4.2;4.9)	5.08 (4.7;5.4)	5.88 (5.6;6.1)	**p<0.05**	2.68 (1.9;3.4)	4.12 (3.7;4.4)	4.96 (4.8;5)	**p<0.05**
16	MC023	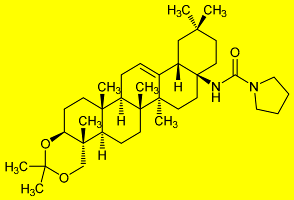	7	**5.00 (5.0;5.0)**	**5.00 (5.0;5.0)**	**5.00 (5.0;5.0)**	**p<0.05**	**5.00 (5.0;5.0)**	**5.00 (5.0;5.0)**	**5.00 (5.0;5.0)**	**p<0.05**
17	MC024	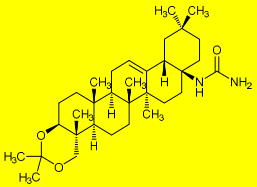	7	**5.00 (5.0;5.0)**	**5.00 (5.0;5.0)**	**5.00 (5.0;5.0)**	**p<0.05**	**5.00 (5.0;5.0)**	**5.00 (5.0;5.0)**	**5.00 (5.0;5.0)**	**p<0.05**
18	MC027	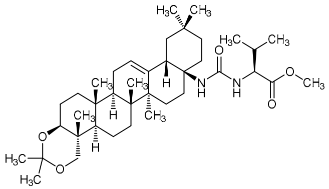	7	3.32 (2.5;4.1)	4.64 (3.9;5.3)	5.48 (4.9;5.9)	**p<0.05**	3.48 (2.9;3.9)	4.72 (4.4;4.9)	4.76 (4.4;5)	**p<0.05**
19	MC028	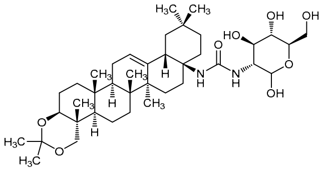	7	2.24 (1.6;2.8)	2.76 (2;3.4)	2.72 (2;3.4)	**Not Significant**	2.32 (1.8;2.7)	3.52 (2.9;4)	2.60 (1.9;3.2)	**Not Significant**

20	MC030	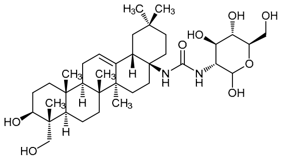	6	3.00 (2.2;3.7)	3.60 (2.9;4.2)	4.00 (3.2;4.7)	**p<0.05**	3.20 (2.6;3.7)	3.92 (3.5;4.2)	3.96 (3.4;4.4)	**p<0.05**
21	MC031	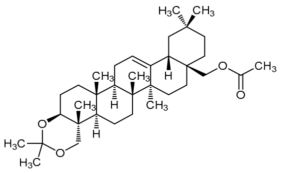	8	2.72 (1.9;3.4)	3.64 (2.9;4.3)	4.80 (4.2;5.3)	**p<0.05**	2.96 (2.4;3.4)	2.76 (2;3.4)	3.44 (2.6;4.2)	**Not Significant**
22	MC032	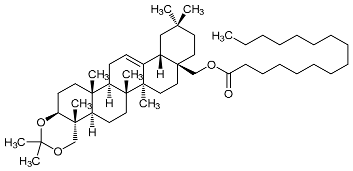	8	2.52 (1.7;3.3)	2.64 (1.8;3.4)	2.80 (2;3.5)	**Not Significant**	3.20 (2.6;3.7)	3.12 (2.5;3.6)	3.12 (2.5;3.7)	**Not Significant**
23	MC033	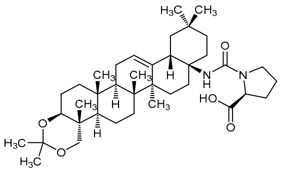	7	3.28 (2.7;3.8)	3.44 (2.8;4)	4.04 (3.3;4.6)	**p<0.05**	3.80 (3.4;4.1)	3.88 (3.5;4.2)	3.76 (3.2;4.2)	**Not Significant**
24	MC034	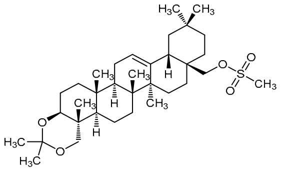	10	2.64 (2.0;3.2)	4.56 (4.2;4.9)	4.56 (4.2;4.9)	**p<0.05**	5.00 (5.0;5.0)	5.00 (5.0;5.0)	**5.00 (5.0;5.0)**	**p<0.05**
25	MC035	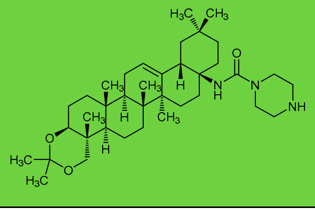	7	**6.00 (6.0;6.0)**	**6.00 (6.0;6.0)**	**6.00 (6.0;6.0)**	**p<0.05**	**5.00 (5.0;5.0)**	**5.00 (5.0;5.0)**	**5.00 (5.5)**	**p<0.05**

26	MC036	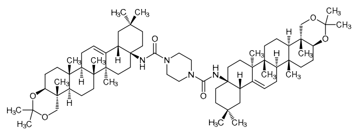	7	1.80 (1.6;1.9)	3.04 (2.2;3.7)	3.16 (2.4;3.8)	**Not Signifi-cant**	2.64 (2.3;2.9)	2.80 (2.1;3.4)	3.60 (3;4.1)	**Not Signifi-cant**
27	MC037	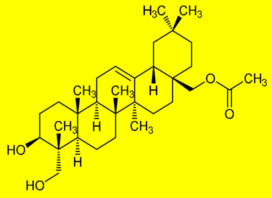	8	6.00 (6.0;6.0)	6.00 (6.0;6.0)	6.00 (6.0;6.0)	**p<0.05**	5.00 (5.0;5.0)	5.00 (5.0;5.0)	5.00 (5.0;5.0)	**p<0.05**
28	MC038	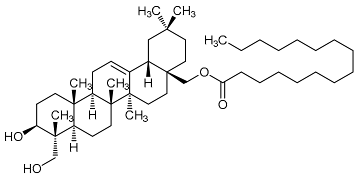	8	2.32 (1.7;2.9)	2.92 (2.1;3.6)	3.48 (2.7;4.2)	**Not Signifi-cant**	2.96 (2.3;3.5)	2.80 (2.2;3.3)	3.32 (2.7;3.8)	**Not Signifi-cant**
29	MC042	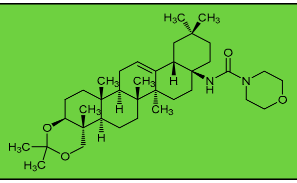	7	**6.00 (6.0;6.0)**	**6.00** (6.0;6.0)	**6.00** (6.0;6.0)	**p<0.05**	**5.00** (5.0;5.0)	**5.00** (5.0;5.0)	**5.00** (5.0;5.0)	**p<0.05**
30	MC044	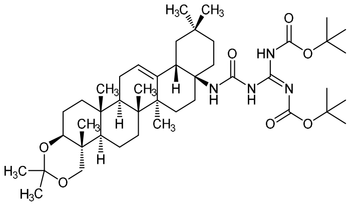	7	1.92 (1.7;2)	2.04 (1.8;2.2)	2.48 (1.9;3)	**Not Signifi-cant**	2.64 (2.3;2.8)	2.64 (2.3;2.9)	2.76 (2.3;3.1)	**Not Signifi-cant**

31	MC048	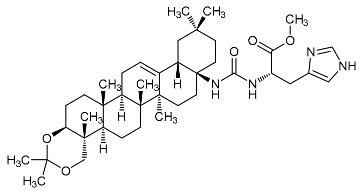	7	1.44 (1.2;1.6)	**X**	1.88 (1.7;2)	**Not Significant**	1.40 (1.2;1.5)	**X**	1.96 (1.8;2)	**Not Significant**
32	MC055	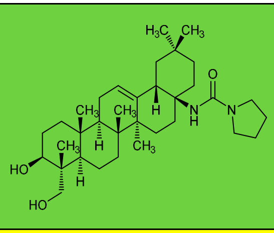	6	**6.00 (6.0;6.0)**	**6.00 (6.0;6.0)**	**6.00 (6.0;6.0)**	**p<0.05**	**5.00 (5.0;5.0)**	**5.00 (5.0;5.0)**	**5.00 (5.0;5.0)**	**p<0.05**
33	MC056	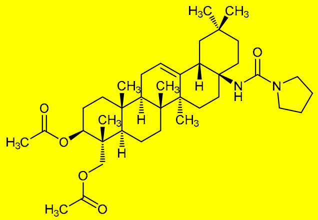	6	**6.00 (6.0;6.0)**	**6.00 (6.0;6.0)**	**6.00 (6.0;6.0)**	**p<0.05**	**5.00 (5.0;5.0)**	**5.00 (5.0;5.0)**	**5.00 (5.0;5.0)**	**p<0.05**
34	MC057	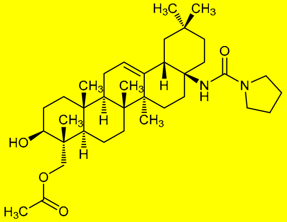	7	**6.00 (6.0;6.0)**	**6.00 (6.0;6.0)**	**6.00 (6.0;6.0)**	**p<0.05**	**5.00 (5.0;5.0)**	**5.00 (5.0;5.0)**	**5.00 (5.0;5.0)**	**p<0.05**
35	MC059	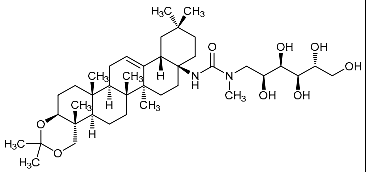	7	2.68 (1.9;3.3)	3.04 (2.3;3.7)	3.88 (3.2;4.4)	**p<0.05**	2.84 (2.3;3.3)	3.20 (2.7;3.6)	3.20 (2.6;3.7)	**Not Significant**

36	MC062	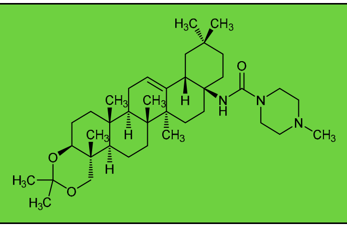	7	**6.00 (6.0;6.0)**	**6.00 (6.0;6.0)**	**6.00 (6.0;6.0)**	**$p<0.05$**	**5.00 (6.0;6.0)**	**5.00 (6.0;6.0)**	**5.00 (6.0;6.0)**	**$p<0.05$**
**DMSO (0.1%) in RPMI 1640****				1.56 (0.8; 1.1)	1.86 (0.7; 1.2)	1.94 (0.8; 1.1)	**Not Significant**	1.76 (0.7;1.2)	2.18 (0.7;1.2)	2.86 (0.7;1.2)	**Not Significant**
**Triclabendazole (40μM)****				6.00 (6.0;6.0)	6.00 (6.0;6.0)	6.00 (6.0;6.0)	**$p<0.05$**	5.00 (5.0;5.0)	5.00 (5.0;5.0)	5.00 (5.0;5.0)	**$p<0.05$**

Significance is relative to parasites cultivated in RPMI 1640 containing 0.1% DMSO and *collected at the 72 hour time point for each concentration, ** the average phenotype and motility scores were obtained from 50 NEJs. The screening of IVL and MC series were conducted separately.

Compounds marked in green or yellow are those selected for further examination in other assays; those in green were progressed to the final assay.

X—data not available. In this Table, the compounds in the library are also subdivided according to their functionality into subgroups:

1. Hederagenin 28-carbamates
2. Anemoclemosides.
3. Hederagenin 28-esters
4. Hederagenin 3,23-acetals
5. Hederagenin 3,23-protected 28-esters
6. Hederagenin 28-ureas
7. Hederagenin 3,23-protected 28-ureas
8. Hederagenin 28-methanol derivatives
9. Hederagenin esters of diacids
10. Other

pharmaceutics

MDPI

Table S3. Effects of selected compounds on motility of wild strain adult flukes at 40 μM over 72 hours.

Compounds	Molecular structure	Short-hand structure	Average motility of 4 parasites with 95% lower and higher confidence intervals (CI) at 72 hour time point indicated.	Significance Adjusted P value*
MC014	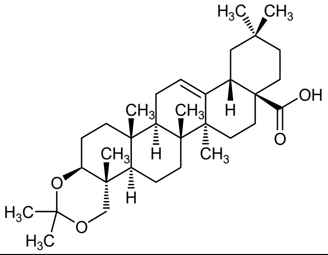		6.00 (6.0;6.0)	**p<0.05**
MC023			4.75 (3.5;5.9)	**p<0.05**
MC024			4.25 (3.2;5.7)	**p<0.05**
MC035			5.75 (5.2; 6.2)	**p<0.05**
MC037	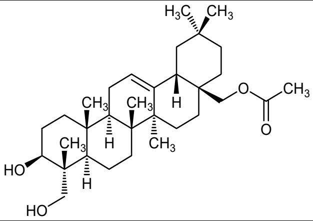		3.25 (2;4.4)	**Not Significant**
MC042			6.00 (6.0;6.0)	**P<0.05**

MC055	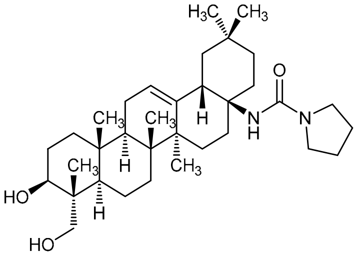	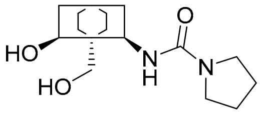 MC055	5.25 (4.7;5.7)	**p<0.05**
MC056	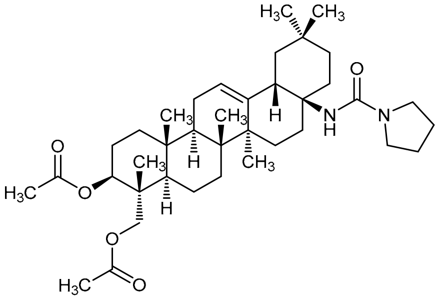	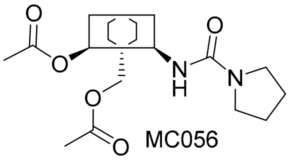 MC056	4.25 (3.3;5.1)	**Not Significant**
MC057	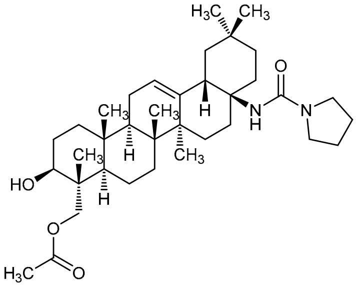	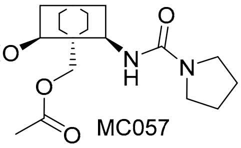 MC057	5.00 (5.0;5.0)	**p<0.05**
MC062	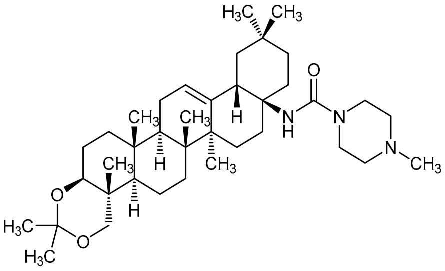	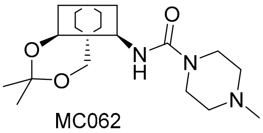 MC062	5.00 (5.0;5.0)	**p<0.05**
IVL-5	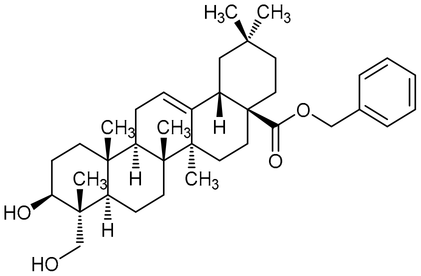	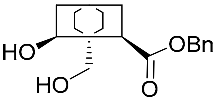 IVL-5	2.75 (2.2;3.2)	**Not Significant**
	**DMSO (0.4%) in RPMI 1640**		1.00 (1.0; 1.0)	**Not Significant**
	**Triclabendazole**		6.00 (6.0;6.0)	**p<0.05**

These compounds are those selected to screen on adult fluke based on their activity against NEJs. The full structure and a short-hand version showing the fixed triterpene skeleton and the variable functional groups are both presented. Significance is relative to parasites cultivated in RPMI 1640 containing 0.4% DMSO and *collected at the 72 hour time point for each concentration.

pharmaceutics MDPI

Table S4. Effects of selected compounds on motility of immature flukes at 40 µM over 72 hours.

Compound	Molecular structure	Short-hand structures	Average motility of 4 parasites with 95% lower and higher confidence intervals (CI) at 24 hour time point indicated.	Average motility of 4 parasites with 95% lower and higher confidence intervals (CI) at 48 hour time point indicated.	Average motility of 4 parasites with 95% lower and higher confidence intervals (CI) at 72 hour time point indicated.	Significance Adjusted P value*
MC014	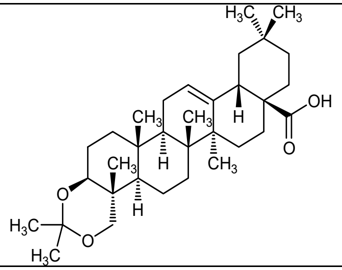		5.00 (5.0;5.0)	5.00 (5.0;5.0)	5.00 (5.0;5.0)	**p<0.05**
MC035			5.00 (5.0;5.0)	5.00 (5.0;5.0)	5.00 (5.0;5.0)	**p<0.05**
MC042			4.75 (4.2;5.2)	5.00 (5.0;5.0)	5.00 (5.0;5.0)	**p<0.05**

MC055	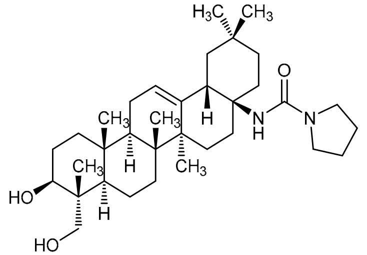	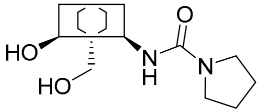 MC055	2.75 (1.8;3.6)	5.00 (5.0;5.0)	5.00 (5.0;5.0)	**p<0.05**
MC057	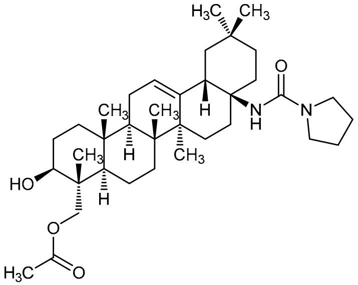	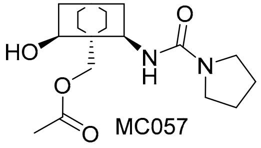 MC057	3.50 (2.5;4.4)	4.25 (3.3;5.1)	4.50 (3.5;5.4)	**0.182**
MC062	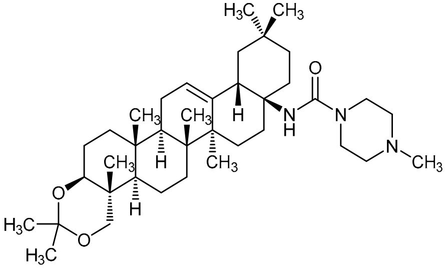	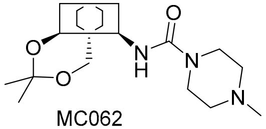 MC062	5.00 (5.0;5.0)	5.00 (5.0;5.0)	5.00 (5.0;5.0)	**<0.05**
**DMSO (0.4%) in RPMI 1640**			1.00 (1.0,1.0)	1.00 (1.0,1.0)	1.00 (1.0,1.0)	**Not Significant**
**Triclabendazole**			5.0 (5.0,5.0)	5.0 (5.0,5.0)	5.0 (5.0,5.0)	**<0.05**

These compounds are those selected for evaluation on the basis of their effects on wild strain adult flukes. Significance is relative to parasites cultivated in RPMI 1640 containing 0.4% DMSO and *collected at the 72 hour time point for each concentration.

Table S5. Effects of dose response assay of MC042 on motility of Italian strain immature flukes over 72 hours.

Compounds	**Molecular Structure**	Short-hand structures	**Average motility of 2 parasites treated with 4.4 µM; with 95% lower and higher confidence intervals (CI) at three time points indicated.**			**Average motility of 2 parasites treated with 13.3 µM; with 95% lower and higher confidence intervals (CI) at three time points indicated.**			**Average motility of 2 parasites treated with 40 µM; with 95% lower and higher confidence intervals (CI) at three time points indicated.**			**Significance Adjusted P value*<br><br>(for 40 µM treated worms for 72 hours)**
			**24 hours**	**48 hours**	**72 hours**	**24 hours**	**48 hours**	**72 hours**	**24 hours**	**48 hours**	**72 hours**	
MC042	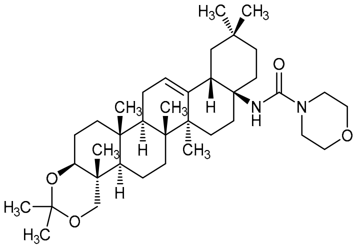	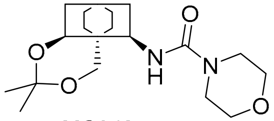 MC042	4.00 (4.0;4.0)	5.00 (5.0; 5.0)	5.00 (5.0; 5.0)	4.00 (4.0;4.0)	5.00 (5.0; 5.0)	5.00 (5.0; 5.0)	5.00 (5.0;5.0)	5.00 (5.0;5.0)	5.00 (5.0,5.0)	**<0.05**
**Triclabendazole**									5.00 (5.0; 5.0)	5.00 (5.0; 5.0)	5.00 (5.0; 5.0)	**<0.05**
**DMSO (0.4%) in RPMI 1640**									1.00 (1.0,1.0)	1.00 (1.0,1.0)	1.00 (1.0,1.0)	**Not Significant**

Significance is relative to parasites cultivated in RPMI 1640 containing 0.4% DMSO and *collected at the 72 hour time point for each concentration.

Table S6. Effects of dose response assay of selected compounds on motility of adult flukes over 72 hours.

Compounds	**Molecular Structure**	Short-hand structures	**Average motility of 3 parasites treated with 4.4 μM; with 95% lower and higher confidence intervals (CI) at three time points indicated.**			**Average motility of 3 parasites treated with 13.3 μM; with 95% lower and higher confidence intervals (CI) at three time points indicated.**			**Average motility of 3 parasites treated with 40 μM; with 95% lower and higher confidence intervals (CI) at three time points indicated.**			**Significance adjusted P value* (for 40 μM treated worms at 72 hours)**
			**24 hours**	**48 hours**	**72 hours**	**24 hours**	**48 hours**	**72 hours**	**24 hours**	**48 hours**	**72 hours**	
MC014	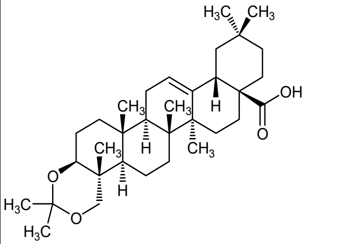	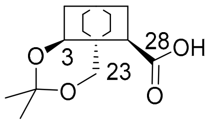 MC014	1.00 (1.0;1.0)	1.00 (1.0;1.0)	3.00 (1.8; 4.1)	1.33 (0.6;1.9)	1.66 (0.3;2.9)	3.33 (2.6;3.9)	3.33 (2.6;3.9)	4.00 (4.0;4.0)	5.33 (4.0;6.6)	**<0.05**
MC035	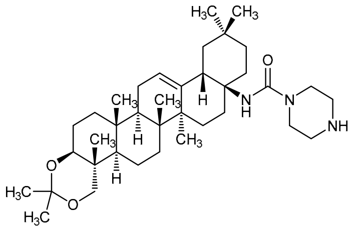	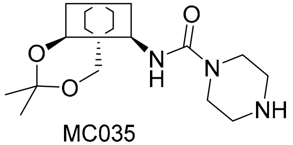 MC035	1.00 (1.0;1.0)	1.00 (1.0;1.0)	1.00 (1.0;1.0)	1.00 (1.0;1.0)	1.00 (1.0;1.0)	3.66 (2.3; 4.9)	2.33 (1.6;2.9)	4.33 (3.6;4.9)	5.00 (3.8;6.1)	**<0.05**
MC042	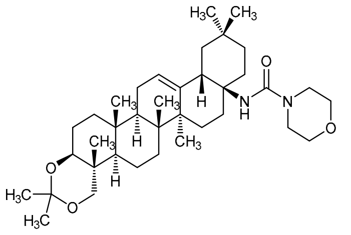	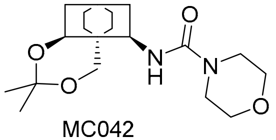 MC042	1.00 (1.0;1.0)	2.00 (2.0; 2.0)	3.00 (3.0; 3.0)	3.66 (3.0;4.3)	4.00(4.0; 4.0)	4.00 (4.0; 4.0)	5.33 (4.6;5.9)	5.66 (5.0;6.3)	5.66 (5.0,6.3)	**<0.05**

MC055	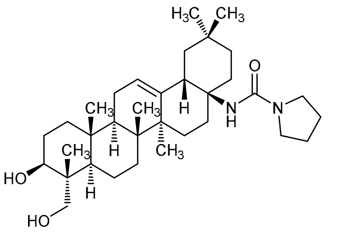	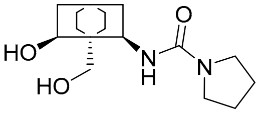 MC055	1.00 (1.0;1.0)	1.00 (1.0;1.0)	3.00 (3.0;3.0)	1.00 (1.0;1.0)	1.00 (1.0;1.0)	4.66 (3.3;5.9)	3.00 (3.0;3.0)	3.66 (3.0;4.3)	5.00 (5.0;5.0)	**<0.05**
MC062	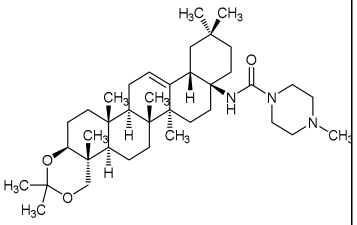	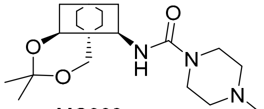 MC062	1.00 (1.0; 1.0)	1.00 (1.0; 1.0)	3.00 (1.0; 1.0)	1.00 (1.0; 1.0)	1.66 (1.0; 2.3)	5.00 (5.0; 5.0)	3.00 (3.0;3.0)	4.00 (4.0;4.0)	5.00 (5.0;5.0)	**<0.05**
**Triclabendazole**			1.00 (1.0; 1.0)	1.00 (1.0; 1.0)	1.00 (1.0; 1.0)	1.00 (1.0;1.0)	1.33 (0.6;1.9)	2.00 (0.8; 3.1)	2.33 (1.6; 2.9)	5.66 (5.0; 6.3)	6.00 (6.0; 6.0)	**<0.05**
**DMSO (0.4%) in RPMI 1640**									1.00 (1.0,1.0)	1.00 (1.0,1.0)	1.00 (1.0,1.0)	**Not Significant**

Significance is relative to parasites cultivated in RPMI 1640 containing in 0.4% DMSO and *collected at the 72 hour time point for each concentration.

Table S7. Summary of anthelmintic activities and selectivity indices.

Compounds	EC_{50} with 95% lower and higher confidence intervals (CI) on Adult Flukes (µM)		EC_{50} with 95% lower and higher confidence intervals (CI) on Immature Flukes (µM)	CC_{50} with 95% lower and higher confidence intervals (CI) on MDBK cells (µM)	Selectivity Index (SI) on Adult Flukes SI=CC_{50}/EC_{50}		Selectivity Index (SI) on Immature Flukes SI=CC_{50}/EC_{50}
	24 hour	72 hour	24 hour	24 hour	24 hour	72 hour	24 hour
MC014	13.46 (7.07; 23.62)	9.30 (2.83; 17.98)	NA	Undefined	Undefined	Undefined	Undefined
MC035	Not determined*	14.27 (9.87; 21.99)	NA	79.84 (46.45; 198.9)	Undefined	5.59	NA
MC042	13.02±	7.15 (5.27; 9.22)	1.07 (0.07; 3.04)	47.48 (36.41; 65.29)	3.64	6.64	44.37
MC055	Not determined*	6.06 (2.69; 9.36)	NA	34.70 (29.93; 40.11)	Undefined	5.72	NA
MC062	Not determined*	5.61 (3.89; 7.24)	NA	13.65 (11.59; 16.05)	Undefined	2.43	NA

Not determined*—the accurate estimation of EC_{50} value in GraphPad software is not possible for this time point without the inclusion of additional higher compound concentrations.

±—lower and higher 95% CI are very wide.

NA—Not applicable or investigation not conducted.

Figure S1. Scatter and dot plot showing the effects of 36 hederagenin derivatives on Ultrastructure (phenotype) and motility of *Fasciola hepatica* Newly Excysted Juveniles (NEJs).

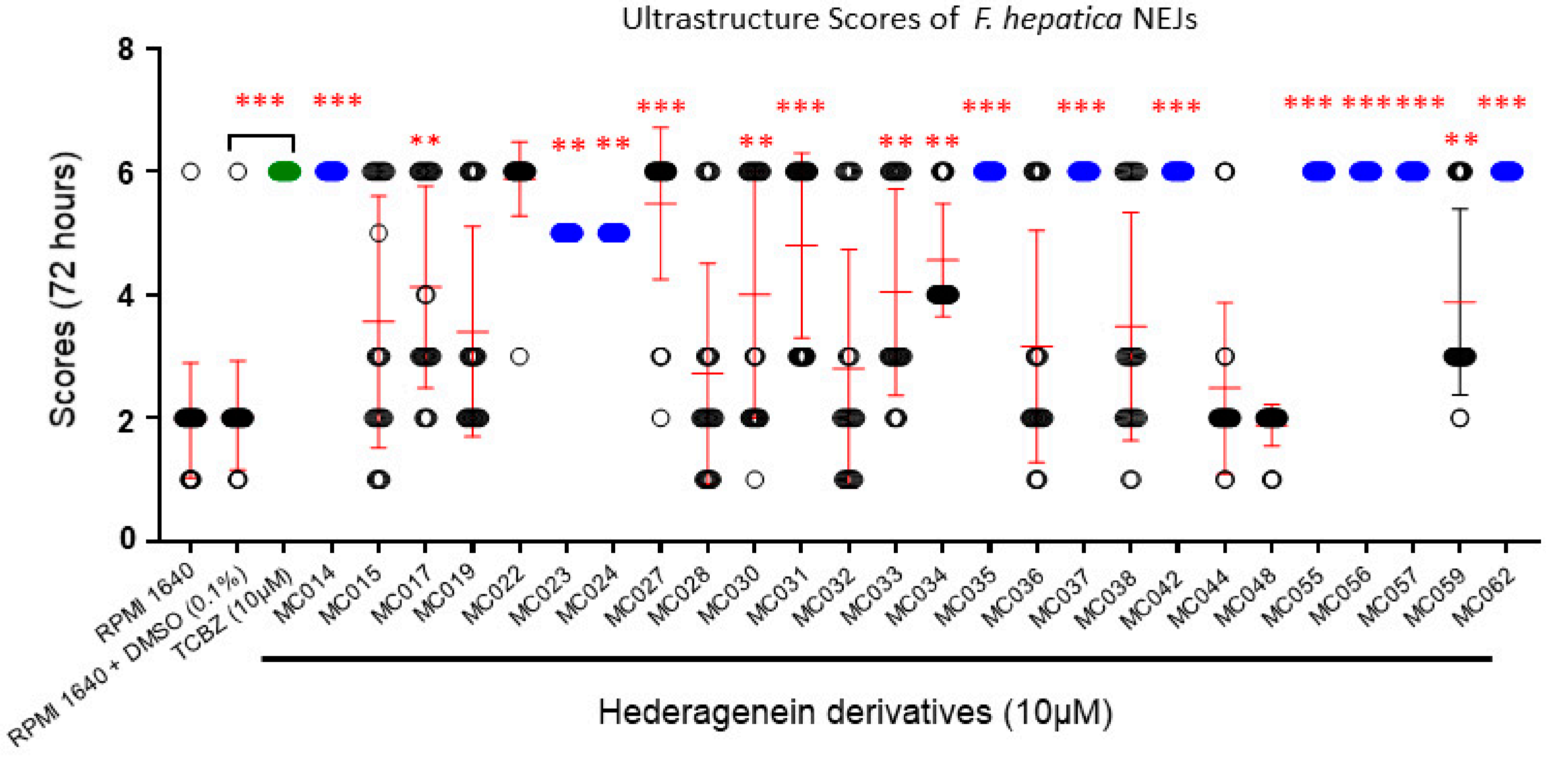

Figure S1(a): *F. hepatica* Newly Excysted Juveniles (NEJs) treated with MC series of compounds.

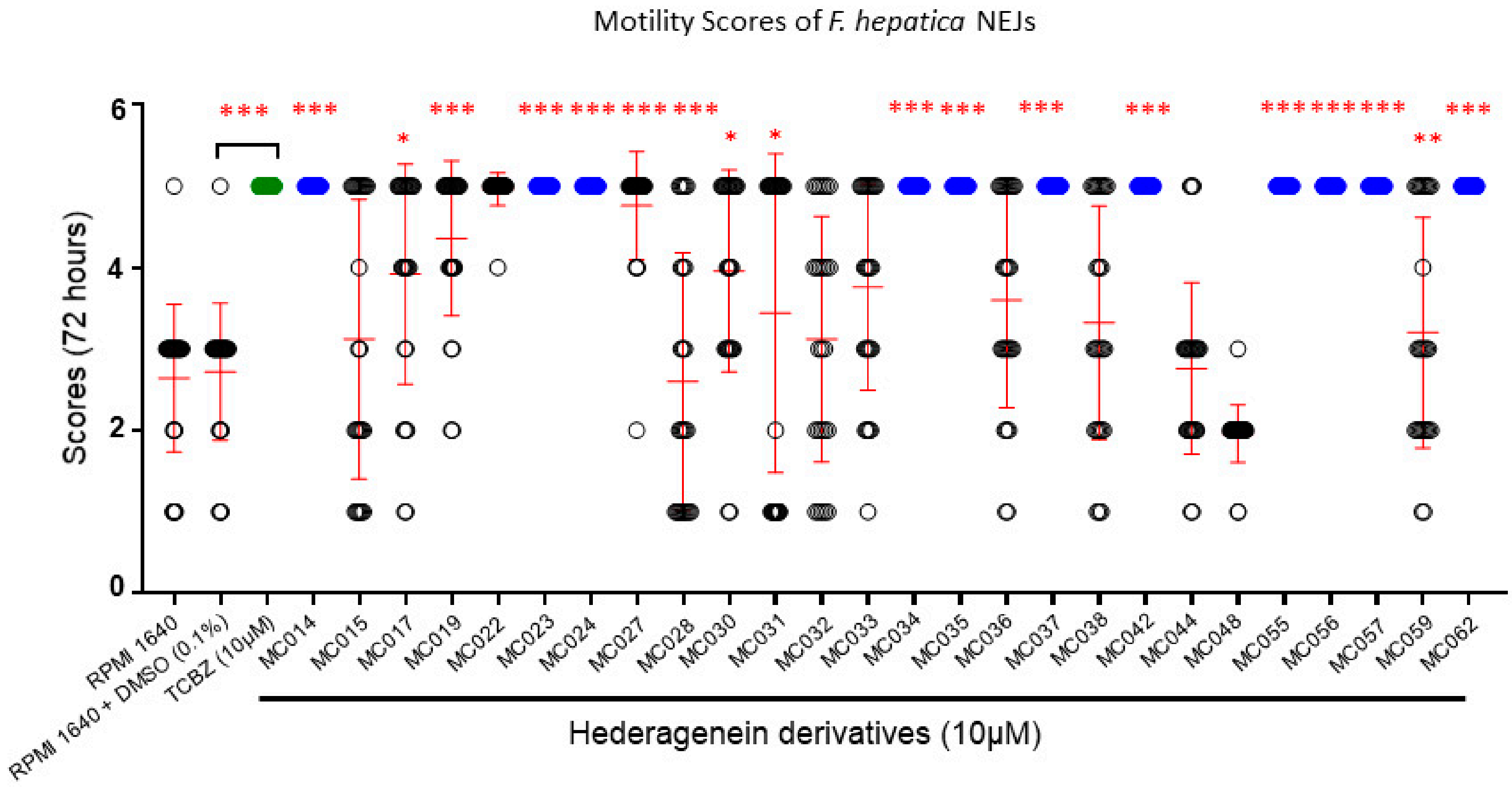

Figure S1(b): *F. hepatica* Newly Excysted Juveniles (NEJs) treated with MC series of compounds.

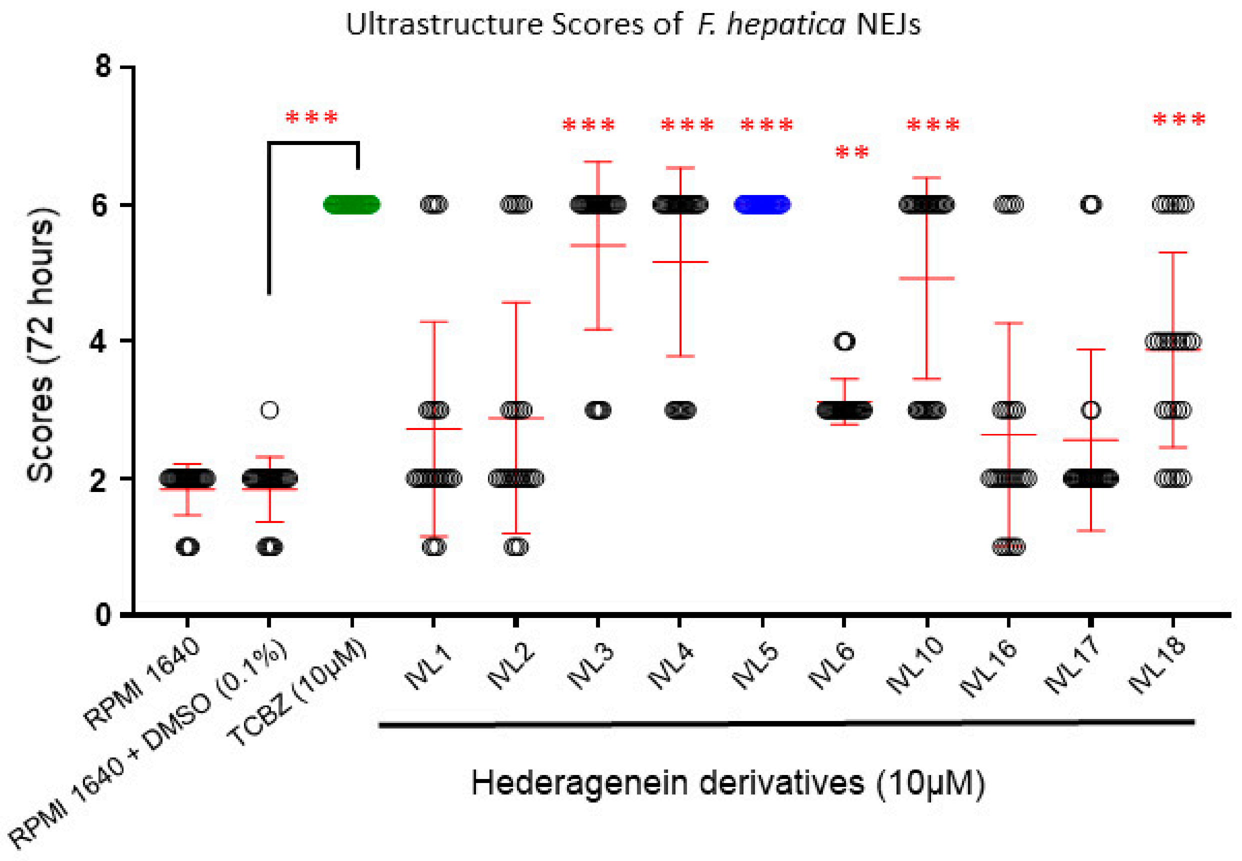

Figure S1(c): *F. hepatica* Newly Excysted Juveniles (NEJs) treated with IVL series of compounds.

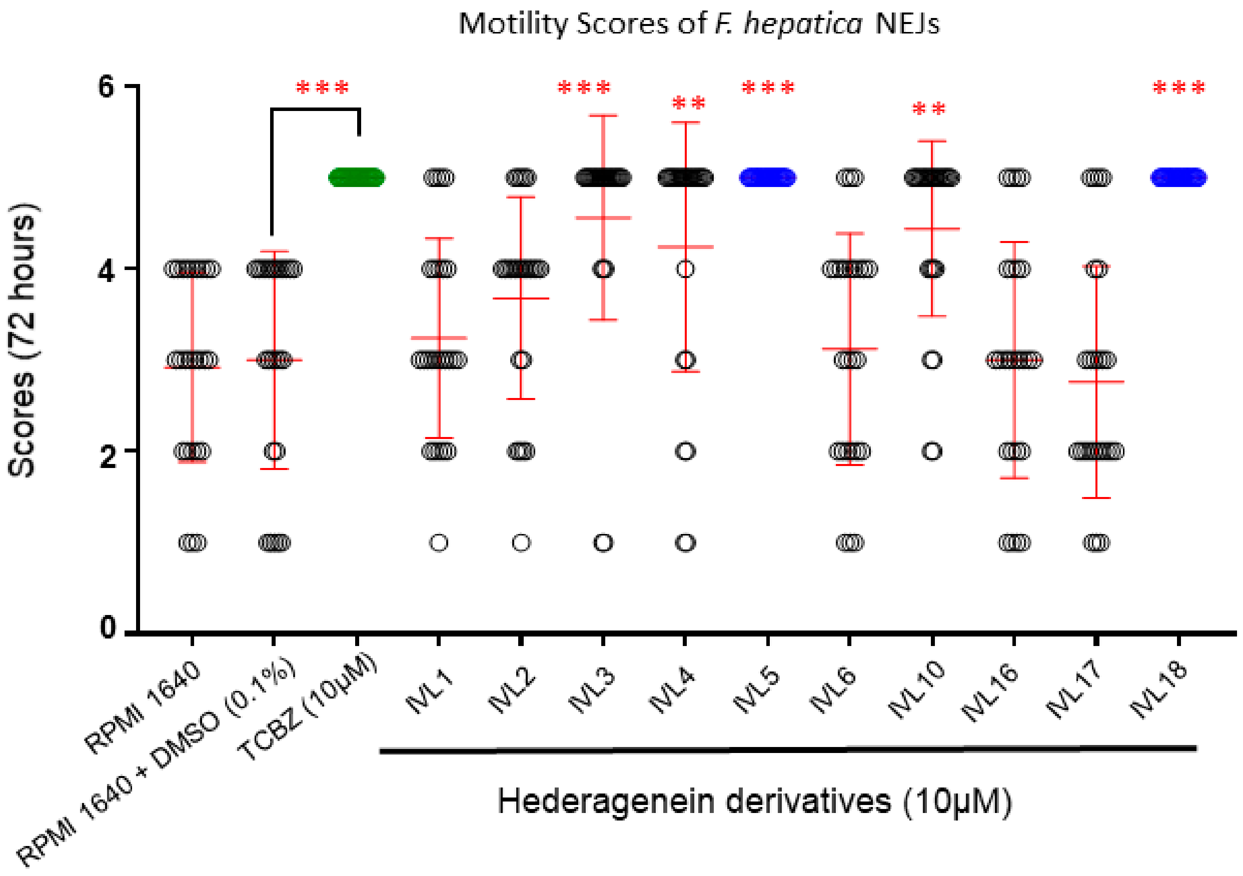

Figure S1(d): *F. hepatica* Newly Excysted Juveniles (NEJs) treated with IVL series of compounds.

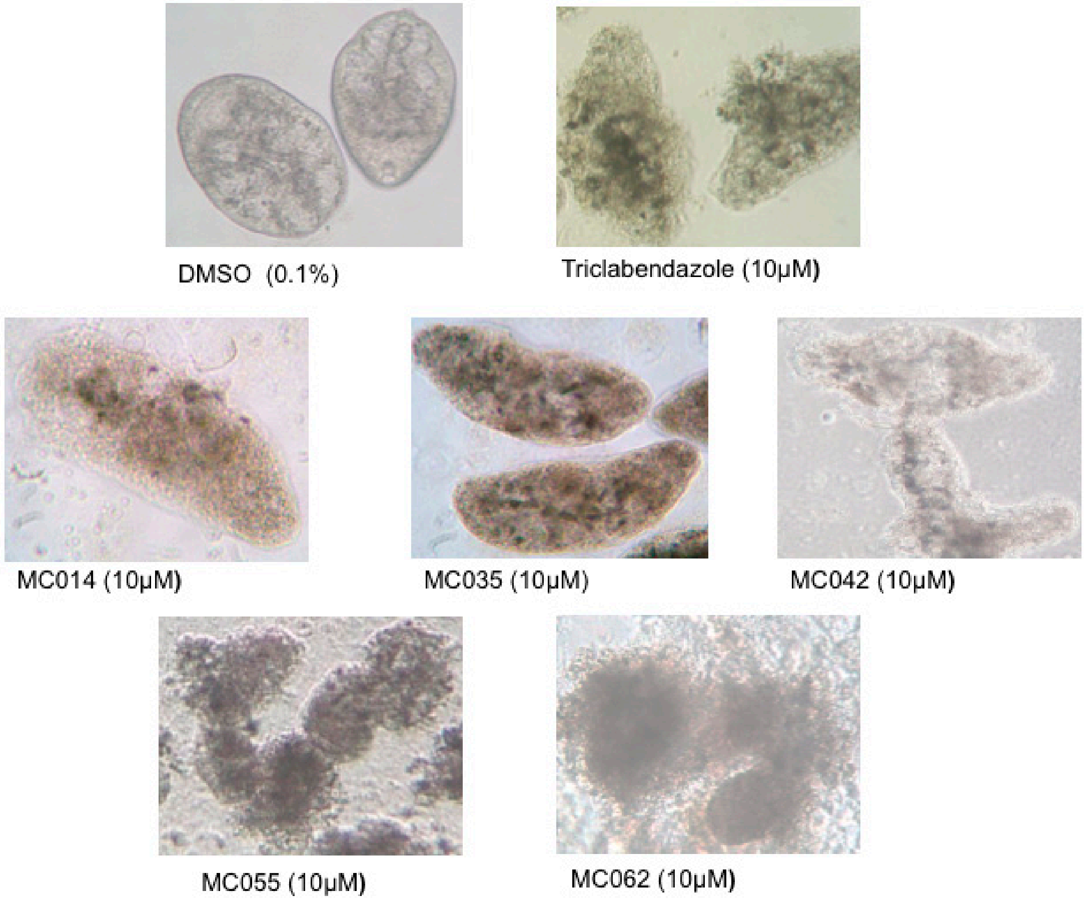

Figure S2. Microscopic images showing ultrastructure (phenotype) of *F. hepatica* NEJs treated with MC014, MC035, MC042, MC055 and MC062**.**

Methods S1 – Synthesis of new compounds

This section includes additional data for some known compounds referenced in **Table S1**.

RT – room temperature; DPPA – diphenylphosphoryl azide; DMF – dimethylformamide; DCM – dichloromethane; petrol – petroleum b.p. 40 – 60 °C.

(4a*R*,6a*R*,6b*S*,8a*S*,14b*R*)-8a-Isocyanato-2,2,4a,6a,6b,11,11,14b-octamethyl-4a,4b,5,6,6a,6b,7,8,8a,9, 10,11,12,12a,14,14a,14b,15,16,16a-eicosahydro-4*H*-piceno[3,4-*d*][1,3]dioxine (MC015**)**

To a solution of MC014 (0.50g, 0.98mmol) and DPPA (0.40g, 0.31mL, 1.47mmol) in dry toluene (25mL), NEt_3 (0.17g, 0.23mL, 1.76mL) was added, and the mixture was then heated to 90°C under a nitrogen atmosphere for 1 hour. The reaction mixture was then adsorbed on silica and the material was applied directly to a silica; column chromatography (10:1 petrol:ethyl acetate; silica NEt_3 neutralized) afforded the product [15],as a white, crystalline solid (0.40g; 80%); R_f (SiO_2; 6:1 petrol:ethyl acetate) = 0.49; ^{1}H NMR (400MHz, benzene-d_6) δ_H : 5.21 (t, *J* = 3.6 Hz, 1H), 3.59 (d, *J* = 10.4 Hz, 1H), 3.43 (dd, *J* = 11.8, 3.8 Hz, 1H), 3.34 (d, *J* = 10.5 Hz, 1H), 2.40 – 2.30 (m, 1H), 2.00 (td, *J* = 13.4, 4.4 Hz, 1H), 1.89 – 1.59 (m, 5H), 1.59 – 0.97 (m, 13H), 1.54 (s, 3H), 1.34 (s, 3H), 1.19 (s, 3H), 1.08, (s, 3H), 1.03 (s, 3H), 0.92 (m, 1H), 0.87 – 0.76 (m, 1H), 0.85 (s, 3H), 0.80 (s, 3H), 0.79 (s, 3H), 0.74 – 0.68 (m, 1H); ^{13}C NMR (101MHz, benzene-d_6) δ_C : 142.8, 124.5, 123.3, 99.0, 77.9, 72.8, 62.0, 52.0, 49.4, 48.1, 47.6, 41.8, 40.1, 39.2, 37.9, 37.5, 37.0, 35.7, 32.8_3, 32.7_5, 30.8, 30.4, 27.3, 27.1, 25.9, 24.1, 23.7_9, 23.7_4, 19.5, 17.9, 17.2, 16.7, 13.0; HMRS [M+H]$^+$: 527.4204. $C_{33}H_{55}O_2N_2$ requires: 527.4207.

1-Benzyl-3-((4a*R*,6a*R*,6b*S*,8a*S*,14b*R*)-2,2,4a,6a,6b,11,11,14b-octamethyl-4a,5,6,6a,6b,7,8, 9,10,11,12,12a,14,14a,14b,15,16,16a-octadecahydro-4*H*-piceno[3,4-*d*][1,3]dioxin-8a(4b*H*)-yl)urea (MC017**)**

A solution of MC015 (0.049 g, 1 mol.eq.) and benzylamine (0.06 mL, 5.7 mol.eq.) in anhydrous toluene (1 mL) was refluxed for 1 hour. The mixture was allowed to cool to RT and was then concentrated under vacuum. The residue was purified by chromatography (4:1→2:1 petrol:ether) to afford the product as a white solid (0.057g; 96%); **R_f** (SiO_2; 4:1 petrol:ether) = 0.26. ^{1}H NMR (400MHz, benzene-d_6) δ_H : 7.23 (d, *J* = 7.2 Hz, 2H), 7.21 – 7.17 (m, 2H), 7.12 – 7.05 (m, 1H), 5.29 – 5.25 (m, 1H), 4.33 (m, 2H), 4.28 (m, 1H), 4.02 (s, 1H), 3.61 (d, *J* = 10.5 Hz, 1H), 3.46 (dd, *J* = 11.7, 3.7 Hz, 1H), 3.35 (d, *J* = 10.5 Hz, 1H), 2.66 (dt, *J* = 13.9, 3.4 Hz, 1H), 2.50 – 2.39 (m, 1H), 2.29 (dd, *J* = 13.6, 4.2 Hz, 1H), 1.98 – 1.59 (m, 8H), 1.58 (s, 3H), 1.54 – 1.13 (m, 8H), 1.36 (s, 3H), 1.23 (s, 3H), 1.18 (s, 3H), 1.03 (s, 3H), 1.09 – 0.81 (m, 3H), 0.91 (s, 3H), 0.89 (br. s, 6H), 0.78 – 0.69 (m, 1H). ^{13}C NMR (101MHz, benzene-d_6): δ_C 158.1, 145.5, 142.3, 130.0, 128.5, 125.3, 100.3, 79.1, 74.0, 56.8, 53.2, 49.3, 48.8, 48.4, 45.6, 43.1, 41.4, 40.3, 38.7, 38.3, 37.0, 35.4, 34.4, 33.7, 33.2, 32.2, 31.6, 31.5, 28.0, 27.4, 25.5, 25.3, 25.1, 24.3, 20.8, 19.2, 18.4, 18.0, 15.6, 14.2.

Butyl ((4a*R*,6a*R*,6b*S*,8a*S*,14b*R*)-2,2,4a,6a,6b,11,11,14b-octamethyl-4a,5,6,6a,6b,7,8,9,10, 11,12,12a,14, 14a,14b,15,16,16a-octadecahydro-4*H*-piceno[3,4-*d*][1,3]dioxin-8a(4b*H*)-yl)carbamate (MC019**)**

A solution of MC015 (0.04 g, 1 mol.eq.) in *n*-butanol (0.5 mL) was left to stand for 2 weeks at RT, the solvent being allowed to evaporate slowly to leave a cream colored, waxy residue. The residue was purified by chromatography (8:1 petrol:ether) to afford the product as a white, crystalline solid (0.042g; 92%); **R_f** (SiO_2; 4:1 petrol:ether) = 0.84. ^{1}H NMR (400MHz, benzene-d_6): δ_H 5.08 – 5.01 (m, 1H), 4.50 (s, 1H), 4.22 - 4.14 (m, 1H), 4.13 - 4.05 (m, 1H), 3.59 (d, *J* = 10.5 Hz, 1H), 3.43 (dd, *J* = 11.8, 3.8 Hz, 1H), 3.33 (d, *J* = 10.5 Hz, 1H), 2.63 – 2.52 (m, 1H), 2.30 – 2.19 (m, 1H), 2.18 – 2.10 (m, 1H), 1.92 – 1.62 (m, 7H), 1.60 – 1.07 (m, 13H), 1.57 (s, 3H), 1.34 (s, 3H), 1.20 (s, 3H),1.10 (s, 3H), 1.04 – 0.98 (m, 1H), 0.96 – 0.76

(m, 2H), 0.94 (s, 3H), 0.93 (s, 3H), 0.86 (s, 3H), 0.84 (s, 3H), 0.83 (t, *J* = 7.4 Hz, 3H), 0.73 – 0.66 (m, 1H). ^{13}C NMR (101MHz, benzene-*d*$_6$): δ$_C$ 154.7, 143.2, 124.6, 99.0, 77.9, 72.8, 64.0, 55.1, 51.9, 48.1, 46.9$_4$, 46.9$_1$, 41.7, 40.0, 39.0, 37.4, 37.0, 35.6, 33.4,33.1, 32.4, 31.8,30.9, 30.4, 26.6, 26.1, 24.0$_8$, 24.0$_5$, 23.7, 22.6, 19.5$_2$, 19.4$_9$, 17.8, 16.9, 16.6, 13.9, 13.0.

1-((4a*R*,6a*R*,6b*S*,8a*S*,14b*R*)-2,2,4a,6a,6b,11,11,14b-Octamethyl-4a,5,6,6a,6b,7,8,9,10,11, 12,12a,14,14a, 14b,15,16,16a-octadecahydro-4*H*-piceno[3,4-*d*][1,3]dioxin-8a(4b*H*)-yl)-3-propylurea (MC022**)**

A solution of MC015 (0.045 g, 1 mol.eq.) and *n*-propylamine (0.026 g, 5 mol.eq.) in anhydrous toluene (1 mL) was stirred at RT for 24 hours. The mixture was then concentrated under vacuum and the residue was purified by chromatography (2:1 petrol:ether) to afford the product as a white, crystalline solid (0.044g; 88%); **R**$_f$ (SiO$_2$; 1:1 petrol:ether) = 0.68. ^{1}H NMR (400MHz, benzene-*d*$_6$): δ$_H$ 5.36 – 5.28 (m, 1H), 4.22 (t, *J* = 5.9 Hz, 1H), 4.17 (s, 1H), 3.59 (d, *J* = 10.5 Hz, 1H), 3.46 (dd, *J* = 11.7, 3.7 Hz, 1H), 3.34 (d, *J* = 10.6 Hz, 1H), 3.26 (dq, *J* = 13.2, 6.7 Hz, 1H), 3.09 (dq, *J* = 12.9, 6.6 Hz, 1H), 2.74 (dt, *J* = 13.8, 3.3 Hz, 1H), 2.61 – 2.49 (m, 1H), 2.31 (dd, *J* = 13.5, 4.1 Hz, 1H), 2.03 – 1.80 (m, 6H), 1.68 (m, 2H), 1.57 (s, 3H), 1.55 – 1.18 (m, 9H), 1.35 (s, 3H), 1.22 (s, 3H), 1.21 (s, 3H), 1.09-0.99 (m, 2H), 1.06 (s, 3H), 1.00 (s, 3H), 0.94 (s, 3H), 0.92 (s, 3H), 0.91-0.85 (m, 1H), 0.88 (t, *J* = 7.4 Hz, 3H), 0.78 – 0.72 (m, 1H). ^{13}C NMR (101MHz, benzene-*d*$_6$): δ$_C$ 157.2, 144.4, 124.1, 99.1, 77.9, 72.7, 55.3, 51.9, 48.1, 47.8, 47.1, 42.1, 41.9, 40.2, 39.1, 37.5, 37.1, 35.9, 34.3, 33.2, 32.4, 31.0,30.4, 26.9, 26.2, 24.3$_6$, 24.3$_2$, 24.1, 23.9, 23.0, 19.5, 17.9, 17.1, 16.7, 13.0, 11.6.

***N*-((4a*R*,6a*R*,6b*S*,8a*S*,14b*R*)-2,2,4a,6a,6b,11,11,14b-Octamethyl-4a,5,6,6a,6b,7,8,9,10,11,12,12a,14,14a, 14b,15,16,16a-octadecahydro-4*H*-piceno[3,4-*d*][1,3]dioxin-8a(4b*H*)-yl)- pyrolidine-1-carboxamide (**MC023**)**

A solution of MC015 (0.045 g, 1 mol.eq.) and pyrrolidine (0.037 mL) in anhydrous toluene (1 mL) was stirred at RT for 18 hours. The mixture was then concentrated under vacuum and the residue was purified by chromatography (application of residue to silica in DCM; 1:1 petrol:ether→10:1 chloroform:methanol) to afford the product [15], as a white, resinous solid (0.045g; 88%); **R**$_f$ (SiO$_2$; 1:1 petrol: ether) = 0.44. ^{1}H NMR (400MHz, benzene-*d*$_6$): δ$_H$ 5.35 – 5.25 (m, 1H), 4.05 (s, 1H), 3.59 (d, *J* = 10.5 Hz, 1H), 3.44 (dd, *J* = 11.7, 3.8 Hz, 1H), 3.34 (d, *J* = 10.5 Hz, 1H), 3.29 – 3.11 (m, 4H), 2.91 (dt, *J* = 13.9, 3.4 Hz, 1H), 2.70-2.57 (m, 1H), 2.48 – 2.34 (m, 1H), 2.09 – 1.75 (m, 6H), 1.75 – 1.52 (m, 2H), 1.57 (s, 3H), 1.51 – 1.13 (m, 12H), 1.35 (s, 3H), 1.21 (s, 3H), 1.17 (s, 3H), 1.04 (s, 3H), 1.03-0.81 (m, 3H), 0.95 (s, 3H), 0.91 (s, 3H), 0.86 (s, 3H), 0.71 (dd, *J* = 11.6, 2.1 Hz, 1H). ^{13}C NMR (101MHz, benzene-*d*$_6$): δ$_C$ 155.6, 144.5, 124.0, 99.0, 77.9, 72.7, 55.6, 51.9, 48.08, 47.91, 47.4, 45.8, 42.0, 40.1, 39.0, 37.4, 37.1, 35.8, 34.1, 33.2, 32.2, 31.0, 30.4, 26.7, 26.0, 25.8, 24.2, 24.1, 23.8, 22.9, 19.5, 17.9, 17.1, 16.6, 13.0. [M+H]$^+$: 581.4676. Calculated for $C_{37}H_{61}O_3N_2$: 581.4677.

1-((4a*R*,6a*R*,6b*S*,8a*S*,14b*R*)-2,2,4a,6a,6b,11,11,14b-Octamethyl-4a,5,6,6a,6b,7,8,9,10,11, 12, 12a,14,14a, 14b,15,16,16a-octadecahydro-4*H*-piceno[3,4-*d*][1,3]dioxin-8a(4b*H*)- yl)urea (MC024**)**

A gentle stream of ammonia gas was passed through a solution of MC015 (0.045 g, 1mol.eq.) over 30 minutes. The occasional addition of solvent was necessary in order to compensate for evaporative losses. The percolation of gas was then suspended, and the flask was sealed with a greased glass stopper. Vigorous stirring was maintained at RT for 18 hours. The mixture was then concentrated under vacuum and the residue was purified by chromatography (10:1 chloroform:methanol) to afford the product as a white powder (0.037g; 80%); **R**$_f$ (SiO$_2$; 10:1 chloroform:methanol) = 0.54. ^{1}H NMR (400MHz, $CDCl_3$): δ$_H$ 5.20 (m, 1H), 4.35 (s, 1H), 3.44 (d, *J* = 10.6 Hz, 1H), 3.43 – 3.38 (m, 1H), 3.35 (d, *J* = 10.6 Hz, 1H), 2.21 (dt, *J* = 13.8, 3.5 Hz, 1H), 2.04 – 1.92 (m, 2H), 1.80 (m, 3H), 1.70 – 1.46 (m, 6H), 1.38 – 1.29 (m, 3H), 1.35 (s, 3H), 1.32 (s, 3H), 1.25 – 1.01 (m, 4H), 1.05 (s, 3H), 0.98 – 0.77 (m, 3H), 0.96 (s, 3H), 0.86 (s, 3H), 0.84 (s, 3H), 0.80 (br. s, 6H), 0.74 – 0.67 (m, 1H). ^{13}C NMR (101MHz,

$CDCl_3$): δ_C 157.7, 143.3, 124.6, 99.1, 77.7, 72.7, 55.8, 51.6, 47.7, 47.5, 46.5, 41.7,39.9, 39.0, 37.3, 36.9, 35.3, 33.3, 33.0, 32.0, 30.9, 30.0, 26.3, 26.0, 24.1, 23.6_1, 23.5_9, 22.3, 19.5, 17.8, 16.8, 16.6, 12.6.

Methyl (((4a*R*,6a*R*,6b*S*,8a*S*,14b*R*)-2,2,4a,6a,6b,11,11,14b-octamethyl-4a,5,6,6a,6b,7,8,9,10, 11,12,12a, 14,14a,14b,15,16,16a-octadecahydro-4*H*-piceno[3,4-*d*][1,3]dioxin-8a(4b*H*)-yl)-carbamoyl)-L-valinate (MC027**)**

A solution of L-valine methyl ester hydrochloride (0.044 g, 5 mol.eq.) and NEt_3 (0.038 mL, 5.1 mol.eq.) in dry toluene (2.5mL) was stirred at RT for 10 minutes prior to the addition of a solution of MC015 (0.027 g, 1 mol.eq.) in dry toluene (2.5mL). The mixture was stirred at RT for 18 hours, then concentrated under vacuum and the residue purified by chromatography (4:1→1:1 petrol:ether) to afford the product as a white, resinous solid (0.035g; quant.); $\mathbf{R_f}$ (SiO_2; 4:1 petrol:ether) = 0.17. ^{1}H NMR (400MHz, benzene-*d*$_6$): δ_H 5.27 – 5.19 (m, 1H), 4.78 (dd, *J* = 8.8, 4.7 Hz, 1H), 4.66 (d, *J* = 8.8 Hz, 1H), 4.08 (s, 1H), 3.60 (d, *J* = 10.5 Hz, 1H), 3.45 (dd, *J* = 11.7, 3.8 Hz, 1H), 3.34 (d, *J* = 10.5 Hz, 1H), 3.29 (s, 3H), 2.64 (dt, *J* = 13.9, 3.3 Hz, 1H), 2.39 – 2.26 (m, 2H), 2.10 (pd, *J* = 6.9, 4.8 Hz, 1H), 2.02 – 1.54 (m, 8H), 1.58 (s, 3H), 1.53 – 1.40 (m, 2H), 1.39 – 1.10 (m, 5H), 1.35 (s, 3H), 1.21 (s, 3H), 1.15 (s, 3H), 1.07-0.87 (m, 3H), 1.04 (s, 3H), 1.02 (s, 3H), 0.98 (d, *J* = 6.8 Hz, 3H), 0.89 (s, 3H), 0.88 (s, 3H), 0.85 (d, *J* = 6.9 Hz, 3H), 0.75 – 0.69 (m, 1H). ^{13}C NMR (101MHz, benzene-*d*$_6$): δ_C 174.1, 156.4, 144.1, 124.3, 99.0, 77.9,72.8, 57.9, 55.6, 51.9, 51.5, 48.1, 45.8, 47.2, 41.8, 40.2,39.1, 37.4, 37.1, 35.8, 34.0, 33.1, 32.3, 31.8, 31.0, 30.4, 26.7, 26.2, 24.3, 24.1, 23.8, 22.9, 19.5, 19.3, 17.96, 17.91, 16.9, 16.7, 13.0.

1-((4a*R*,6a*R*,6b*S*,8a*S*,14b*R*)-2,2,4a,6a,6b,11,11,14b-Octamethyl-4a,5,6,6a,6b,7,8,9,10,11, 12,12a,14,14a, 14b,15,16,16a-octadecahydro-4*H*-piceno[3,4-*d*][1,3]dioxin-8a(4b*H*)-yl)-3-((3*R*,4*R*,5*S*,6*R*)-2,4,5-tri-hydroxy-6-(hydroxymethyl)tetrahydro-2*H*-pyran-3-yl)urea (MC028**)**

A mixture of D-(+)-glucosamine hydrochloride (0.022 g, 1.05 mol.eq.) and NEt_3 (0.015 mL) in dry DMF (1 mL) was stirred at RT for 5–10 minutes prior to the addition of solid MC015 (0.05 g, 1 mol.eq.) in one portion. The mixture was heated to 50°C for 2–4 hours, whereupon the DMF was removed under high vacuum and the residue was purified by chromatography (10:1 chloroform:methanol) to afford the product [15], as a white crystalline solid (0.065g; 96%); $\mathbf{R_f}$ (SiO_2; 10:1 chloroform:methanol) = 0.28.

((4a*R*,6a*R*,6b*S*,8a*S*,14b*R*)-2,2,4a,6a,6b,11,11,14b-Octamethyl-4a,5,6,6a,6b,7,8,9,10,11,12,12a,14,14a, 14b,15,16,16a-octadecahydro-4*H*-piceno[3,4-*d*][1,3]dioxin-8a(4b*H*)-yl)methanol (MC029**)**

A solution of MC014 (0.067 g, 1 mol.eq) in dry THF (15.0 mL) was added dropwise over 1–2 min to a suspension of $LiAlH_4$ (0.25 g, 5 mol.eq) in dry THF (5.0mL) cooled to ≈ +10°C. The mixture was heated under reflux at 85°C for 2–3 hours, then cooled to 0°C and quenched carefully with sat.aq. sodium sulphate (≈ 5-10mL), diluted with Et_2O (15mL) and stirred vigorously for 0.5–1 hour at RT. The mixture was then filtered, by suction, through a plug of celite, the plug being washed thoroughly with Et_2O (20mL) and warm chloroform (15 mL). The filtrate was concentrated under vacuum and the residue was purified by chromatography (2:1 petrol:ether) to afford the product as a white solid (0.59g; 91%); $\mathbf{R_f}$ (SiO_2; 5:1 petrol:ether) = 0.55. ^{1}H NMR (400MHz, benzene-*d*$_6$): δ_H 5.19 (t, *J* = 3.7 Hz, 1H), 3.60 (d, *J* = 10.5 Hz, 1H), 3.54 (d, *J* = 10.6 Hz, 1H), 3.44 (dd, *J* = 11.7, 3.7 Hz, 1H), 3.34 (d, *J* = 10.5 Hz, 1H), 3.14 (d, *J* = 10.6 Hz, 1H), 2.09 (dd, *J* = 13.7, 4.3 Hz, 1H), 1.93-1.52 (m, 9H), 1.57 (s, 3H), 1.51-1.14 (m, 9H), 1.34 (s, 3H), 1.24 (s, 3H), 1.21 (s, 3H), 1.07 – 0.99 (m, 1H), 0.98 – 0.91 (m, 1H), 0.94 (br. s, 9H), 0.87 – 0.78 (m, 1H), 0.86 (s, 3H), 0.72 (dd, *J* = 12.1, 2.0 Hz, 1H). ^{13}C NMR (101MHz, benzene-*d*$_6$): δ_C 144.6, 122.6, 99.0, 77.9, 72.8, 69.4, 51.9, 48.1, 47.0, 42.8, 42.0, 40.3, 39.2, 37.3_6, 37.3_0, 37.1, 34.6, 33.5, 32.5, 31.5, 31.2, 30.4, 26.2, 26.1, 24.1, 23.8_4, 23.7_6, 22.5, 19.5, 17.9, 16.9_1, 16.8_4, 13.0.

1-((4a*S*,6a*S*,6b*R*,9*R*,10*S*,12a*R*)-10-Hydroxy-9-(hydroxymethyl)-2,2,6a,6b,9,12a-hexa- methyl-1,3,4,5,6, 6a,6b,7,8,8a,9,10,11,12,12a,12b,13,14b-octadecahydropicen-4a(2*H*)-yl)- 3-((3*R*,4*R*,5*S*,6*R*)-2,4,5-tri-hydroxy-6-(hydroxymethyl)tetrahydro-2*H*-pyran-3-yl)urea (MC030**)**

To a solution of MC028 (0.0325 g) in THF (1 mL), H_2O (0.1 mL) was added, followed by HCl/Et_2O (2M, 0.15 mL). The mixture was stirred vigorously at RT for 1.5 hours. The mixture was then concentrated under vacuum and the orange oily residue was purified by chromatography (85:12.5:2.5 chloroform:methanol:H_2O) to afford the principal product as a white crystalline solid (0.02g; 65%); $\mathbf{R_f}$ (SiO_2; 75:22.5:2.5 chloroform:methanol: H_2O) = 0.62. A minor product (0.01 g) was also isolated, a white solid (0.01g); $\mathbf{R_f}$ (SiO_2; 75:22.5:2.5 chloroform: methanol: H_2O) = 0.49.

((4a*R*,6a*R*,6b*S*,8a*S*,14b*R*)-2,2,4a,6a,6b,11,11,14b-Octamethyl-4a,5,6,6a,6b,7,8,9,10,11,12,12a,14,14a, 14b,15,16,16a-octadecahydro-4*H*-piceno[3,4-*d*][1,3]dioxin-8a(4b*H*)-yl)methyl acetate (MC031**)**

To a solution of MC029 (0.04 g, 1 mol.eq.), NEt_3 (0.012 mL, 1.05 mol.eq.) and DMAP (0.01 g, 1 mol.eq.) in dry DCM (1.0 mL), cooled to ≈ +5°C, acetyl chloride (0.0066 g, 1.05 mol.eq.) was added dropwise over a period of 1–2 minutes. The mixture was stirred for 2 hours with gradual warming to RT. The mixture was adsorbed on silica and the material was applied directly to a silica column; chromatography (10:1 petrol:ether) afforded the product as a clear, colorless oil (0.030g; 69%); $\mathbf{R_f}$ (SiO_2; 10:1 petrol: ether) = 0.43. ^{1}H NMR (400MHz, benzene-d_6): δ_H 5.19 (t, *J* = 3.7 Hz, 1H), 4.30 (d, *J* = 11.1 Hz, 1H), 3.86 (d, *J* = 11.1 Hz, 1H), 3.59 (d, *J* = 10.5 Hz, 1H), 3.43 (dd, *J* = 11.8, 3.7 Hz, 1H), 3.33 (d, *J* = 10.5 Hz, 1H), 2.12 (dd, *J* = 13.6, 4.4 Hz, 1H), 1.93 (td, *J* = 13.6, 4.2 Hz, 1H), 1.86 – 1.71 (m, 8H), 1.75 (s, 3H), 1.57 (s, 3H), 1.51 – 1.09 (m, 9H), 1.33 (s, 3H), 1.21 (s, 3H), 1.19 (s, 3H), 1.03 – 0.77 (m, 3H), 0.98 (s, 3H), 0.93 (s, 3H), 0.90 (s, 3H), 0.81 (s, 3H), 0.71 (dd, *J* = 12.0, 2.0 Hz, 1H). ^{13}C NMR (101MHz, benzene-d_6): δ_C 170.2, 143.8, 123.3, 99.0, 77.9, 72.8, 70.7, 52.0, 48.1, 46.7, 43.1, 41.9, 40.3, 39.2, 37.3, 37.1, 36.2, 34.4, 33.4, 32.5, 31.9, 31.1, 30.4, 26.3, 26.1, 24.1, 23.76, 23.73, 22.6, 20.6, 19.5, 17.9, 16.9, 16.8, 13.0.

((4a*R*,6a*R*,6b*S*,8a*S*,14b*R*)-2,2,4a,6a,6b,11,11,14b-Octamethyl-4a,5,6,6a,6b,7,8,9,10,11,12,12a,14,14a, 14b,15,16,16a-octadecahydro-4*H*-piceno[3,4-*d*][1,3]dioxin-8a(4b*H*)-yl)methyl palmitate (MC032**)**

To a solution of MC029 (0.04 g, 1 mol.eq), NEt_3 (0.012 mL) and DMAP (0.02 g, 1 mol.eq) in dry DCM (2 mL), cooled to ≈ +5°C, palmitoyl chloride (0.023 g, 1.05 mol.eq.) was added dropwise over 1–2 minutes. The mixture was stirred for 2 hours with gradual warming to RT, then adsorbed on silica and applied directly to a silica column; chromatography (20:1 petrol:ether) afforded the product as a clear, colorless oil (0.054g; 91%); $\mathbf{R_f}$ (SiO_2; 10:1 petrol:ether) = 0.87. Repetition of the chromatographic purification was deemed necessary in order to give a sample of acceptable purity. ^{1}H NMR (400MHz, benzene-d_6): δ_H 5.22 (t, *J* = 3.6 Hz, 1H), 4.33 (d, *J* = 11.0 Hz, 1H), 3.94 (d, *J* = 11.1 Hz, 1H), 3.59 (d, *J* = 10.5 Hz, 1H), 3.44 (dd, *J* = 11.7, 3.6 Hz, 1H), 3.34 (d, *J* = 10.5 Hz, 1H), 2.26 (t, *J* = 7.3 Hz, 2H), 2.17 (dd, *J* = 13.5, 4.4 Hz, 1H), 1.98 (td, *J* = 13.6, 4.2 Hz, 1H), 1.91 - 1.38 (m, 46H), 1.57 (s, 3H), 1.23 (s, 3H), 1.20 (s, 3H), 1.05 - 0.95 (m, 2H), 1.01 (s, 3H), 0.94 (s, 3H),0.93 (s, 3H), 0.92 (t, *J* = 7.0 Hz, 3H), 0.87 - 0.78 (m, 1H), 0.80 (s, 3H), 0.75 – 0.68 (m, 1H). ^{13}C NMR (101MHz, benzene-d_6): δ_C 173.1, 143.9, 123.4, 99.0, 77.9,72.8, 70.6, 52.0, 48.1, 46.8, 43.0, 41.9, 40.3, 39.2, 37.3, 37.1, 36.3, 34.6, 34.5, 33.4, 32.5, 32.4, 32.0, 31.1, 30.4, 30.2_4, 30.2_2, 30.1_9, 30.1_3, 30.0_4, 29.9, 29.8, 29.6, 26.3, 26.1, 25.6, 24.1, 23.7_8, 23.7_7, 23.2, 22.8, 19.5, 17.9, 17.0, 16.8, 14.4, 13.0.

(((4a*R*,6a*R*,6b*S*,8a*S*,14b*R*)-2,2,4a,6a,6b,11,11,14b-Octamethyl-4a,5,6,6a,6b,7,8,9,10,11,12,12a,14,14a, 14b,15,16,16a-octadecahydro-4*H*-piceno[3,4-*d*][1,3]dioxin-8a(4b*H*)-yl)carbam- oyl)-L-proline (MC033**)**

A mixture of MC015 (0.031 g, 1 mol.eq.) and L-proline (0.035 g, 5 mol.eq.) in absolute ethanol (1.3 mL) was heated briefly to reflux (1–2 minutes) in order to effect the dissolution of the amino acid. The mixture was then stirred vigorously for 19 hours at RT. The mixture was then concentrated under vacuum and the residue was purified by chromatography (10:1 chloroform:methanol) to afford the product [15], as a white, waxy solid (0.038g; quant.); $\mathbf{R_f}$ (SiO_2; 10:1 chloroform:methanol) = 0.43. ^{1}H NMR (400MHz, pyridine-*d*$_5$: δ_H 5.41 – 5.31 (m, 1H), 5.01 – 4.71 (m, 2H), 3.72 – 3.59 (m, 3H), 3.59 – 3.42 (m, 2H), 2.74 – 2.62 (m, 1H), 2.61 – 2.47 (m, 2H), 2.29 – 1.60 (m, 12H), 1.59 – 0.82 (m, 11H), 1.53 (s, 3H), 1.48 (s, 3H), 1.20 (s, 3H), 1.19 (br. s, 6H), 0.96 (s, 3H), 0.94 (s, 3H), 0.89 (s, 3H). ^{13}C NMR (101MHz, pyridine-*d*$_5$): δ_C 156.7, 144.7, 124.5, 99.5, 80.3, 78.1, 73.0, 60.4, 56.4, 51.9, 48.4, 47.7, 47.6, 46.8, 42.4, 40.6, 39.4, 37.9, 37.5, 36.2, 34.5, 33.5, 32.7, 31.4, 30.8, 30.6, 27.1, 26.6, 25.6, 24.6, 24.5, 24.3, 23.3, 20.1, 18.4, 17.6, 17.1, 13.4. [M+H]$^+$: 625.4583. Calculated for $C_{38}H_{61}O_5N_2$: 625.4575.

***N*-((4a*R*,6a*R*,6b*S*,8a*S*,14b*R*)-2,2,4a,6a,6b,11,11,14b-Octamethyl-4a,5,6,6a,6b,7,8,9,10,11,12,12a,14,14a, 14b,15,16,16a-octadecahydro-4*H*-piceno[3,4-*d*][1,3]dioxin-8a(4b*H*)-yl)piper- azine-1-carboxamide (**MC035**)**

A solution of MC015 (0.03g, 0.059mmol) and piperazine (0.006g, 0.065mmol) in anhydrous toluene (1.0mL) was allowed to stir at RT for 19 hours. The reaction mixture was then concentrated *in vacuo* and the residue was purified by column chromatography (1:1→ 0:1 petrol:ethyl acetate → 10:1 chloroform:methanol; silica NEt_3 neutralized), to afford the principal product as a white solid (0.026g; 75%); R_f (SiO_2; 10:1 chloroform:methanol) = 0.30; ^{1}H NMR (400MHz, benzene-*d*$_6$) δ_H : 5.26 (t, *J* = 3.6 Hz, 1H), 4.30 (s, 1H), 3.60 (d, *J* = 10.5 Hz, 1H), 3.49 – 3.40 (m, 1H), 3.34 (d, *J* = 10.5 Hz, 1H), 3.29 – 3.17 (m, 4H), 2.90 (dt, *J* = 13.9, 3.4 Hz, 1H), 2.65 – 2.56 (m, 1H), 2.56 – 2.50 (m, 1H), 2.49 – 2.42 (m, 4H), 2.32 (dd, *J* = 13.5, 4.2 Hz, 1H), 1.97 – 1.75 (m, 6H), 1.74 – 1.55 (m, 2H), 1.58 (s, 3H), 1.52 – 1.44 (m, 1H), 1.44 - 1.11 (m, 6H), 1.36 (s, 3H), 1.24 (s, 3H), 1.15 (s, 3H), 1.09 – 0.76 (m, 3H), 0.99 (s, 3H), 0.93 (s, 3H), 0.90 (s, 3H), 0.86 (s, 3H), 0.74 – 0.68 (m, 1H); ^{13}C NMR (101MHz, benzene-*d*$_6$) δ_C: 156.3, 144.3, 124.3, 99.1, 77.9, 72.7, 55.7, 51.9, 48.0$_8$, 47.9$_9$, 47.2, 46.0, 45.1, 41.9, 40.1, 39.0, 37.4, 37.1, 35.7, 33.7, 33.1, 32.1, 31.0, 30.4, 26.7, 26.1, 24.1$_9$, 24.0$_7$, 23.8, 22.6, 19.5, 17.9, 17.0, 16.6, 13.0.

((4a*S*,6a*S*,6b*R*,9*R*,10*S*,12a*R*)-10-Hydroxy-9-(hydroxymethyl)-2,2,6a,6b,9,12a-hexamethyl-1,3,4,5,6,6a, 6b,7,8,8a,9,10,11,12,12a,12b,13,14b-octadecahydropicen-4a(2*H*)-yl)methyl acetate (MC037**)**

To a solution of MC031 (0.012 g, 1.mol.eq.) in DCM (0.5 mL), H_2O (0.05 mL) was added, followed by HCl/Et_2O solution (2 M, 0.15 mL). The mixture was stirred vigorously at RT for 0.5 hour. The mixture was then concentrated under vacuum and the residue was purified by chromatography (10:1 chloroform:methanol) to afford the principal product as a white solid (0.011g; quant.); $\mathbf{R_f}$ (SiO_2; 10:1 chloroform:methanol) = 0.42; ^{1}H NMR (400MHz, benzene-*d*$_6$): δ_H 5.20 (t, *J* = 3.7 Hz, 1H), 4.32 (d, *J* = 11.1 Hz, 1H), 3.86 (d, *J* = 11.1 Hz, 1H), 3.54 (d, *J* = 10.5 Hz, 1H), 3.51 (dd, *J* = 12.0, 4.0 Hz, 1H), 3.20 (d, *J* = 10.2 Hz, 1H), 5.20 (br. S, 1H), 2.23 (s, 1H), 2.12 (dd, *J* = 13.7, 4.4 Hz, 1H), 1.98 – 1.70 (m, 5H), 1.74 (s, 3H), 1.67 – 1.37 (m, 6H), 1.37 – 1.10 (m, 7H), 1.18 (s, 3H), 1.08 – 0.67 (m, 4H), 1.01 (s, 3H), 0.92 (s, 3H), 0.90 (s, 3H), 0.85 (s, 3H), 0.84 (s, 3H). ^{13}C NMR (101MHz, benzene-*d*$_6$): δ_C 170.3, 143.9, 123.4, 76.1, 71.4, 70.8, 49.8, 48.0, 46.7, 43.1, 42.1, 41.9, 40.1, 38.6, 37.0, 36.2, 34.3, 33.4, 32.7, 31.9, 31.1, 27.2, 26.2, 26.1, 23.9, 23.7, 22.6, 20.5, 18.7, 17.0, 16.0, 11.9.

((4a*S*,6a*S*,6b*R*,9*R*,10*S*,12a*R*)-10-Hydroxy-9-(hydroxymethyl)-2,2,6a,6b,9,12a-hexamethyl-1,3,4,5,6,6a, 6b,7,8,8a,9,10,11,12,12a,12b,13,14b-octadecahydropicen-4a(2*H*)-yl)methyl palmitate (MC038**)**

To a solution of MC032 (0.0134 g, 1 mol.eq.) in DCM (0.5 mL), H_2O (0.05 mL) was added, followed by HCl/Et_2O solution (2 M, 0.15 mL). The mixture was stirred vigorously at RT for 0.75 hour, then concentrated under vacuum and the residue was purified by chromatography (10:1 chloroform:methanol) to afford the principal product as a clear,

colorless oil (0.008g; 63%); **R$_f$** (SiO$_2$; 10:1 chloroform:methanol) = 0.51. ^{1}H NMR (400MHz, benzene-*d$_6$*): 5.23 (t, *J* = 3.6 Hz, 1H), 4.34 (d, *J* = 11.1 Hz, 1H), 3.94 (d, *J* = 11.1 Hz, 1H), 3.54 (d, *J* = 10.6 Hz, 1H), 3.50 (dd, *J* = 12.0, 8.0 Hz, 1H), 3.20 (d, *J* = 10.2 Hz, 1H), 2.25 (t, *J* = 7.4 Hz, 2H), 2.21 – 2.13 (m, 2H), 1.96 (td, *J* = 13.4, 4.0 Hz, 1H), 1.91 – 1.61 (m, 8H), 1.60 –1.15 (m, 37H), 1.19 (s, 3H), 1.06 – 0.74 (m, 3H), 1.04 (s, 3H), 0.93 (m, 9H), 0.85$_4$ (s, 3H), 0.85$_0$ (s, 3H). ^{13}C NMR (101MHz, benzene-*d$_6$*): 173.2, 143.9, 123.4, 76.1, 71.4, 70.6, 49.8, 48.1, 46.7, 43.0, 42.1$_2$, 41.9$_5$, 40.1$_2$, 38.7, 37.0, 36.3, 34.6$_2$, 34.4$_3$, 33.4, 32.7, 32.4, 32.0, 31.1, 30.2$_3$, 30.2$_1$, 30.1$_8$, 30.1$_2$, 30.0$_2$, 29.8$_7$, 29.7$_7$, 29.5$_8$, 27.3, 26.2$_3$, 26.1$_1$, 25.6, 23.9, 23.8, 23.2, 22.9, 18.7, 17.1, 16.0, 14.4, 11.9.

_N_-((4a*R*,6a*R*,6b*S*,8a*S*,14b*R*,16a*S*)-2,2,4a,6a,6b,11,11,14b-Octamethyl-4a,5,6,6a,6b,7,8,9,10,11,12,12a, 14,14a,14b,15,16,16a-octadecahydro-4*H*-piceno[3,4-*d*][1,3]dioxin-8a(4b*H*)-yl)- morpholine-4-carboxamide (MC042**)**

A solution of MC015 (0.04g, 0.078mmol) and morpholine (0.0082g, 0.0083mL, 0.094mmol) in anhydrous toluene (1.0mL) was stirred at RT for 19 hours. The reaction mixture was then concentrated *in vacuo* and the residue was purified by column chromatography (1:1 petrol:ethyl acetate), to afford the product as a white, resinous solid (0.049g; quant.) [15]; R$_f$ (SiO$_2$; 10:1 chloroform:methanol) = 0.64; ^{1}H NMR (400MHz, benzene-*d$_6$*) δ$_H$: 5.31 – 5.23 (m, 1H), 4.23 (s, 1H), 3.61 (d, *J* = 10.5 Hz, 1H), 3.45 (dd, *J* = 11.8, 3.9 Hz, 1H), 3.40 – 3.29 (m, 5H), 3.21 – 3.07 (m, 4H), 2.86 (dt, *J* = 14.0, 3.4 Hz, 1H), 2.62 – 2.53 (m, 1H), 2.32 (dd, *J* = 13.6, 4.2 Hz, 1H), 1.98 – 1.72 (m, 6H), 1.68 (dd, *J* = 12.5, 3.3 Hz, 1H), 1.64 – 1.55 (m, 1H), 1.58 (s, 3H), 1.53 – 1.44 (m, 1H), 1.43 – 1.08 (m, 7H), 1.36 (s, 3H), 1.24 (s, 3H), 1.15 (s, 3H), 1.06 – 0.77 (m, 3H), 1.00 (s, 3H), 0.90 (s, 3H), 0.89 (s, 3H), 0.85 (s, 3H), 0.75 – 0.68 (m, 1H); ^{13}C NMR (101MHz, benzene-*d$_6$*) δ$_C$: 156.2, 144.2, 124.3, 99.1, 77.9, 72.7, 66.7, 55.8, 51.9, 48.0$_1$, 47.9$_1$, 47.2, 44.6, 41.9, 40.1, 39.0, 37.4, 37.1, 35.7, 33.7, 33.1, 32.1, 31.0, 30.4, 26.7, 26.1, 24.1$_7$, 24.0$_7$, 23.8, 22.5, 19.5, 17.9, 17.0, 16.6, 13.0.

1, 3-bis(*tert*-Butoxycarbonyl) guanidine urea derivative of MC015 (MC044**)**

A solution of MC015 (0.03 g, 1 mol.eq.) and 1,3-bis(*tert*-butoxycarbonyl) guanidine (0.017 g, 1.1 mol.eq.) in anhydrous toluene (1.0 mL) was allowed to stir at RT for 18 hours. The mixture was then concentrated under vacuum and the residue was purified by chromatography (15:1→5:1 petrol:ether) to afford the product as a white, resinous solid (0.038g; 84%); **R$_f$** (SiO$_2$; 10:1 petrol:ether) =0 .38.

Methyl (((4a*R*,6a*R*,6b*S*,8a*S*,14b*R*,16a*S*)-2,2,4a,6a,6b,11,11,14b-octamethyl-4a,5,6,6a,6b,7, 8,9,10,11,12, 12a,14,14a,14b,15,16,16a-octadecahydro-4*H*-piceno[3,4-*d*][1,3]dioxin-8a(4b*H*)-yl)carbamoyl)-L-histidinate (MC048**)**

A suspension of L-histidine methyl ester dihydrochloride (0.016 g, 1.1 mol.eq.) and NEt$_3$ (0.02 mL, 2.42.mol.eq.) in dry DCM (0.3mL) was stirred at RT for 5 minutes prior to the addition of a solution of MC015 (0.03 g, 1 mol.eq.) in dry DCM (1.0mL). The reaction was stirred at RT for 18 hours, whereupon the mixture was concentrated under vacuum and the residue was purified by chromatography (10:1 chloroform:methanol; silica NEt$_3$ neutralized) to afford the principal product as a white solid (0.025g; 63%); **R$_f$** (SiO$_2$; 10:1 chloroform:methanol) = 0.36. A secondary product was also isolated; **R$_f$** (SiO$_2$; 10:1 chloroform:methanol)=0.71. ^{1}H NMR (400MHz, methanol-*d$_4$*): δ$_H$ 7.59 (d, *J* = 1.1 Hz, 1H), 6.85 (d, *J* = 1.1 Hz, 1H), 5.50 (d, *J* = 1.8 Hz, 1H), 5.29 – 5.25 (m, 1H), 4.48 (dd, *J* = 7.6, 5.4 Hz, 1H), 3.68 (s, 3H), 3.62 (dd, *J* = 11.9, 3.8 Hz, 1H), 3.53 (s, 2H), 3.02 (dd, *J* = 14.9, 5.4 Hz, 1H), 2.95 (dd, *J* = 14.9, 7.6 Hz, 1H), 2.30 (dd, *J* = 13.6, 4.2 Hz, 1H), 2.07 (dt, *J* = 13.7, 3.4 Hz, 1H), 2.03 – 1.27 (m, 15H), 1.45 (s, 3H), 1.35 (s, 3H), 1.27 – 1.08 (m, 3H), 1.18 (s, 3H), 1.06 (s, 3H), 1.03 (s, 3H), 1.00 – 0.80 (m, 2H), 0.97 (s, 3H), 0.94 (s, 3H), 0.90 (s, 3H). ^{13}C NMR (101MHz, methanol-*d$_4$*): δ$_C$ 174.5, 159.0, 144.8, 136.2, 124.9, 100.4, 78.9, 73.5, 56.4, 54.1, 52.5, 47.9, 47.8, 42.7, 41.0, 39.9, 38.3, 37.9, 36.4, 34.7, 33.3$_8$, 33.3$_2$, 31.7, 30.8, 30.1, 27.3, 26.5, 24.6, 24.5, 24.4, 23.8, 19.7, 18.7, 17.5, 17.1, 13.0.

***N*-((4a*S*,6a*S*,6b*R*,9*R*,10*S*,12a*R*)-10-Hydroxy-9-(hydroxymethyl)-2,2,6a,6b,9,12a-hexamethyl-1,3,4,5,6, 6a,6b,7,8,8a,9,10,11,12,12a,12b,13,14b-octadecahydropicen-4a(2*H*)-yl)pyrrol- idine-1-carboxamide (**MC055**)**

To a solution of MC023 (0.05g, 0.086mmol) in dichloromethane (1.0mL), H_2O (0.05 mL) was added, followed by HCl/Et_2O solution (2M solution; 0.05mL, 0.1mmol). The mixture was stirred vigorously at RT for 45 minutes. Hydrochloric acid (2M aq. soln.; 5 drops) was then administered, and the reaction mixture was stirred vigorously for a further period of 1 hour at room temperature. The mixture was then concentrated *in vacuo* and the residue was purified by column chromatography (20:1 chloroform:methanol; silica NEt_3 neutralized), to afford the product [15], as a white solid (0.046g; quant.); R_f (SiO_2; 10:1 chloroform:methanol) = 0. 40; ^{1}H NMR (400MHz, chloroform-*d*) δ_H : 5.30 (t, *J* = 2.3 Hz, 1H), 4.33 (s, 1H), 3.72 (d, *J* = 10.2 Hz, 1H), 3.67 – 3.60 (m, 1H), 3.42 (d, *J* = 10.2 Hz, 1H), 3.33 – 3.21 (m, 4H), 2.67 (br. s, 2H), 2.38 (dt, *J* = 13.7, 3.5 Hz, 1H), 2.23 – 2.10 (m, 2H), 1.96 – 1.82 (m, 7H), 1.81 – 1.54 (m, 7H), 1.52 – 1.10 (m, 7H), 1.13 (s, 3H), 1.05 – 0.74 (m, 3H), 0.96 (s, 3H), 0.94 (s, 3H), 0.90 (s, 3H), 0.89 (br. s, 6H); ^{13}C NMR (101MHz, chloroform-*d*) δ_C : 155.5, 143.5, 124.2, 77.0, 72.4, 56.3, 50.0, 47.7, 47.4, 46.9, 46.5, 41.9, 41.8, 39.7, 38.3, 37.0, 35.4, 33.4, 33.0, 32.2, 30.9, 26.7, 26.3, 25.9, 25.7, 24.1, 23.7, 22.6, 18.6, 17.1, 15.8, 11.6; [M+H]$^+$: 541.4361. $C_{34}H_{57}O_3N_2$ requires: 541.4364

((3*S*,4*R*,6a*R*,6b*S*,8a*S*,14b*R*)-3-Hydroxy-4,6a,6b,11,11,14b-hexamethyl-8a-(pyrrolidine-1-carbox-amido)-1,2,3,4,4a,5,6,6a,6b,7,8,8a,9,10,11,12,12a,14,14a, 14b-icosahydropicen-4-yl)- methyl acetate (MC057**)**

To a solution of MC055 (0.03 g, 1 mol.eq.), NEt_3 (0.0089 mL) and DMAP (0.0007 g) in dry DCM (1.2 mL), cooled to ≈ +5°C, a solution of acetyl chloride (0.0043 mL) in dry DCM (0.2 mL) was added dropwise over 2–3 minutes. The mixture was stirred at ≈ +5°C for 0.75 hour, then concentrated under vacuum and the residue was purified by chromatography (1:1 → 2:3 petrol:ether; silica NEt_3 neutralized) to afford the principal product [15] as a white solid (0.018g; 56%); **R**$_f$ (SiO_2; 1:1 petrol:ether) = 0.23. ^{1}H NMR (400MHz, benzene-*d*$_6$): δ_H 5.30 (t, *J* = 3.6 Hz, 1H), 4.34 (d, *J* = 11.4 Hz, 1H), 4.06 (s, 1H), 3.90 – 3.83 (m, 1H), 3.51 – 3.41 (m, 1H), 3.23 (td, *J* = 6.9, 2.8 Hz, 2H), 3.17 (dt, *J* = 9.2, 6.3 Hz, 2H), 2.89 (dt, *J* = 14.0, 3.4 Hz, 1H), 2.64 – 2.52 (m, 1H), 2.42 (dd, *J* = 13.5, 4.2 Hz, 1H), 2.24 (br. s, 1H), 2.03 (td, *J* = 14.3, 4.6 Hz, 1H), 1.97– 1.74 (m, 5H), 1.68 (s, 3H), 1.64 – 1.55 (m, 3H), 1.50 – 1.13 (m, 11H),1.15 (s, 3H), 1.09 – 0.96 (m, 2H), 1.04 (s, 3H), 0.98 (s, 3H), 0.94 – 0.65 (m, 2H), 0.89 (s, 3H), 0.85 (s, 3H), 0.76 (s, 3H). ^{13}C NMR (101MHz, benzene-*d*$_6$): δ_C 170.7, 155.7, 144.5, 124.1, 72.3, 67.2, 55.7, 48.5, 48.3, 47.8, 47.3, 45.8, 42.3, 41.9, 39.9, 38.6, 37.1, 35.8, 34.1, 33.1, 32.4, 31.0, 26.6_7, 26.6_1, 25.8_4, 25.8_0, 24.2, 24.0, 22.9, 20.5, 18.5, 17.2, 16.0, 12.4. [M+H]$^+$: 583.4476. Calculated for $C_{36}H_{59}O_4N_2$: 583.4469.

A second product, the *bis*-acetate (**MC056**; M_r=624.91 gmol^{-1}), was isolated as a white solid (4.2mg; **R**$_f$ (SiO_2; 1:1 petrol:ether) = 0.38). ^{1}H NMR (400MHz, benzene-*d*$_6$): δ_H 5.29 (t, *J* = 3.6 Hz, 1H), 5.07 (dd, *J* = 11.9, 4.6 Hz, 1H), 4.05 (s, 1H), 4.01 (d, *J* = 11.7 Hz, 1H), 3.91 (d, *J* = 11.6 Hz, 1H), 3.25 (dt, *J* = 9.2, 6.5 Hz, 2H), 3.17 (dt, *J* = 9.1, 6.3 Hz, 2H), 2.91 (dt, *J* = 14.0, 3.4 Hz, 1H), 2.61 (d, *J* = 10.0 Hz, 1H), 2.47 – 2.39 (m, 1H), 2.04 (td, *J* = 14.1, 4.4 Hz, 1H), 1.98 – 1.67 (m, 6H), 1.74 (s, 6H), 1.66 – 1.15 (m, 13H), 1.18 (s, 3H), 1.13 – 0.93 (m, 2H), 1.05 (s, 3H), 0.99 (s, 3H), 0.93 – 0.71 (m, 2H), 0.90 (s, 3H), 0.83 (s, 3H), 0.77 (s, 3H). ^{13}C NMR (101MHz, benzene-*d*$_6$): δ_C 170.1, 169.9, 155.6, 144.6, 123.9, 74.2, 65.5, 55.6, 48.3, 48.1, 47.9, 47.3, 45.8, 41.9, 40.9, 39.9, 37.9, 36.9, 35.8, 34.1, 33.1, 32.3, 31.0, 26.6, 25.8_5, 25.8_2, 24.2, 23.9, 23.4, 22.9, 20.8, 20.4, 18.3, 17.2, 16.0, 13.3.

1-Methyl-3-((4a*R*,6a*R*,6b*S*,8a*S*,14b*R*,16a*S*)-2,2,4a,6a,6b,11,11,14b-octamethyl-4a,5,6,6a, 6b,7,8,9,10,11, 12,12a,14,14a,14b,15,16,16a-octadecahydro-4*H*-piceno[3,4-*d*][1,3]dioxin-8a(4b*H*)-yl)-1-((2*S*,3*R*,4*R*,5*R*)-2,3,4,5,6-pentahydroxyhexyl)urea (MC059**)**

To a solution of *N*-methyl-D-glucamine (0.008 g, 1.05 mol.eq.) in dry DMF (1.6 mL), solid MC015 (0.02 g, 1.0 mol.eq.) was added in one portion. The mixture was heated to

70°C for 0.5 hour, whereupon the DMF was removed under high vacuum and the residue was purified by chromatography (10:1 chloroform:methanol; silica NEt_3 neutralized) to afford the product as a white solid (0.017g; 61%); $\mathbf{R_f}$ (SiO_2; 10:1 chloroform:methanol) = 0.23. ^{13}C NMR (101MHz, methanol-d_4): δ_C 160.0, 144.9, 124.7, 100.4, 78.9, 74.3, 73.5, 73.4, 73.1, 70.5, 64.8, 57.3, 52.7, 52.5, 48.2, 47.9, 47.6, 42.8, 41.0, 39.9, 38.3, 37.9, 36.6, 36.0, 34.5, 33.4, 33.2, 31.7, 30.1, 27.3, 24.5_1, 24.5_0, 24.4_8, 23.9, 19.7, 18.7, 17.9, 17.0, 12.9, 9.2.

4-Methyl-*N*-((4a*R*,6a*R*,6b*S*,8a*S*,14b*R*,16a*S*)-2,2,4a,6a,6b,11,11,14b-octamethyl-4a,5,6,6a, 6b,7,8,9,10,11, 12,12a,14,14a,14b,15,16,16a-octadecahydro-4*H*-piceno[3,4-*d*][1,3]dioxin-8a(4b*H*)-yl)piperazine-1-carboxamide (MC062**)**

To a solution of MC015 (0.024g, 0.047mmol) in dry DCM (1.0mL), 1-methylpiperazine (0.0052g, 0.0057mL, 0.052mmol) was administered in one portion, and the mixture was stirred at RT for 19 hours. The mixture was then concentrated *in vacuo* and the residue was purified by column chromatography (10:1 chloroform:methanol; silica NEt_3 neutralized), to afford the product as a white solid (0.02g; 70%); R_f (SiO_2; 10:1 chloroform:methanol) = 0. 46; ^1H NMR (400MHz, benzene-d_6) δ_H: 5.29 – 5.23 (m, 1H), 4.35 (s, 1H), 3.59 (d, *J* = 10.5 Hz, 1H), 3.48 – 3.30 (m, 6H), 2.92 (dt, *J* = 14.0, 3.4 Hz, 1H), 2.69 – 2.57 (m, 1H), 2.37 – 2.26 (m, 1H), 2.16 – 2.04 (m, 4H), 1.99 (s, 3H), 1.97 – 1.75 (m, 6H), 1.74 – 1.53 (m, 2H), 1.58 (s, 3H), 1.52 – 1.11 (m, 8H), 1.36 (s, 3H), 1.23 (s, 3H), 1.16 (s, 3H), 1.07 – 0.77 (m, 3H), 1.00 (s, 3H), 0.95 (s, 3H), 0.89 (s, 3H), 0.85 (s, 3H), 0.75 – 0.68 (m, 1H); ^{13}C NMR (101MHz, benzene-d_6) δ_C: 156.3, 144.3, 124.3, 99.0, 77.9, 72.7, 55.7, 55.1, 51.9, 48.0_3, 48.0_1, 47.2, 46.1, 44.4, 42.0, 40.0, 39.0, 37.4, 37.1, 35.7, 33.7, 33.2, 32.1, 31.0, 30.4, 26.7, 26.1, 24.2, 24.1, 23.8, 22.5, 19.5, 17.9, 17.1, 16.6, 13.0.

pharmaceutics MDPI

NMR spectra

NMR Spectra S1: Proton and carbon NMR spectra of compounds prepared and evaluated in this work

This section includes data for some known compounds referenced in Table S1.

Run at 400 MHz for proton and 101 MHz for carbon (solvents described in text above for each sample). Carbon spectra are determined using the DEPT-q method.

Compound MC014

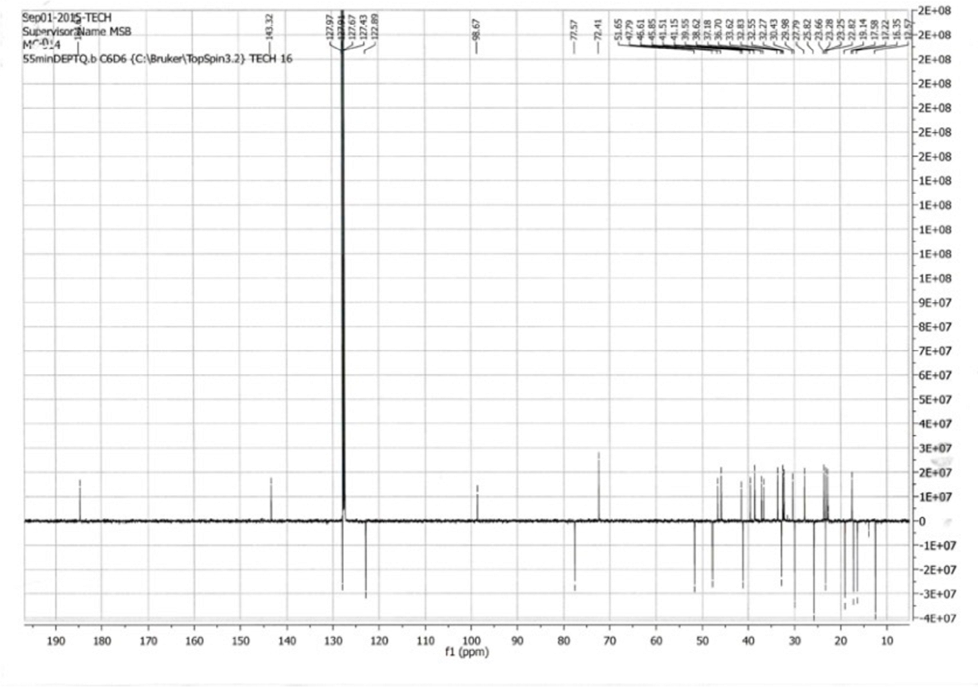

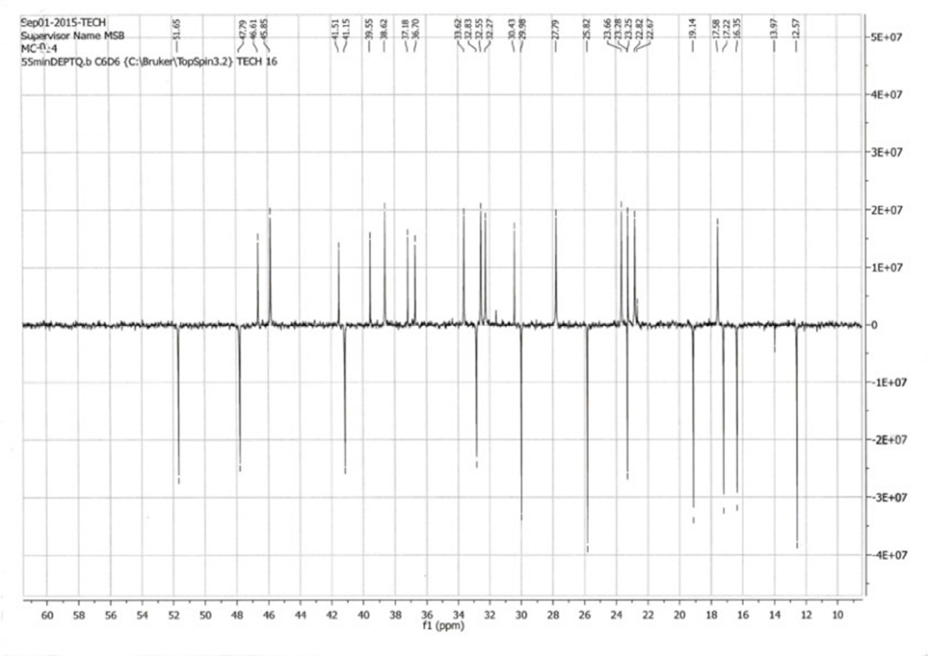

Compound MC015

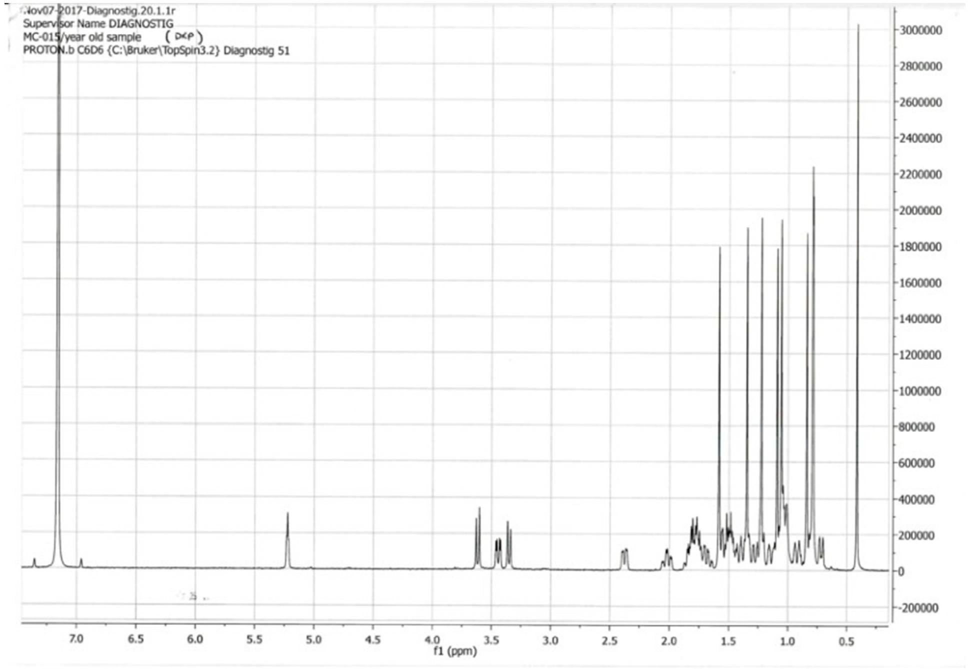

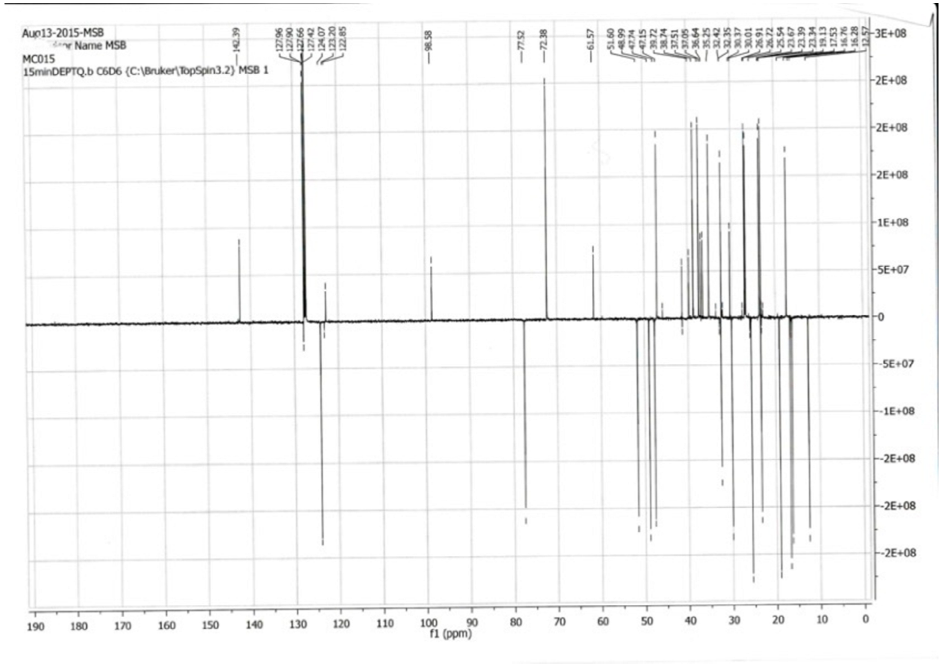

Compound MC019

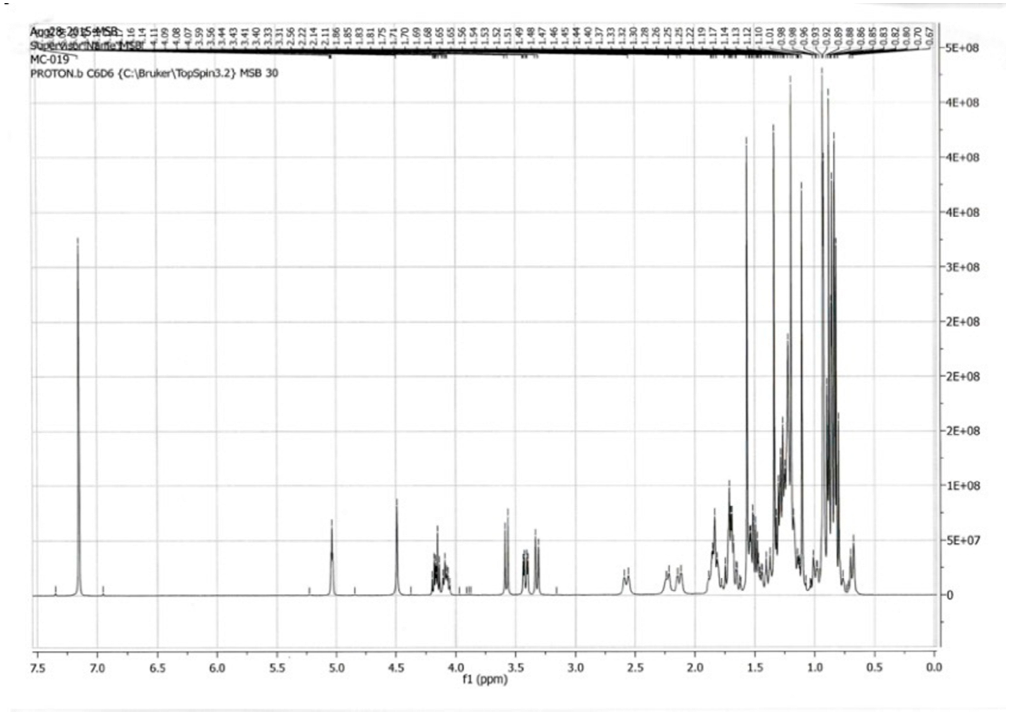

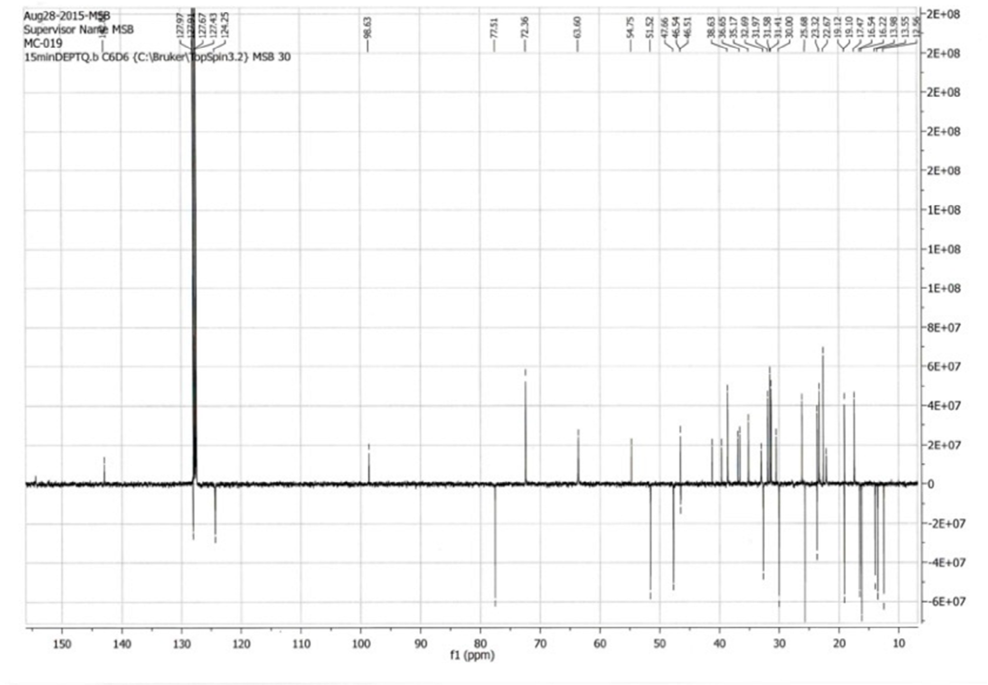

Compound MC022

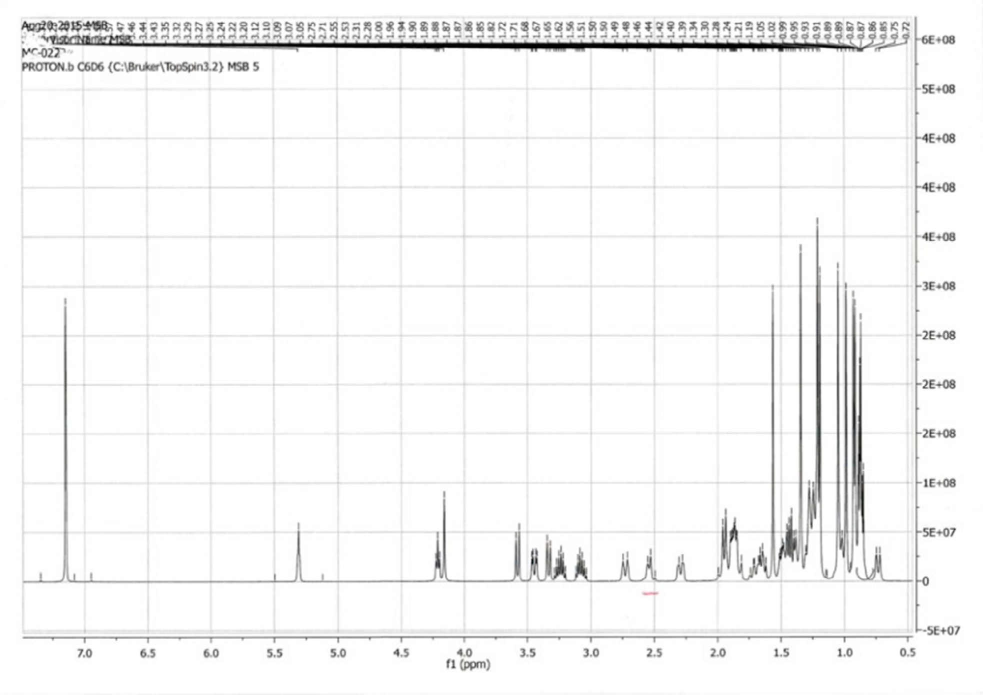

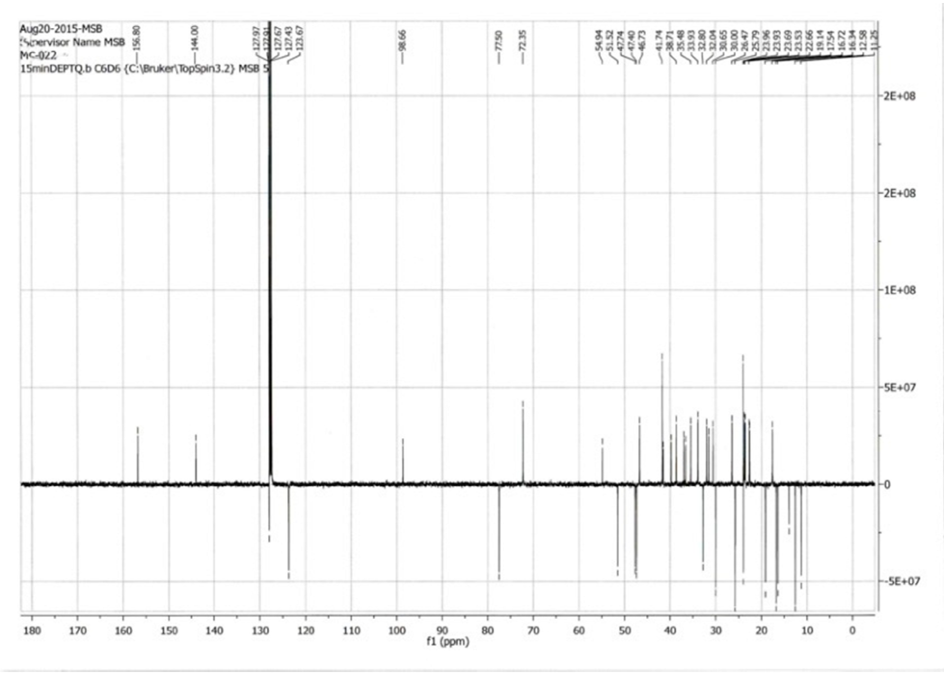

Compound MC023

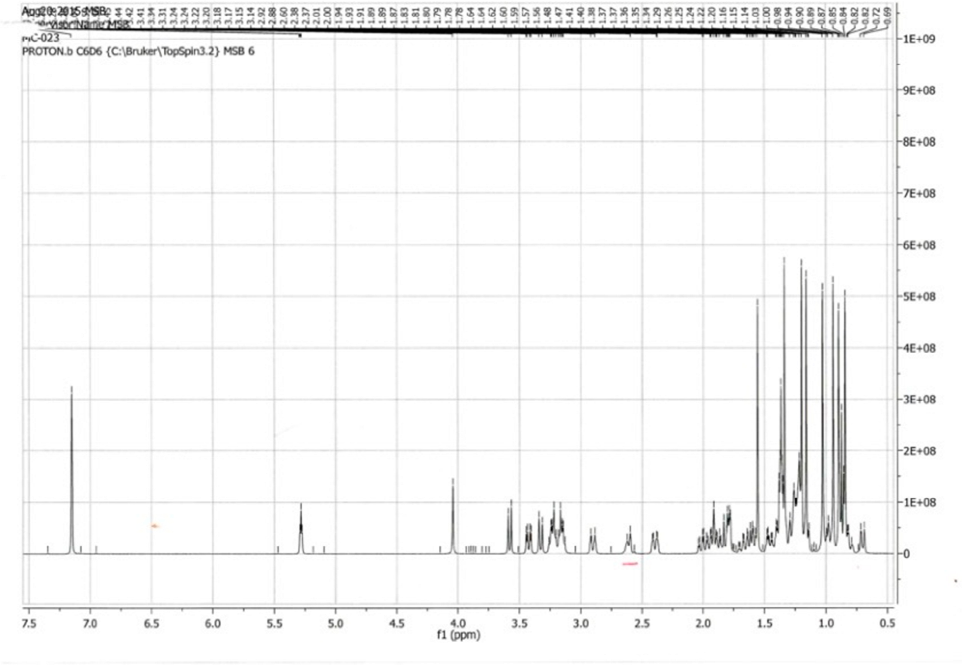

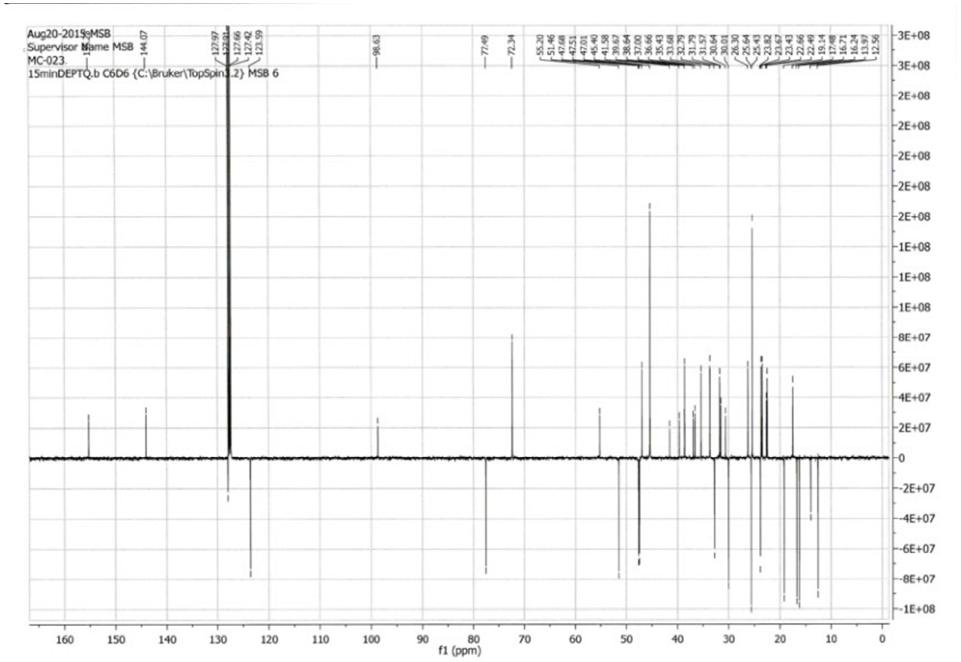

Compound MC024

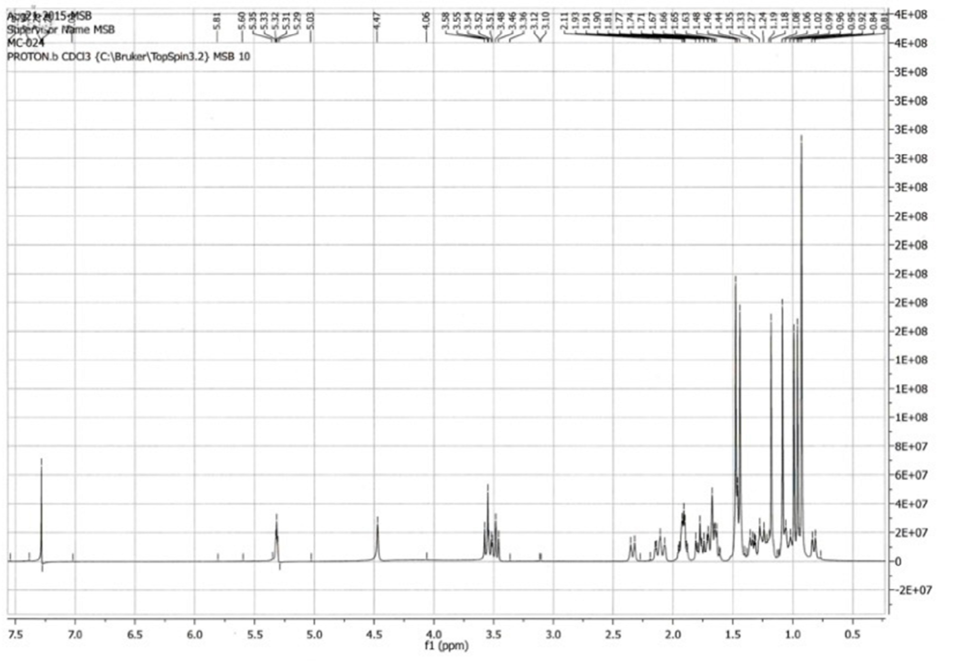

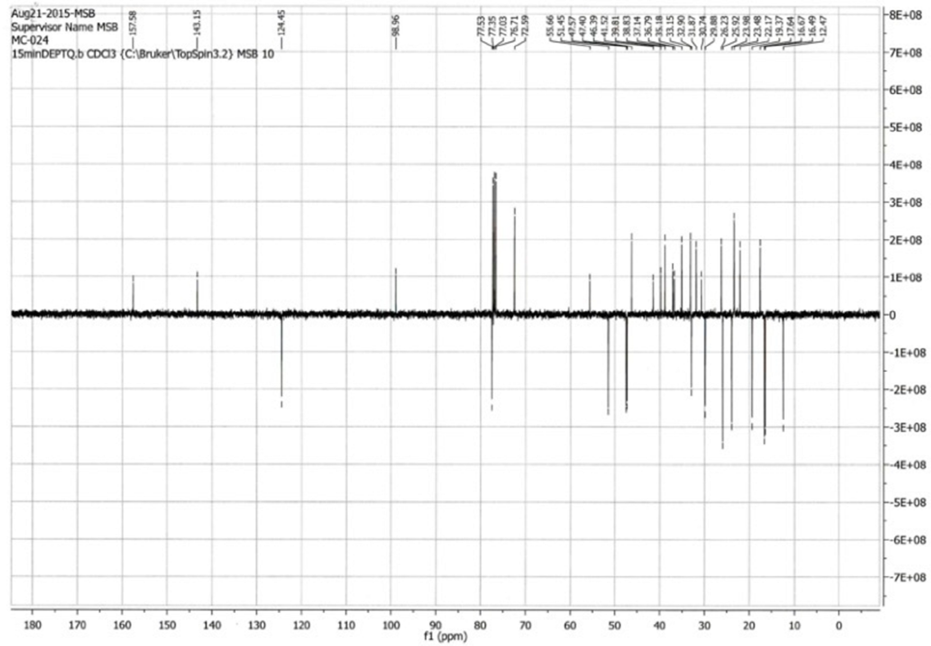

Compound MC027

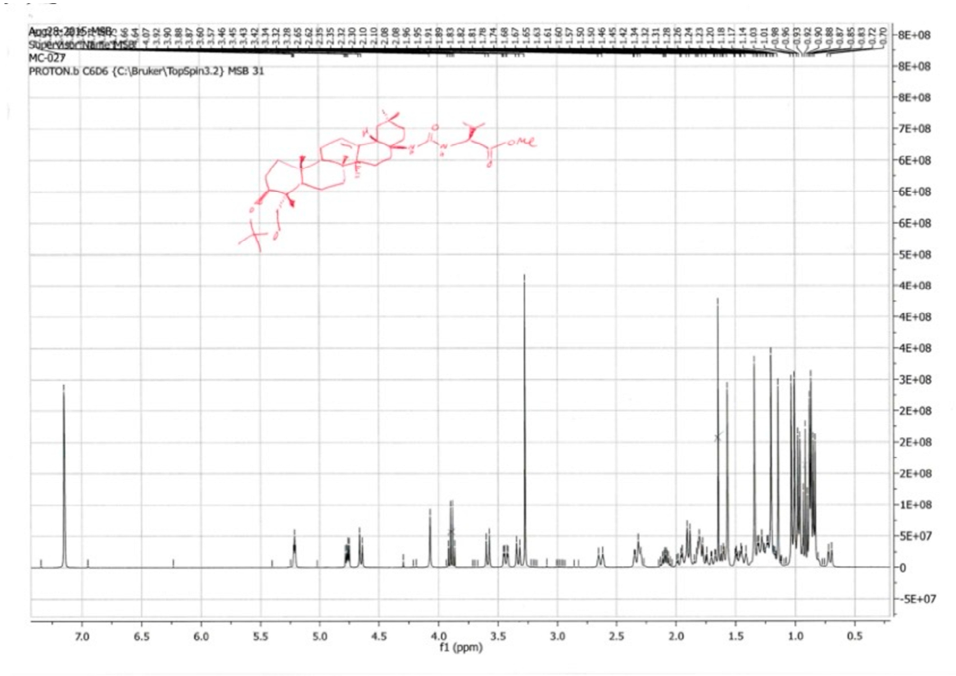

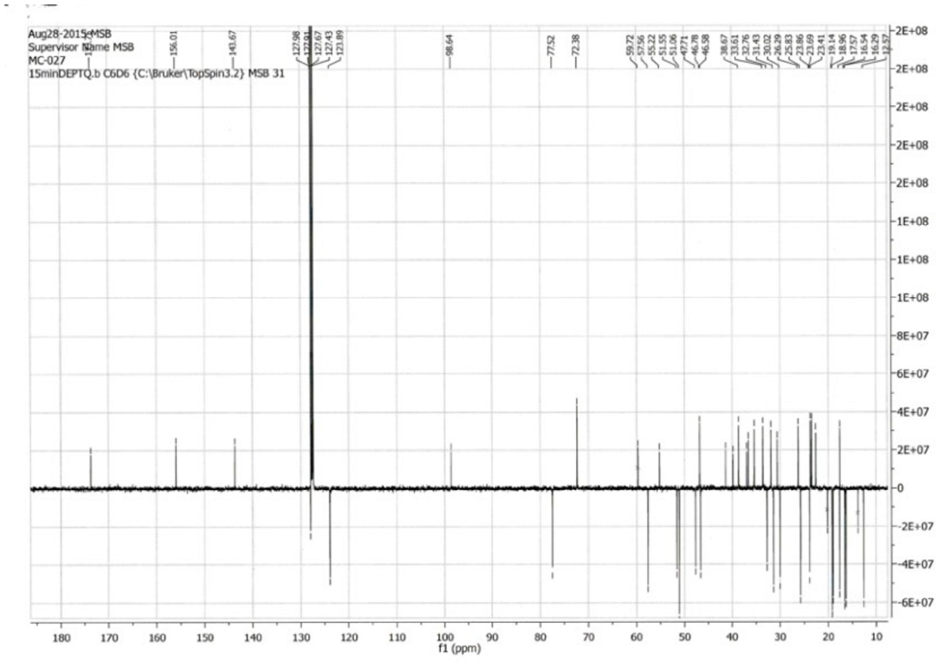

Compound MC028

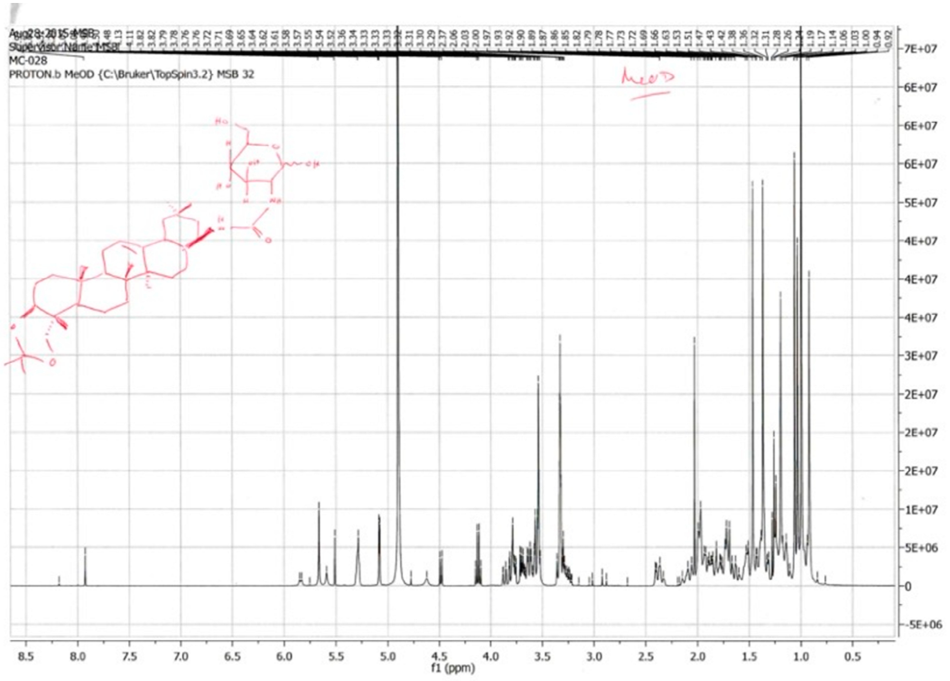

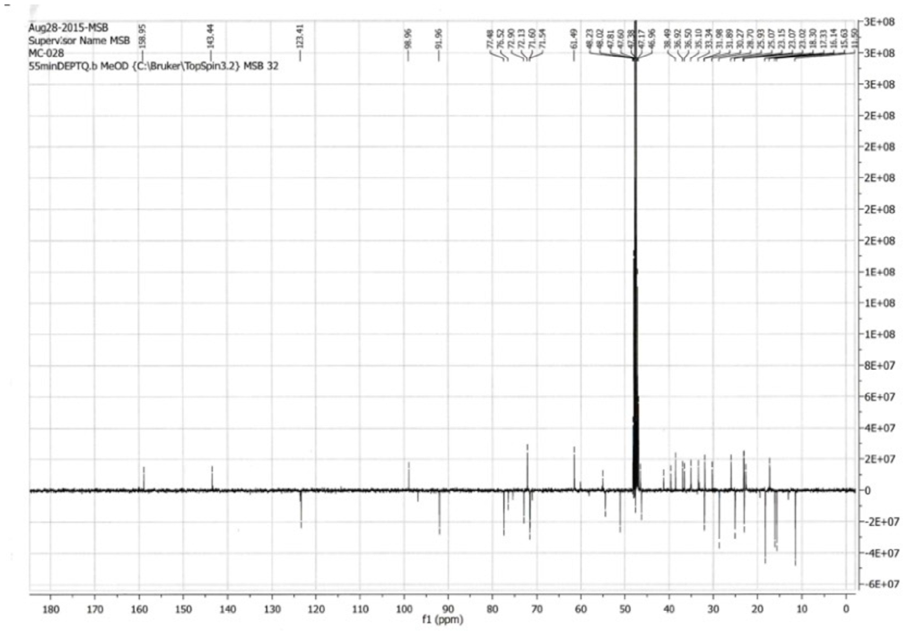

Compound MC030

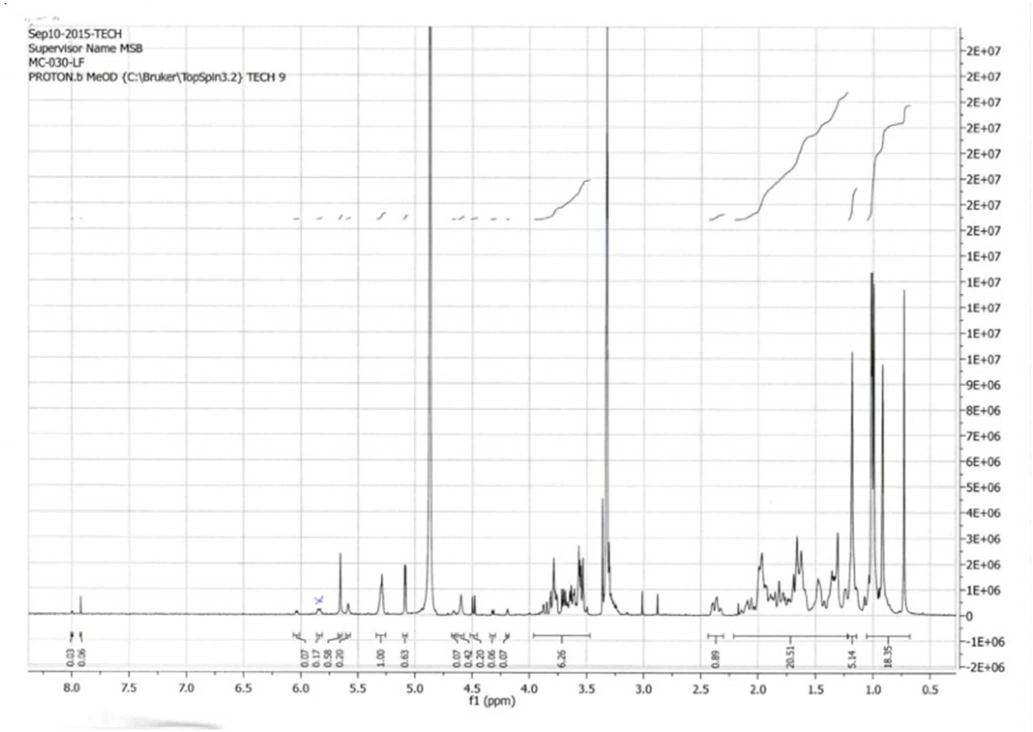

Compound MC031

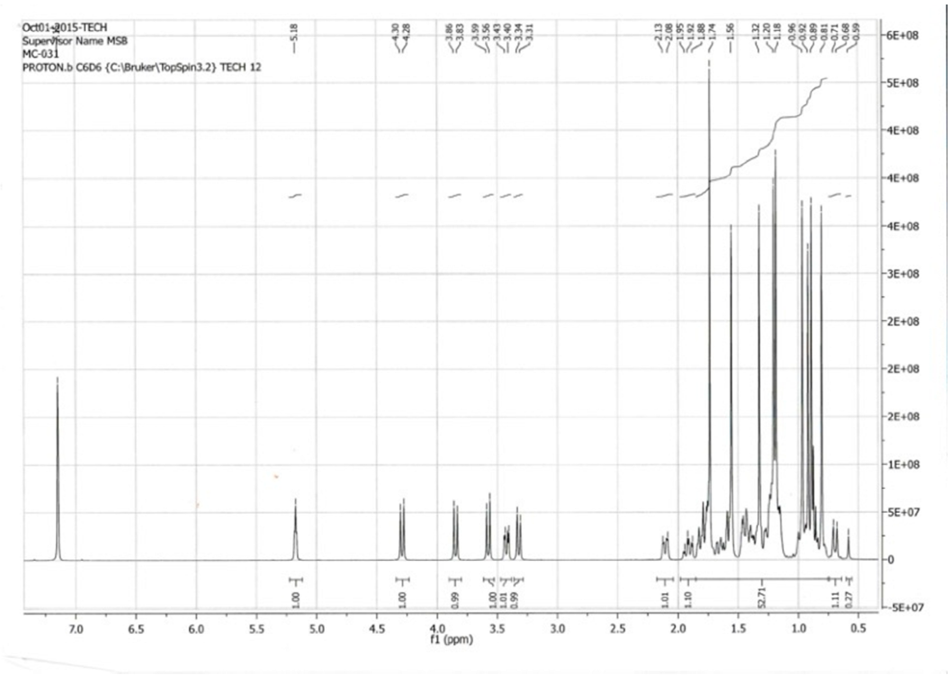

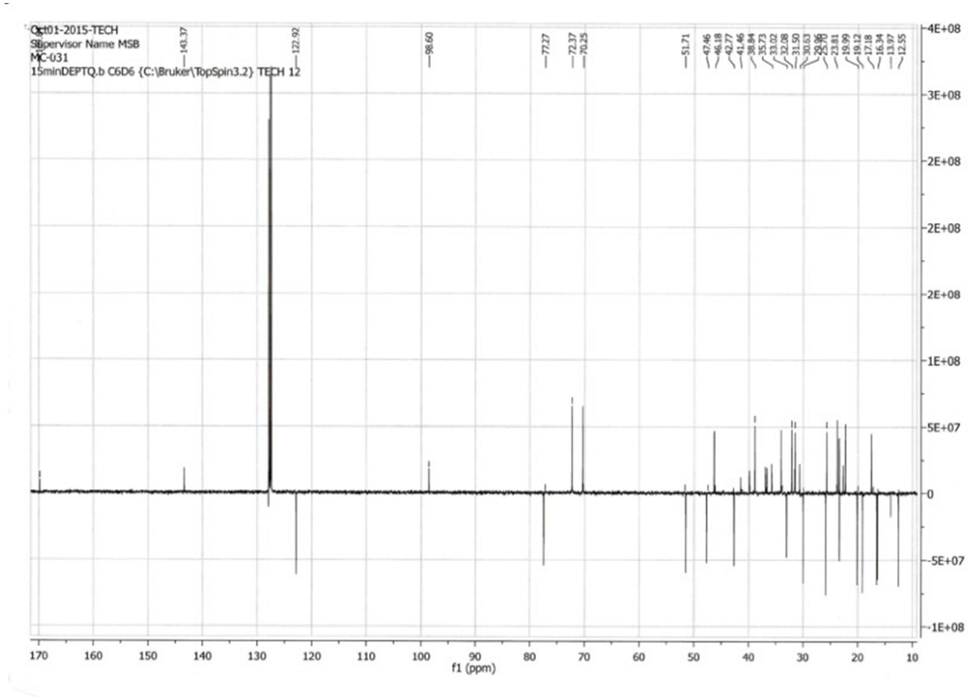

Compound MC032

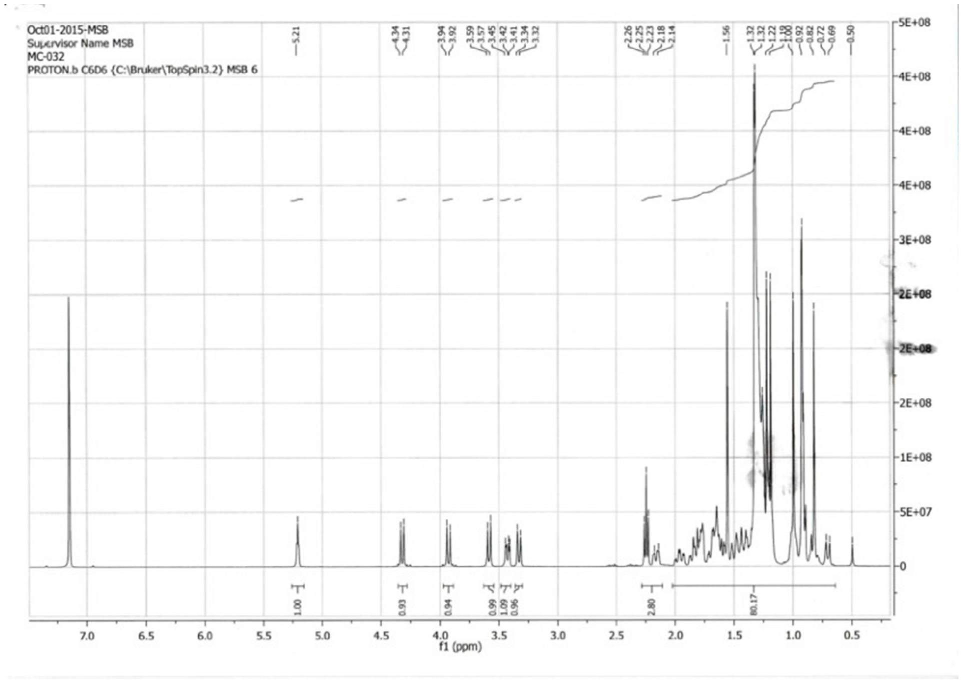

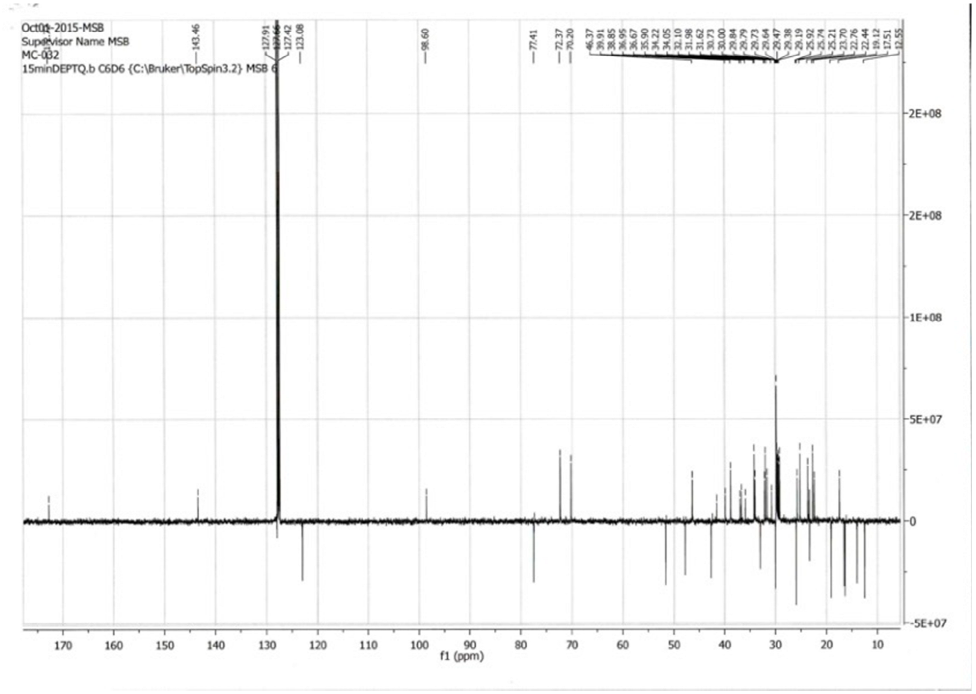

Compound MC033

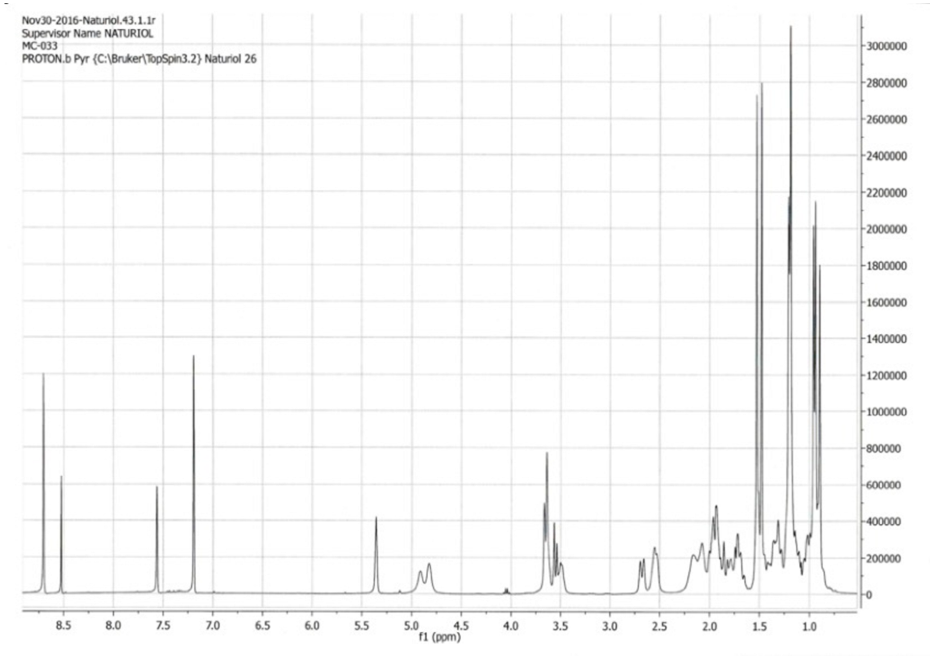

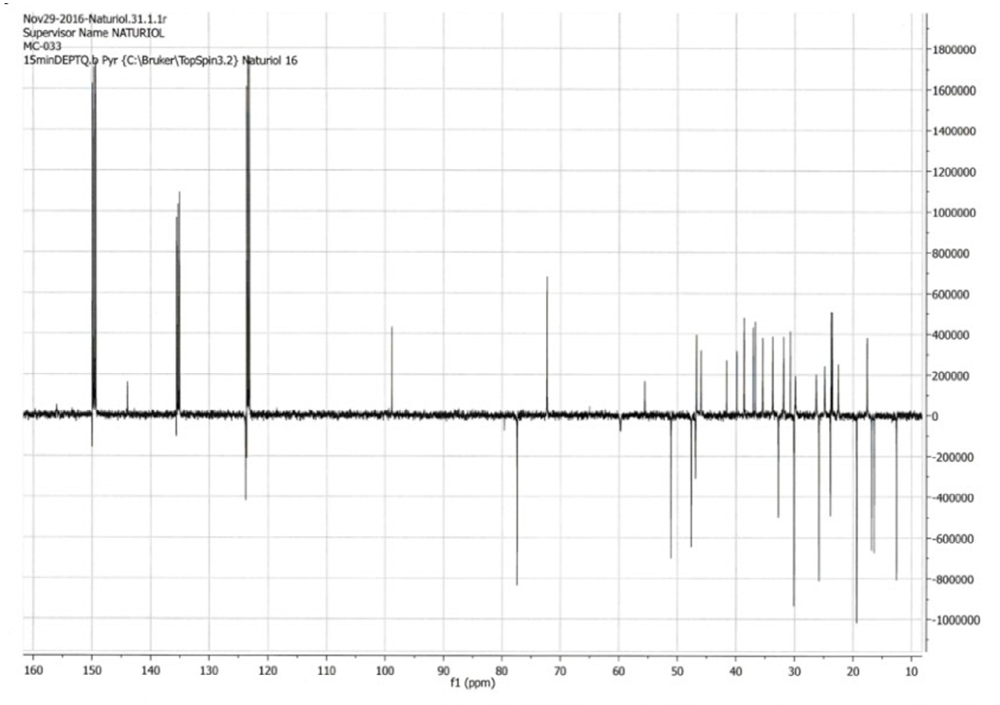

Compound MC034

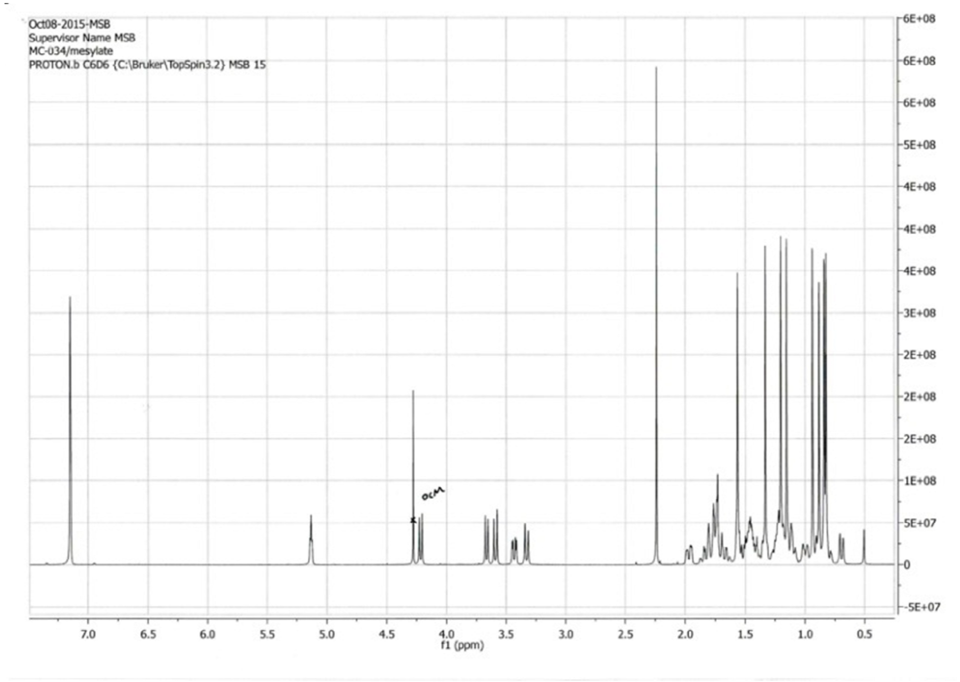

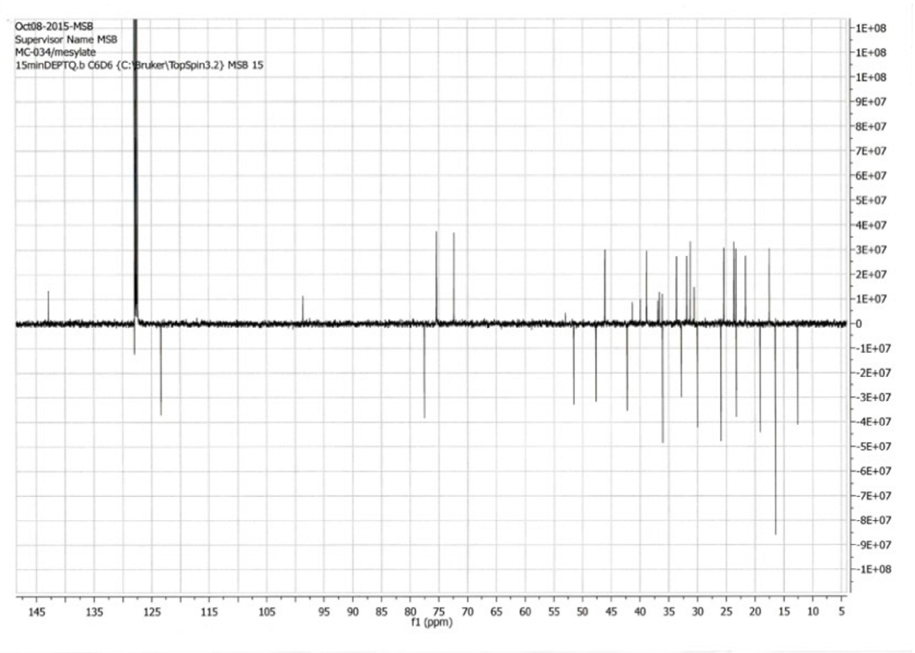

Compound MC037

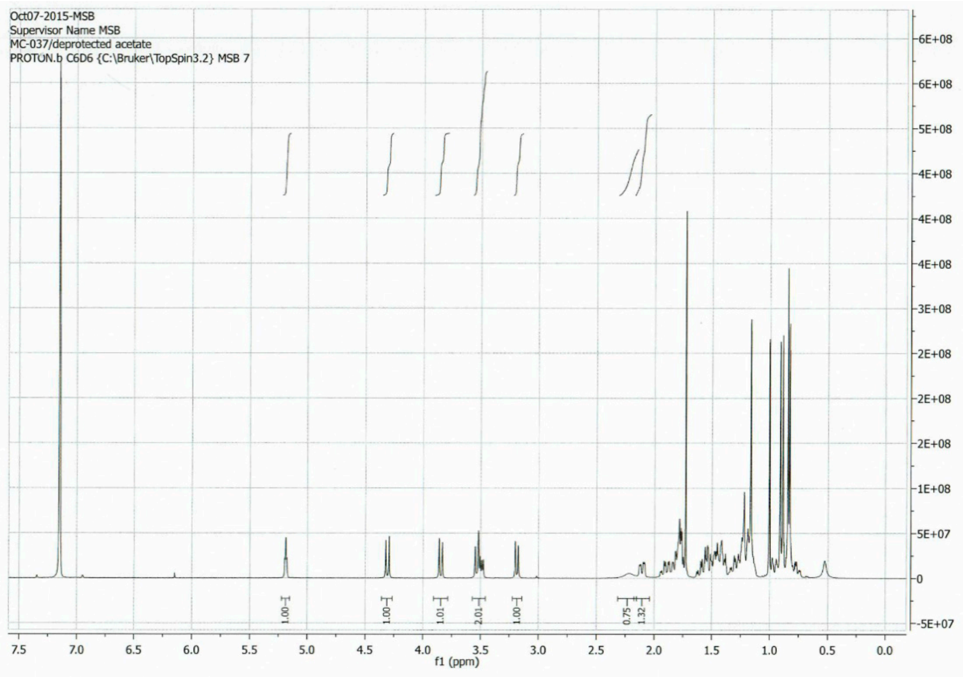

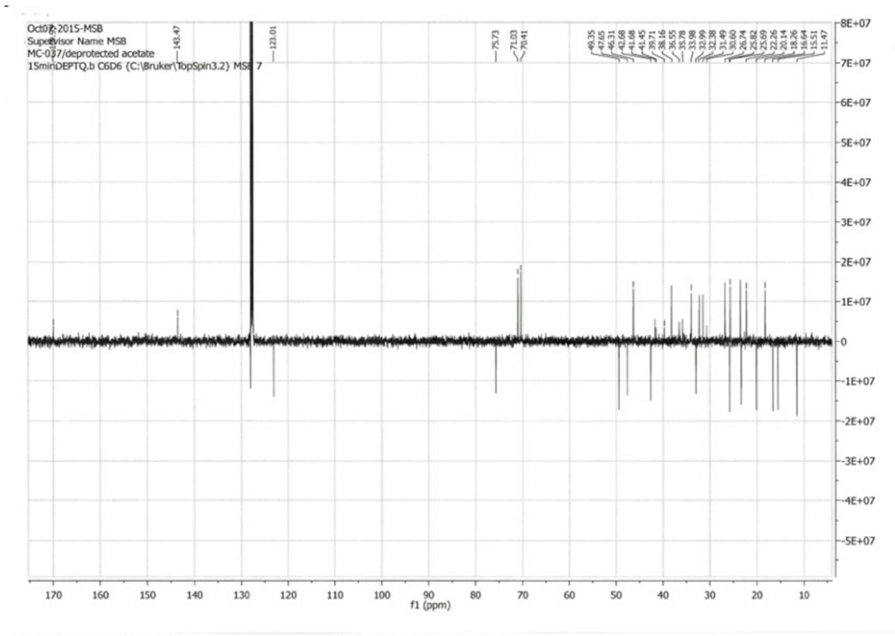

Compound MC038

-

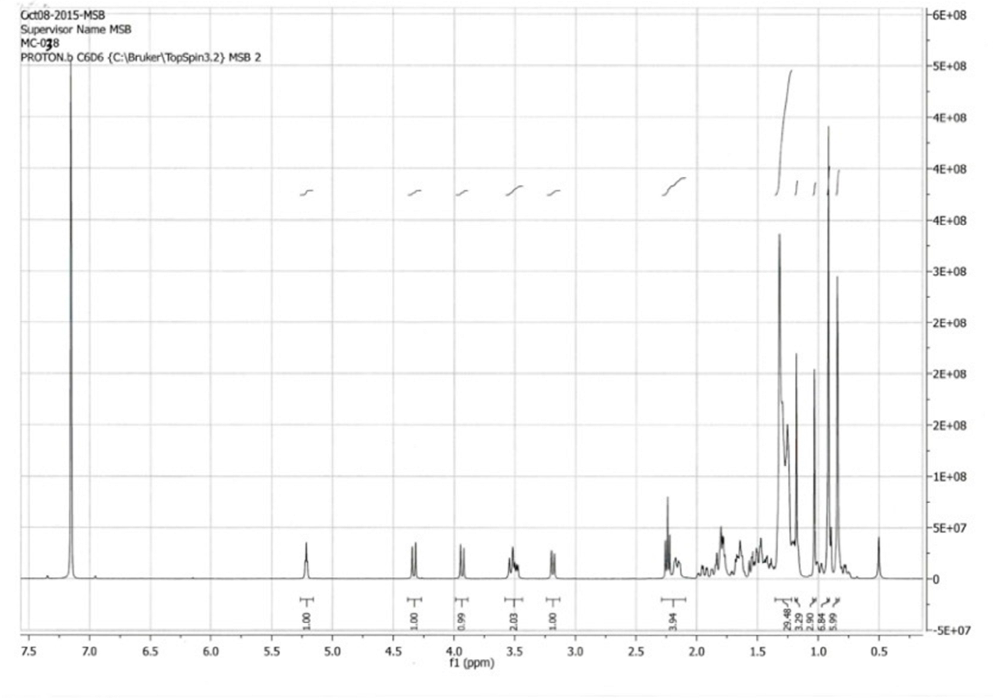

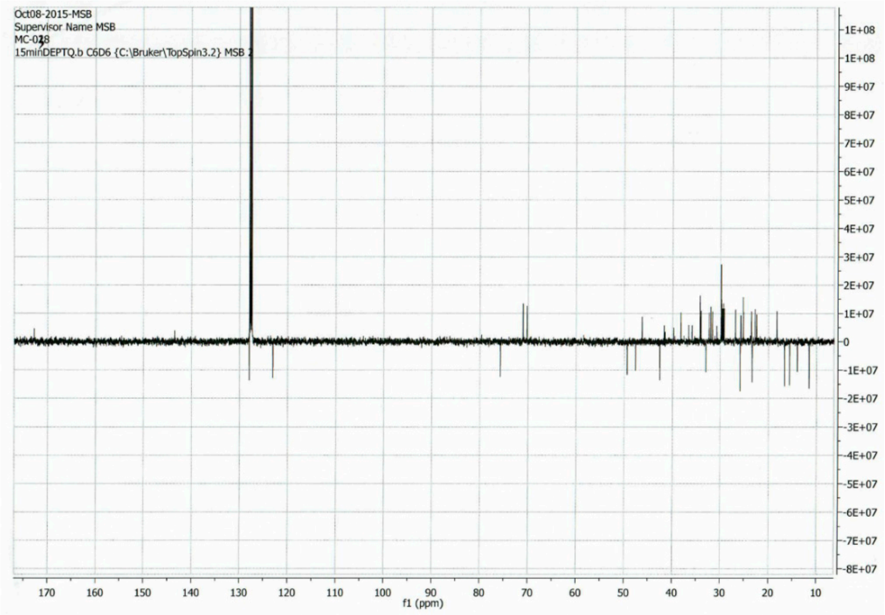

Compound MC042

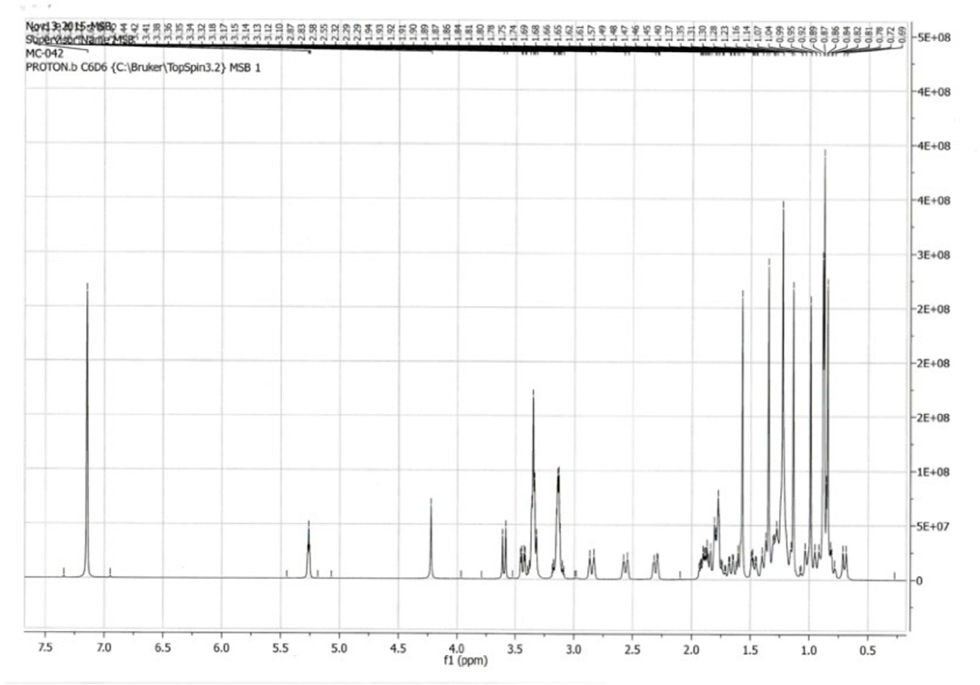

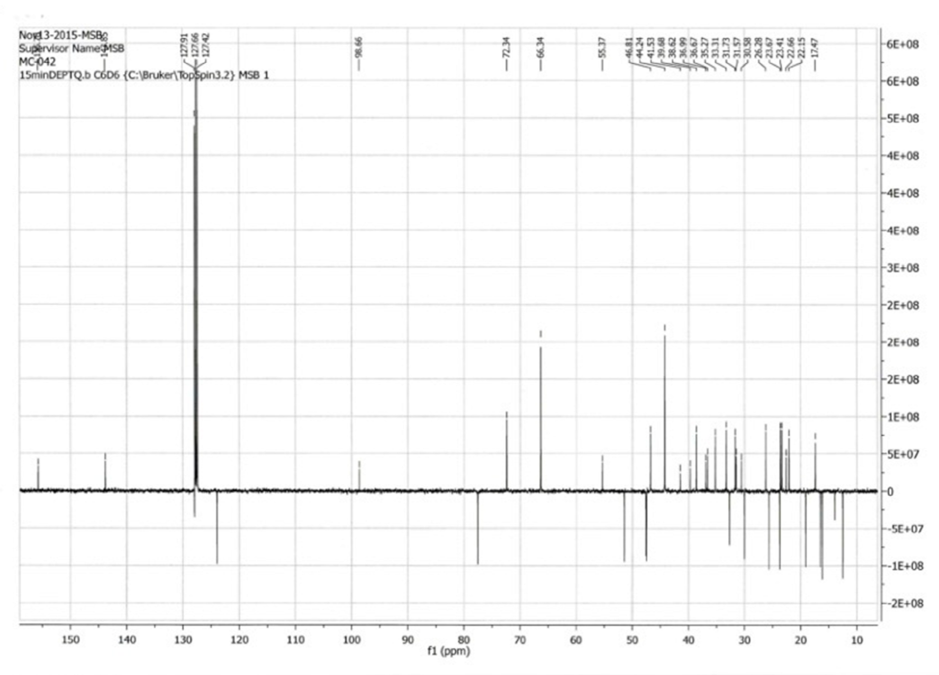

Compound MC044

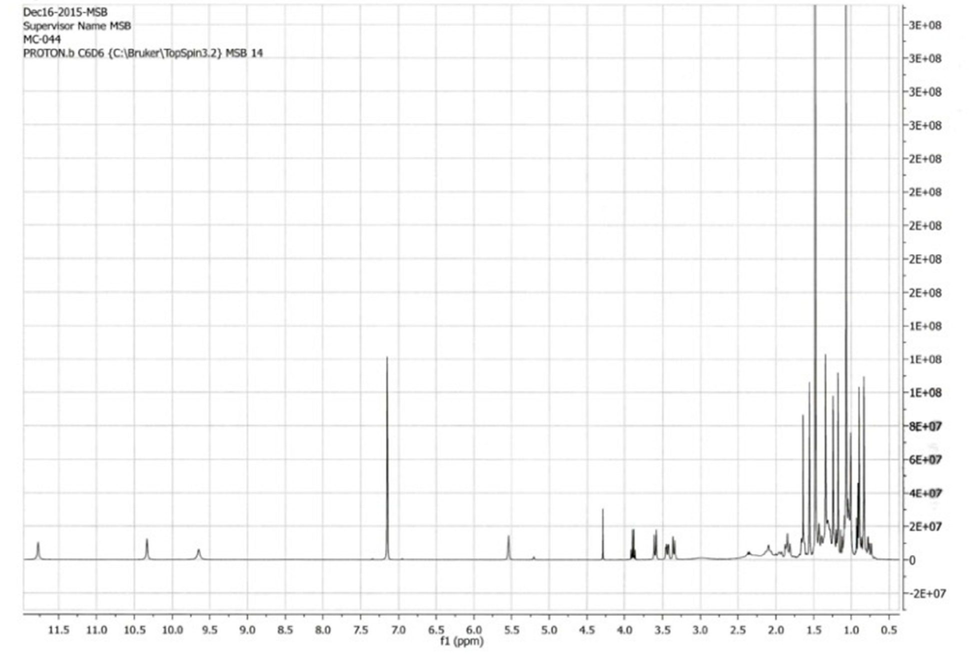

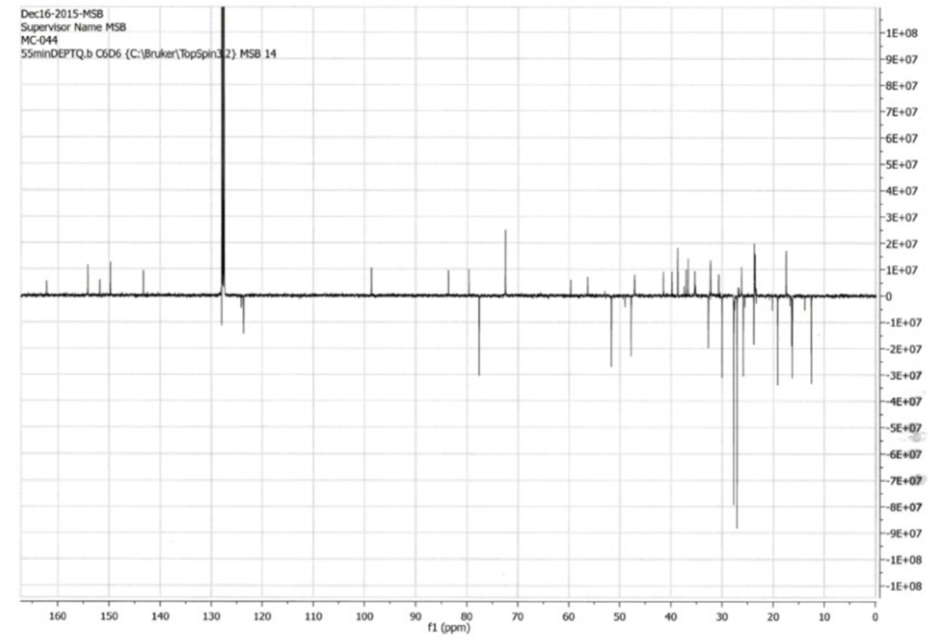

Compound MC055

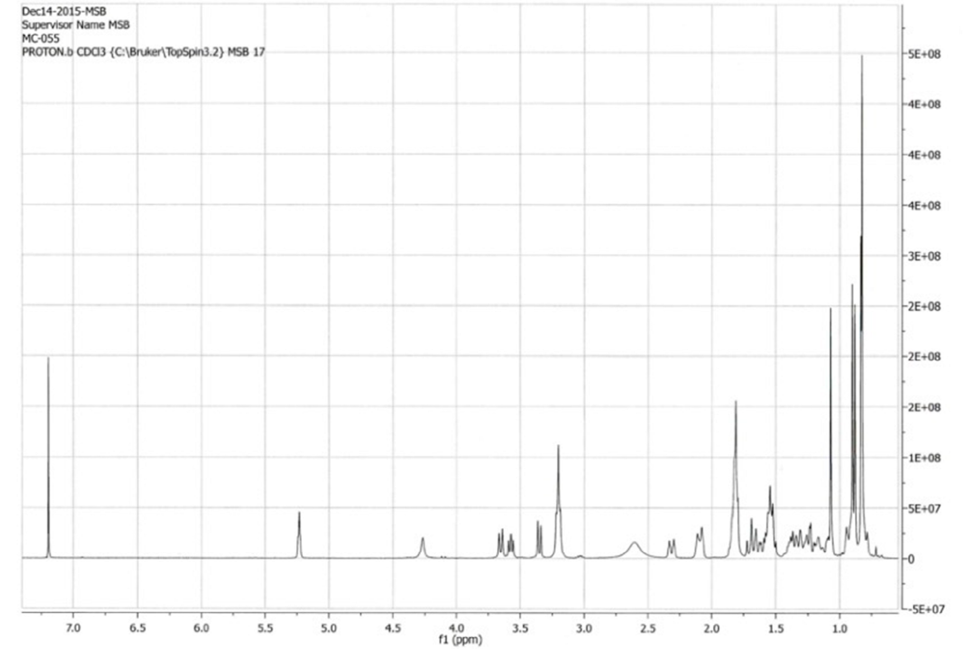

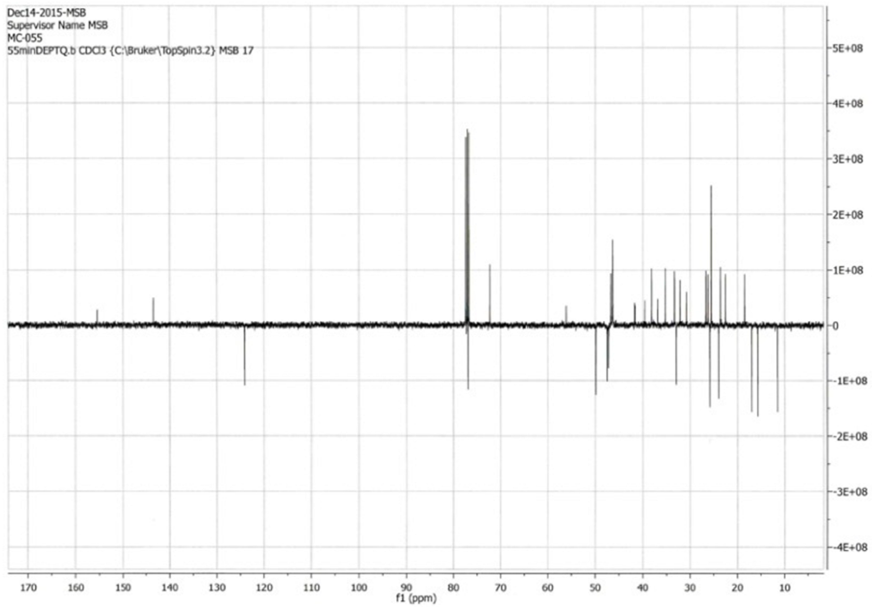

Compound MC057

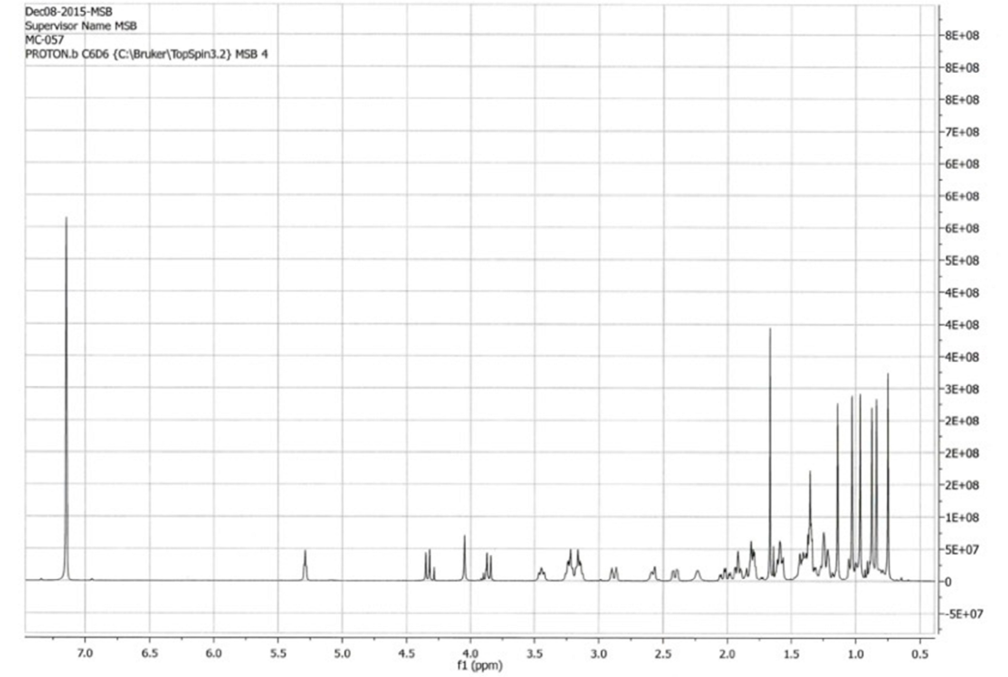

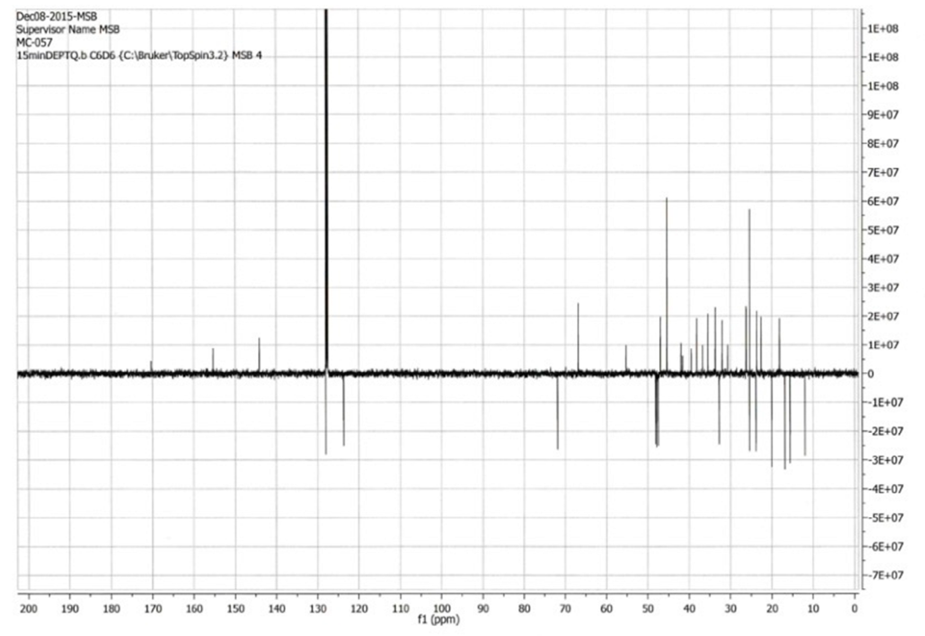

Compound MC059

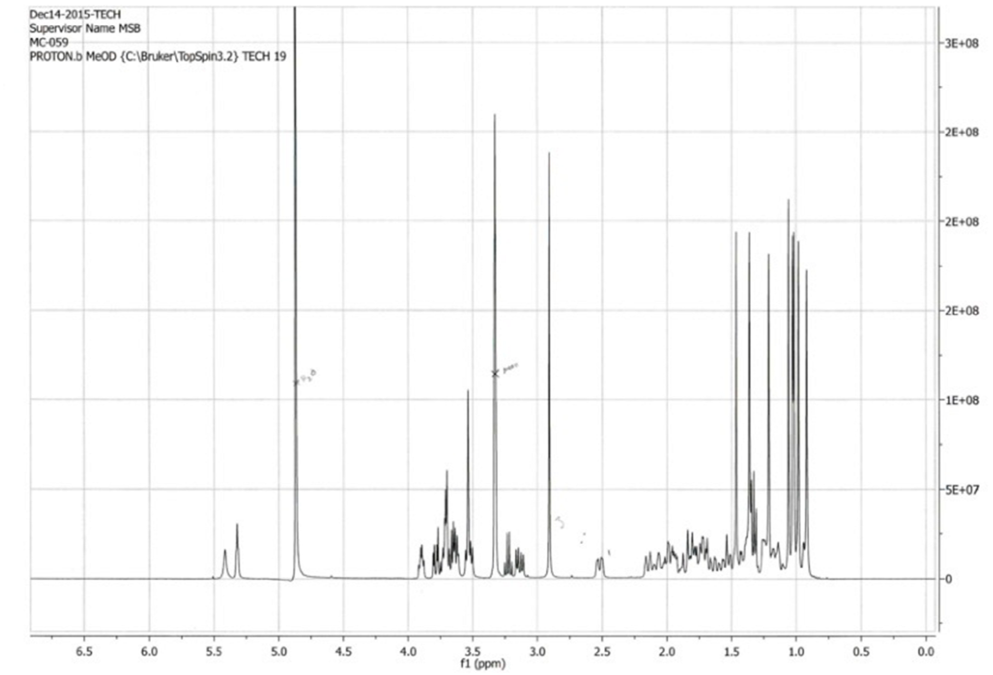

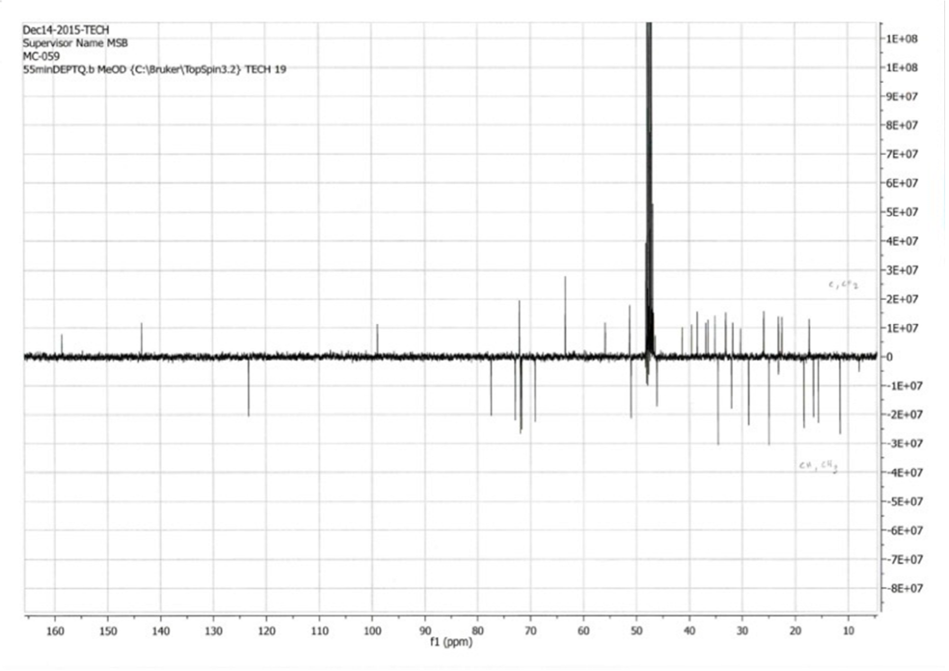

Compound MC062

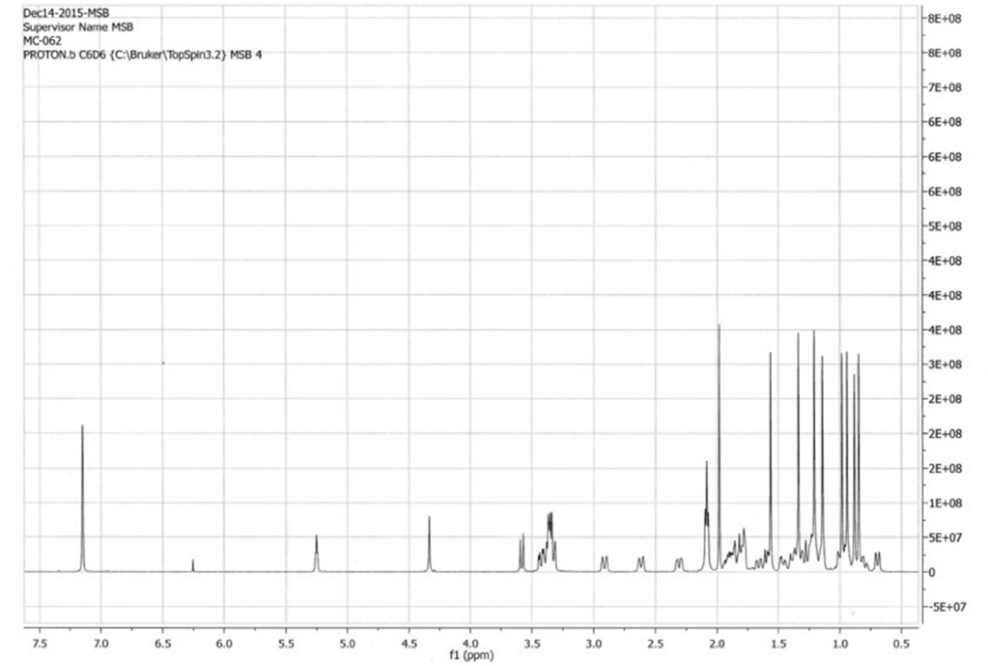

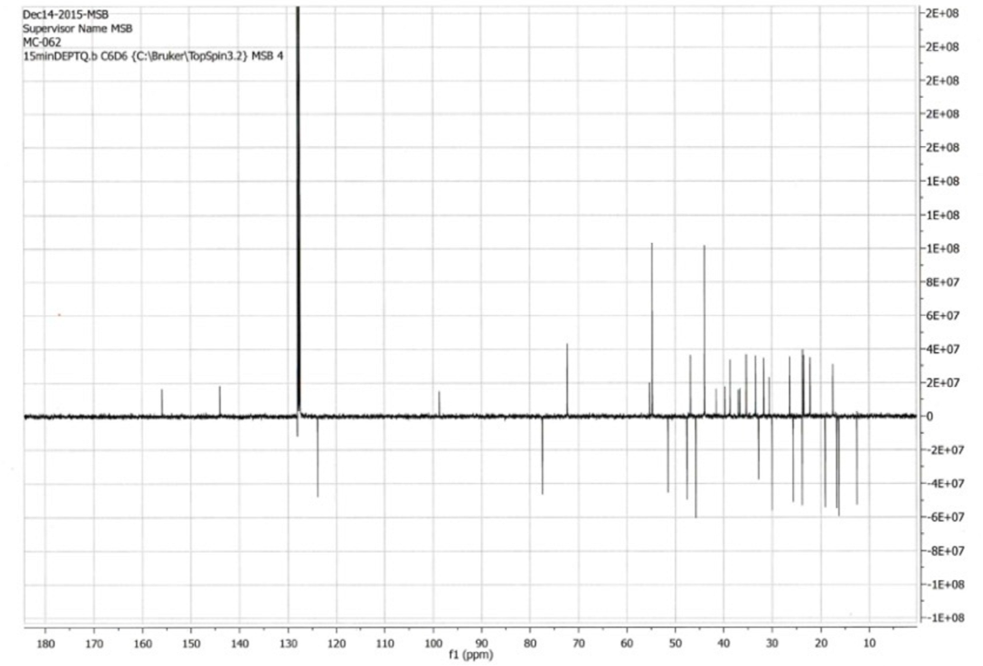

Supplement: Supplementary file 1 [file pharmaceutics-15-01869-s001.zip › pharmaceutics-2434044-supplementary.pdf]
